# Improving the IR spectra alignment algorithm with spectra deconvolution and combination with Raman or VCD spectroscopy†

**DOI:** 10.1039/d2cp04907d

**Published:** 2022-12-22

**Authors:** Lennard Böselt, Roy Aerts, Wouter Herrebout, Sereina Riniker

**Affiliations:** a Laboratory of Physical Chemistry, ETH Zürich Vladimir-Prelog-Weg 2 8093 Zürich Switzerland sriniker@ethz.ch; b Department of Chemistry, University of Antwerp Groenenborgerlaan 171 B-2020 Antwerp Belgium

## Abstract

The relative stereochemistry of organic molecules can be determined by comparing theoretical and experimental infrared (IR) spectra of all isomers and assessing the best match. For this purpose, we have recently developed the IR spectra alignment (IRSA) algorithm for automated optimal alignment. IRSA provides a set of quantitative metrics to identify the candidate structure that agrees best with the experimental spectrum. While the correct diastereomer could be determined for the tested sets of rigid and flexible molecules, two issues were identified with more complex compounds that triggered further development. First, strongly overlapping peaks in the IR spectrum are not treated adequately in the current IRSA implementation. Second, the alignment of multiple spectra from different sources (*e.g.* IR and VCD or Raman) can be improved. In this study, we present an in-depth discussion of these points, followed by the description of modifications to the IRSA algorithm to address them. In particular, we introduce the concept of deconvolution of the experimental and theoretical spectra with a set of pseudo-Voigt bands. The pseudo-Voigt bands have a set of parameters, which can be employed in the alignment algorithm, leading to improved scoring functions. We test the modified algorithm on two data sets. The first set contains compounds with IR and Raman spectra measured in this study, and the second set contains compounds with IR and VCD spectra available in the literature. We show that the algorithm is able to determine the correct diastereomer in all cases. The results highlight that vibrational spectroscopy can be a valuable alternative or complementary method to inform about the stereochemistry of compounds, and the performance of the updated IRSA algorithm suggests that it is a powerful tool for quantitative-based spectral assignments in academia and industry.

## Abstract 2

The relative stereochemistry of organic molecules can be determined by comparing theoretical and experimental infrared (IR) spectra of all isomers and assessing the best match.

## Graphical abstract

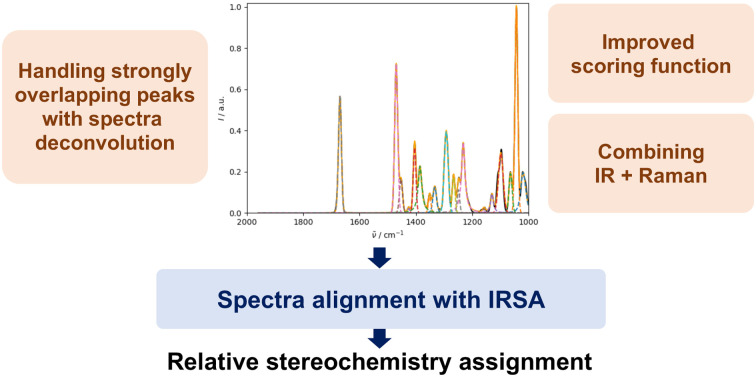

## 1 Introduction

In chemistry, the main workhorse to determine the relative stereochemistry of organic molecules is nuclear magnetic resonance (NMR) spectroscopy. However, NMR spectra can be non-conclusive in the following cases:^1–14^

1. If coupling constants are not resolved.

2. If the spectrum is extremely crowded.

3. If symmetry precludes determination *via* NMR spectroscopy.

4. If NMR signals average out due to fast internal rotation of the compound under investigation.

5. If the substance can only be obtained in low yield.

Vibrational spectroscopy such as infrared (IR), vibrational circular dichroism (VCD), Raman, or Raman optical activity (ROA) spectroscopy can yield valuable additional information in such cases.^6,7^ Vibrational spectroscopy probes the transition moments of vibrations of a compound. This provides complementary information to NMR spectroscopy, which probes the magnetic shielding of atoms. The fingerprint region of vibrational spectra contains information about the compound in a compact manner. The vibrations in this range are non-localized vibrations, which are too complicated to be analyzed without theoretical reference spectra. The general idea of using vibrational spectra to determine the stereochemistry of compounds is thus as follows: Theoretical spectra are generated for all possible isomers, and the computed spectrum that agrees best with the experimental spectrum corresponds most likely to the isomer measured in experiment.^9,14^

This workflow usually involves a number of approximations. First, the computation of the vibrational spectra is generally based on the harmonic frequency analysis. In the harmonic frequency analysis, the potential-energy surface is Taylor approximated around a local minimum structure,1
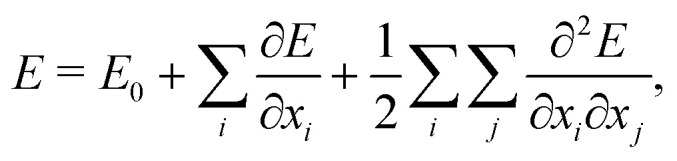
with *x*_*i*_ and *x*_*j*_ being atomic coordinates, and truncated after the second derivatives.^15–17^ The first term describes a constant energy offset. The second term is zero, since the compound is in a local minimum structure. The vibrations are thus completely described by the second derivatives with respect to the atomic coordinates, the Hessian matrix. Anharmonic effects are neglected. This approximation leads to a systematic error, which can be corrected with constant scaling factors. However, the neglect of anharmonic effects also gives rise to a stochastic error, which cannot be corrected with global scaling factors.^15,16^

A second issue results from the sensitivity of the vibrational spectra towards the conformational ensemble. Compounds are often flexible with many rotatable bonds. It is not known *a priori*, which conformers are populated to what degree at room temperature. Hence, an extensive conformational search is necessary, followed by quantum-chemical calculations to estimate the (free) energy of the conformers.^18^ However, the error of density functional theory (DFT)^19^ methods in free-energy calculation is estimated to be 1 kcal mol^−1^,^20^ which is often too imprecise. In addition, the computation is typically performed in vacuum, neglecting intermolecular interactions between solute and solvent atoms. The neglect of these interactions further alters the potential-energy surface such that the second derivatives are perturbed. This may shift the theoretical peaks with respect to the experimental ones. For a review of spectroscopic methods, we refer the reader to ref. 6 and 7.

We recently developed a spectra alignment algorithm for vibrational circular dichroism (VCD) termed VSA^9^ and for infrared termed (IRSA).^14^ The algorithm can partially correct for the stochastic error from the computational setup by interpreting the theoretical and experimental peaks as letters in strings and optimally aligning them. Compared to standard approaches with a global scaling factor, our algorithm allows for local adjustments of individual peaks. Each peak has a set of attributes such as the intensity *I*, the position *x*_0_, and the width *w*. Further, information from multiple spectroscopic sources (*e.g.* VCD and IR) can be combined in the alignment in a straightforward manner. We used the algorithm successfully to determine the correct stereochemical structure not only retrospectively, but also in cases where the correct structure was unknown to us and other methods were unavailable.^14,21^ However, we identified also a few issues with the previous version of the algorithm, which are addressed in this study. The modified algorithm is tested on a set of 14 compounds, for which we measure IR and Raman spectra, and a set of three compounds, for which IR and VCD spectra are available in ref. 18. We show that the algorithm is able to determine the correct diastereomer in all cases.

This article is structured as follows: In Section Theory, we briefly summarize the theory behind the sequence alignment algorithm and its application to vibrational spectroscopy. In Sections 2.1–2.3, we discuss the challenges with the current implementation and the adaptations introduced in this study to address them. In Section Methods, the computational and experimental details are given, followed by Results and Discussion. Finally, we summarize our findings and draw conclusions.

## 2 Theory

In bioinformatics, the Needleman–Wunsch algorithm,^22^ the Smith–Waterman algorithm,^23^ and the basic local alignment search tool^24^ have been developed to optimally align DNA/RNA and amino acid sequences. The foundation of these algorithms is a dynamic programming technique,^25^ where the complete problem (which is hard to solve computationally) is divided into smaller subproblems with decreasing difficulty. The smallest subproblem has a trivial solution, and larger subproblems are dependent on the solution of the smaller subproblems. Thus, the complete problem can be reconstructed by first solving the easier subproblems, reducing the computational complexity significantly. The algorithm aligns two strings by either matching letters to each other (*e.g.* adenine with adenine), mismatching them (*e.g.* adenine with cytosine), or introducing gaps in one of the strings (*e.g.* assign adenine to a gap). The order of the letters in the strings is thereby strictly preserved.

The main ingredient in these alignment algorithms is the scoring function, which measures the “cost” of a match, mismatch, or potential gap between two letters with a score. For example, if two DNA sequences should be aligned, a mismatch between adenine and guanine is penalized, while a match between cytosine and cytosine has a positive contribution to the score. This score is maximized. Hence, the final alignment is sensitive towards the chosen scoring function.

We adapted the idea of the sequence alignment to vibrational spectroscopy,^9,14^ where the peaks in the spectra are the letters in the strings (see Fig. 2 in ref. 9 for a schematic illustration). The approach can in principle also be used for other spectroscopic (*e.g.* NMR) or spectrometric methods (*e.g.* mass spectrometry). In the VSA/IRSA algorithms, each peak is interpreted as a letter with a set of attributes, *e.g.*, the peak position (*x*_0_) and the peak intensity (*I*). These attributes can be incorporated in the scoring function. The algorithm then automatically assigns all theoretical peaks to the experimental peaks, and can additionally shift the theoretical spectrum onto the experimental one. In the current version,^9,14^ the shift is performed by extracting the intensities of the theoretical peaks, and simply “reconvolute” them at the position to which they were shifted to. The alignment is within the framework of the chosen scoring function optimal. An issue arises if the theoretical spectrum has more peaks than the experimental spectrum, thus leaving theoretical peaks unassigned. We solved this by shifting the unassigned peaks by the same distance as the closest assigned theoretical peak. The scoring function used in ref. 14 is of the form,2
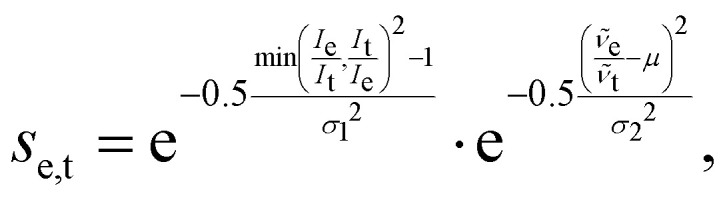
where *I*_e_ and *I*_t_ are the intensities of the peaks in the experimental and theoretical spectra, respectively, *ν̃*_e_ and *ν̃*_t_ are the wave numbers at which the peaks appear in the spectra, and *σ*_1_, *σ*_2_ and *μ* are fitted parameters, which depend on the computational method chosen. The first term computes a score contribution of the intensities, whereas the second computes a score contribution of the frequencies.

After the alignment is performed, quantitative metrics such as the Pearson correlation coefficient^26^ and the Spearman correlation coefficient^27^ can be computed to estimate the goodness of the match. It is also possible to calculate other metrics.^28^ Further, the alignment provides the total score *s* of the alignment procedure, which measures how similar the experimental and theoretical spectra are within the framework of the scoring function (*i.e.* how much “work” was needed to align the spectra). This additional metric was found to be highly important to distinguish the correct isomer.^14^ Provided that a suitable scoring function is available, the total score can be solely used to determine the correct stereochemistry.

In the following, three issues with the current implementation are discussed and possible avenues to resolve them are described.

### 2.1 Handling strongly overlapping peaks

**Fig. 1:**
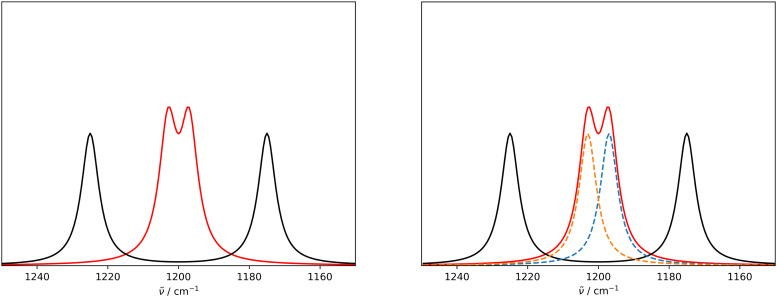
(Left) Illustrative example of a pair of non-overlapping peaks (black) and strongly overlapping peaks (red) with the same individual intensity and Lorentzians. The current IRSA/VSA algorithms use the effective intensities of the red peaks in the scoring function, which are higher than the true ones. (Right) Same peak pairs, overlaid with the decomposition of the red peaks (blue and orange dashed lines) obtained by fitting two Lorentzians to the red spectrum simultaneously. The modified IRSA algorithm uses the intensities of the deconvoluted peaks in the scoring function.

An issue with the current implementation of the IRSA/VSA algorithms arises for spectra with strongly overlapping peaks. Consider the two Lorentzian broadened peaks in the left panel of Fig. 1. The black line represents the (experimental) spectrum, to which the red (theoretical) spectrum should be aligned to. The red and black spectra assume the same Lorentzians, but the red peaks are closer to each other than the black peaks. Even though the red peaks are only shifted on the *x*-axis, they have a higher effective intensity due to a considerable overlap. If the two red peaks are correctly matched by the algorithm to the two black peaks and shifted accordingly, their intensity should decrease since the overlap is reduced. However, the current version of the algorithm does not account appropriately for this case, *i.e.* the red peaks keep the initial (inflated) intensity after shifting.

This issue can be resolved in the following manner. In the case that two peaks strongly overlap, the underlying peaks can be extracted by fitting a set of Lorentzians to the spectrum *via* a least square procedure. The right panel of Fig. 1 shows the two underlying Lorentzians (dashed lines) that were recovered by fitting them to the red spectrum simultaneously. The intensities of the fitted Lorentzians present a more realistic estimate of the true intensities of the underlying peaks. The estimated intensities can subsequently be used when shifting the peaks to align the spectra. In practice, Lorentzians are often too simple to fit the experimental spectrum, since they only capture the life time broadening of the peaks and cannot describe the perturbed shape due to peak overlap. A more sophisticated alternative is the usage of a pseudo-Voigt bandshape *V*,^29^ which is a linear combination of a Lorentzian *L* and a Gaussian bandshape *G*,

3 *V* = *η*·*L* + (1 − *η*)·*G*,

with4
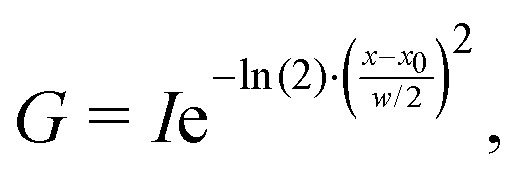
where *η* ∈ [0…1] is a mixing parameter. Eqn (4) is the Gaussian broadening process. Pseudo-Voigt bandshapes can account for more broadening processes, such as pressure broadening, and provide generally more flexibility due to a higher number of parameters. Hence, pseudo-Voigt bandshapes are more suitable to fit theoretical and experimental spectra.

The updated alignment algorithm works as follows: (1) the theoretical spectrum and the experimental spectrum are deconvoluted by assuming a set of pseudo-Voigt bands.^29^ The number of pseudo-Voigt bands is equal to the number of (manually or automatically) detected peaks. From the number of pseudo-Voigt bands, we obtain the peak position *x*_0_, the peak height *I*, the bandwidth *w*, and a mixing parameter *η*. (2) The set of theoretical pseudo-Voigt bands is aligned to the set of experimental pseudo-Voigt bands. Unassigned theoretical peaks are shifted by the same distance as the closest assigned theoretical peak. The pseudo-Voigt bands can now be re-convoluted using the information obtained in step (1). This modified algorithm can handle overlapping peaks better as the intensities of these peaks are no longer inflated after shifting.

Furthermore, the fit of the experimental and theoretical spectrum using pseudo-Voigt bands provides a set of parameters for each peak, which can be used in the scoring function. In the next section, we discuss how an adapted scoring function can leverage these attributes.

### 2.2 Improving the scoring function

The scoring function given in eqn (2) performs well when the number of peaks is similar across all possible isomers of a compound. However, the scoring function gives only zero when peaks are at infinite distance. Thus, peaks might be shifted too much if they are at the lower or higher end of the spectrum.

This point can be addressed as follows. The introduced deconvolution of the experimental and theoretical spectra yields the bandwidth of the peaks. This quantity contains information about the density of the spectrum, which can be incorporated into the scoring function. We propose the following procedure. First, the theoretical spectrum is scaled by the constant factor *μ*, which can correct for the systematic error in the harmonic approximation and is determined by the level of theory used. Second, the alignment algorithm is carried out using the following set of three modified scoring functions with cutoffs for the difference between the experimental and theoretical peaks in intensity, position, and/or bandwidth. This means that two peaks are only matched if all quantities are within the cutoffs. Correspondingly, the score between two peaks is zero if one of the parameters is zero. Hence, a high intensity peak cannot be matched to a low intensity peak, even if they appear at similar frequencies.5
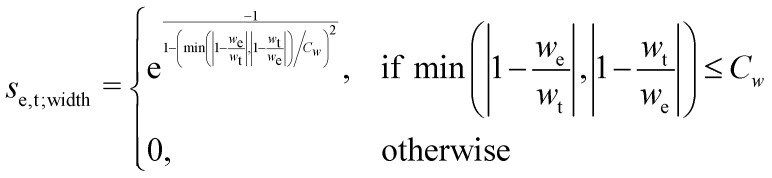
6
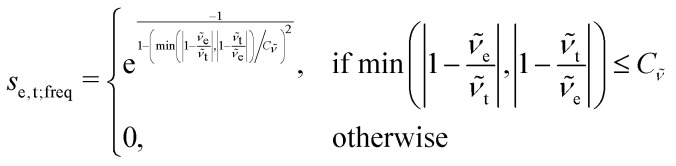
7
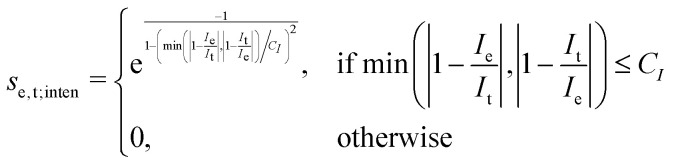
where *w*_e_ and *w*_t_ are the bandwidths extracted from the pseudo-Voigt fit for the experimental and theoretical spectrum, respectively, *I*_t_ and *I*_e_ are the intensities, and *ν̃*_e_ and *ν̃*_t_ the wave numbers. *C*_*w*_, *C*_*I*_, and *C*_*ν̃*_ are the cutoff parameters. The general functional form,8
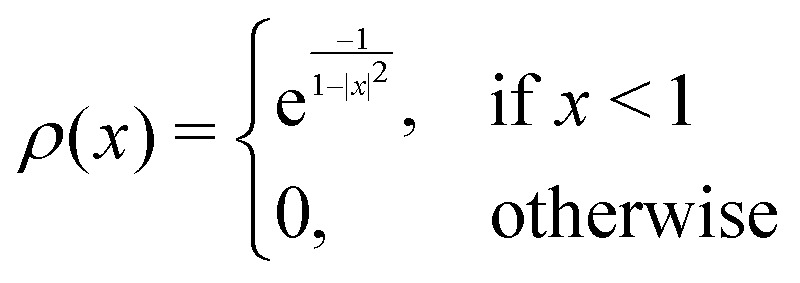
is a well known mollifier.^30^

The score between two peaks is then computed as,

9 *s*_e,t_ = *s*_e,t;inten_·*s*_e,t;width_·*s*_e,t;freq_,

and the final score is maximized using10
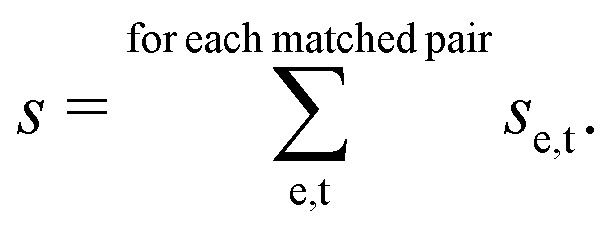
The scoring function gives a maximum value of 1.0 for each pair of peaks and converges smoothly to zero. Furthermore, it can be tuned very precisely, *i.e.* peaks that deviate too much in either position, bandwidth or intensity are not matched and do not contribute to the final score. Note that each peak contributes equally to the final score, *i.e.* high-intensity peaks are valued the same as low-intensity peaks. This is very different to other metrics such as Pearson or Spearman correlation coefficients, which tend to value dominant peaks more. Thus, the final score *s*_e,t_ is complementary to these other metrics.

### 2.3 Aligning spectra from multiple sources

Another issue arises when spectra from different spectroscopic techniques are to be aligned simultaneously (*e.g.* VCD and IR). In the current implementation of the alignment algorithm, it is assumed that for each peak in the IR spectrum there is a corresponding VCD peak in the VCD spectrum. This means that each peak in the IR spectrum has an extended list of attributes, which includes the corresponding VCD intensity. While the positions of the vibrational excited states of the system, and therefore also the wave numbers of the normal mode vibrations, are identical for IR and VCD spectroscopy, there is no correlation between the IR and VCD intensities. Namely, IR absorbance is solely based on the electric dipole transition moment, whereas VCD absorbance also depends on the magnetic dipole transition moment. As a result, after broadening the line spectra, the peaks in the IR and VCD spectra do not necessarily occur at the same positions. This is similar for Raman spectroscopy, which is often complementary to IR. Thus, a different setup is required for the alignment based on several spectroscopies simultaneously.

This issue is addressed as follows: The information from both spectroscopic sources is processed in the same manner, *i.e.*, experimental and theoretical spectrum are deconvoluted by fitting a set of pseudo-Voigts to the spectrum. The strings (*i.e.* the list of all peaks) from each spectroscopic source are then concatenated and an index *p*_*i*_ is attached as an additional attribute to each peak to specify its source. Next, the frequencies are sorted, and the matching is performed as previously described. Note that the strings from each spectroscopic source are sorted already beforehand, and that the purpose of the combined sorting is solely to move each peak from each spectroscopic source at the correct place in the concatenated string. Finally, the index *p*_*i*_ is used to split the concatenated string again, resulting in the aligned spectra of the different sources separately. The calculation of the final score is modified as,11
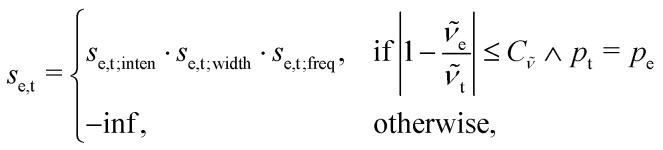
This means that if the source of the theoretical peak (*p*_t_) is not the same source as the one of the experimental peak (*p*_e_), the peaks cannot be matched (*i.e.*, *s*_e,t_ = −inf). This ensures that for instance IR peaks can only be matched to IR peaks.

## 3 Computational and experimental methods

### 3.1 Dataset

**Fig. 2:**
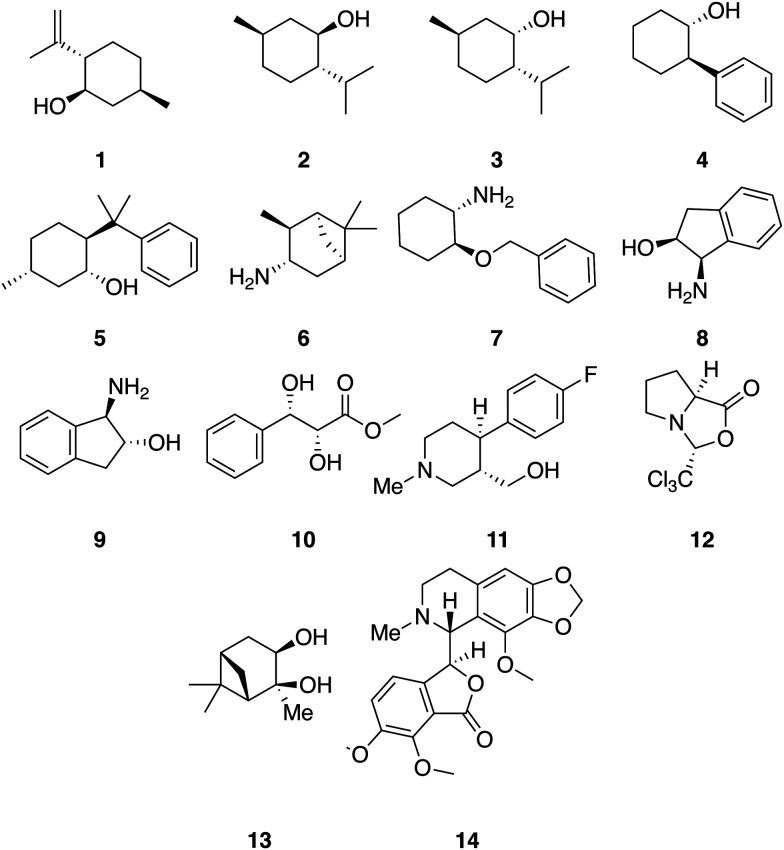
Compounds with experimental IR and Raman spectra recorded in this study: (−)-isopulegol (1), (−)-menthol (2), (+)-neomenthol (3), (1*R*,2*S*)-*trans*-2-phenyl-1-cyclohexanol (4), (+)-8-phenylmenthol (5), (+)-isopinocampheylamin (6), (1*S*,2*S*)-*trans*-2-benzyloxy-cyclohexylamin (7), (1*R*,2*S*)-(+)-*cis*-1-amino-2-indanol (8), (1*S*,2*S*)-(+)-*trans*-1-amino-2-indanol (9), methyl (2*R*,3*S*)-2,3-dihydroxy-3-phenylpropanoate (10), ((3*S*,4*R*)-4-(4-fluorophenyl)-1-methylpiperidin-3-yl)methanol (11), (3*R*,7*aS*)-3-(trichloromethyl)tetrahydro-1*H*,3*H*-pyrrolo[1,2-*c*]oxazol-1-one (12), (1*S*,2*S*,3*R*,5*S*)-2,6,6-trimethylbicyclo[3.1.1]heptane-2,3-diol (13), (−)-noscapicin (14).

**Fig. 3:**
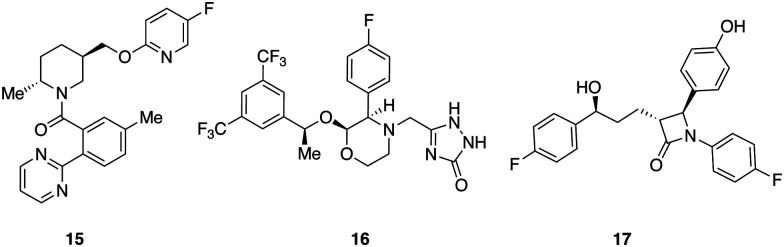
Compounds with experimental IR and VCD available from ref. 18: filorexant (15), aprepitant (16), ezetimibe (17).

A set of 14 commercially available compounds with minimum two stereocenters was selected to measure experimental IR and Raman spectra and compare with theoretical spectra (Fig. 2). In addition, three compounds, *i.e.* filorexant (15), aprepitant (16), and ezetimibe (17), were studied for which experimental IR and VCD spectra are available in ref. 18 (Fig. 3). IR and Raman spectra of 1–14 were measured experimentally either in CDCl_3_ or DMSO-*d*_6_ (see Experimental details). In Table 1, we list the solvent in the experiment for each compound, which was used in the alignment, as well as the spectral range applied.

**Table 1:** Summary of the solvent in the experiment and the spectral range used for the alignment. We focus on the range below 1500 cm^−1^ because the vibrations in this range are delocalized and probe the stereochemical environment, whereas the range above 1500 cm^−1^ does not provide useful information for determining the stereochemistry

Compound	IR	Raman	VCD	Range used [cm^−1^]
1	CDCl_3_	CDCl_3_	—	1000–1500
2	CDCl_3_	CDCl_3_	—	1000–1500
3	CDCl_3_	CDCl_3_	—	1000–1500
4	CDCl_3_	CDCl_3_	—	1000–1500
5	CDCl_3_	CDCl_3_	—	1000–1500
6	CDCl_3_	CDCl_3_	—	1000–1500
7	CDCl_3_	CDCl_3_	—	1000–1500
8	DMSO-*d*_6_	DMSO-*d*_6_	—	1150–1500
9	DMSO-*d*_6_	DMSO-*d*_6_	—	1150–1500
10	CDCl_3_	CDCl_3_	—	1000–1500
11	CDCl_3_	CDCl_3_	—	1000–1500
12	CDCl_3_	CDCl_3_	—	1000–1500
13	CDCl_3_	CDCl_3_	—	1000–1500
14	DMSO-*d*_6_	DMSO-*d*_6_	—	1150–1500
15	CDCl_3_	—	CDCl_3_	1000–1500
16	DMSO-*d*_6_	—	DMSO-*d*_6_	1000–1500
17	DMSO-*d*_6_	—	DMSO-*d*_6_	1150–1500

### 3.2 Computational details

**Fig. 4:**
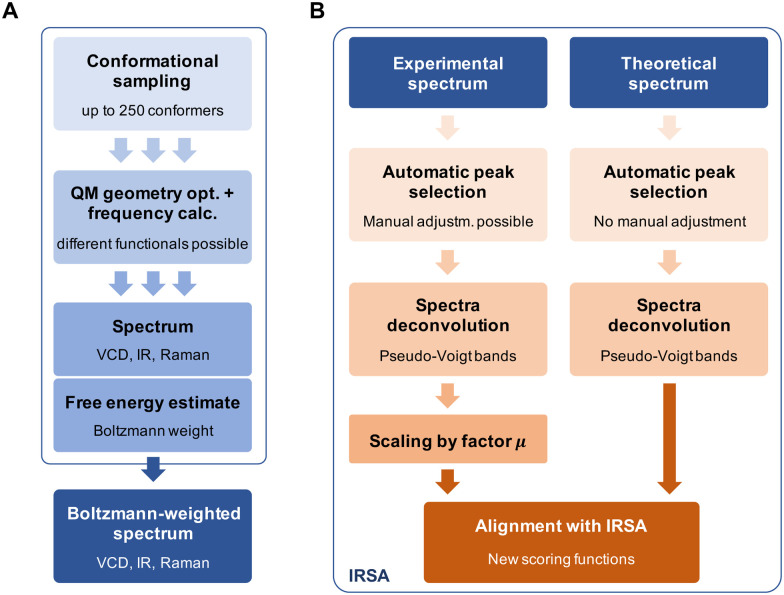
(A) Calculation of the Boltzmann-weighted theoretical spectrum involving conformational sampling, geometry optimization and frequency calculation. (B) Post-processing of the theoretical and experimental spectra, followed by the alignment with IRSA.

Fig. 4 gives a schematic overview of the calculation of the theoretical spectra and the alignment process. First, a theoretical spectrum is generated using conformational sampling, QM geometry optimization and frequency calculation. When calculating a spectrum for a conformational ensemble instead of a single conformation, the contributions of the conformers need to be weighted according to their relative (free) energies, *i.e.*, Boltzmann weights (see eqn (A1) in the Appendix). With the theoretical and experimental spectra at hand, the spectra are post-processed and aligned with the IRSA algorithm. The computational details for the conformational sampling, QM calculations, and the post-processing and alignment of the spectra are given in the Appendix.

#### 3.2.1 Analysis

The aligned theoretical spectra as well as the experimental spectrum were interpolated by cubic splines in the range between 1000 cm^−1^ and 1500 cm^−1^ in steps of 1 cm^−1^. Pearson correlation coefficient *r*_P_ and Spearman correlation coefficient *r*_S_ were computed between the interpolated experimental spectrum and the interpolated theoretical spectra. The score *s* is obtained from the IRSA algorithm.

The different quantitative metrics encode different properties of the superimposed spectra: the Pearson coefficient is an overlap metric, the Spearman coefficient contains information about the derivatives of the spectrum, whereas the alignment score *s* directly informs about the “work” performed by the alignment algorithm. To facilitate comparison, we have introduced in ref. 14 a combined score, which is obtained by multiplying the total alignment score and the Pearson correlation coefficient: *s*_P_ = *s*·*r*_P_. Here, we extend the metric by using also the Spearman correlation, since we found that it provides valuable additional information. Further, we include here the metrics from the Raman spectrum, thus *s*_comb_ = *s*·*r*^IR^_P_·*r*^IR^_S_·*r*^Raman^_P_·*r*^Raman^_S_. For IR and VCD, we have similarly *s*_comb_ = *s*·*r*^IR^_P_·*r*^IR^_S_·*r*^VCD^_P_·*r*^VCD^_S_.

Note that the score *s*_comb_ for an isomer can be any value, and its absolute meaning has no significance. However, the best performing isomer should have the highest score *s*_comb_. If two isomers are to be distinguished but both values are very similar, additional theoretical and/or experimental information needs to be included to increase the confidence in the assignment. The Pearson and Spearman correlation coefficients can be used for additional diagnostic purposes. For example, a low Pearson correlation coefficient *r*_P_ is an indication that the computational setup is in poor agreement with experiment.

### 3.3 Experimental details

#### 3.3.1 Materials and sample preparation

Compounds 1–14 were purchased from Sigma-Aldrich, Germany, and were used without further purification. The solvent was either CDCl_3_ or DMSO-*d*_6_ (see Table 1), depending on their solubility. The concentrations ranged from 25 to 130 mg mL^−1^ and were adapted for each compound so that an optimal IR absorbance range was reached. The same concentrations were used to perform the Raman measurements.

#### 3.3.2 IR spectroscopy

The IR absorbance spectra were recorded using the Bruker Invenio FT-IR spectrometer at room temperature. The resolution was set to 1 cm^−1^. A 200 μm path-length cell was used equipped with BaF_2_ windows. In total, 128 scans were recorded for each compound, corresponding to a measurement time of 2 min. The IR spectra were solvent corrected.

#### 3.3.3 Raman spectroscopy

The Raman spectra were recorded on a home-built spectrometer with resolution 7 cm^−1^. The excitation wavelength used was 532 nm. The laser power at the source ranged from 500 to 700 mW and was adapted for each compound to reach desired CCD detector intensity read-outs. The total acquisition time was 10 min. Solvent spectra were recorded accordingly to perform a solvent subtraction. Finally, a baseline correction was done using the procedure as described by Boelens *et al.*^31^

### 3.4 Data and software availability

The code to reproduce the calculations and alignments is available as a Jupyter notebook on the Github repository (https://www.github.com/rinikerlab/irsa, release https://github.com/rinikerlab/irsa/releases/tag/Boeselt_PCCP_2022 and Zenodo DOI url https://doi.org/10.5281/zenodo.7428760). The experimental and computed data for compounds 1–14 is published on the ETH Research collection (https://doi.org/10.3929/ethz-b-000586421).

## 4 Results and discussion

### 4.1 Combination of IR and Raman spectroscopy

**Fig. 5:**
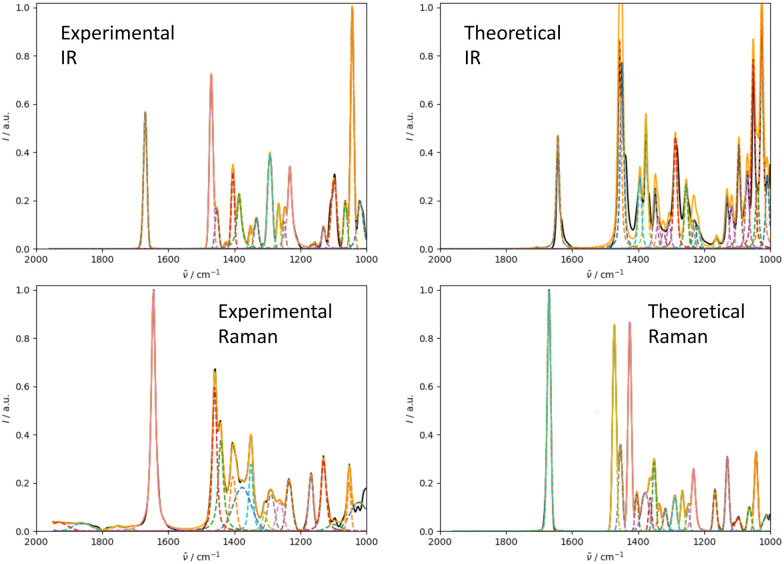
Deconvolution of the experimental (left) and theoretical (right) IR (top) and Raman (bottom) spectrum of isopulegol (1) by fitting pseudo-Voigt bands (colored dashed lines). The black line represents the original experimental spectrum (behind the colored lines), the red line the original theoretical spectrum of isomer 0. The continuous orange line shows the spectrum, which results from superimposing all pseudo-Voigt bands (colored dashed lines) with each other.

We illustrate the workflow for combining IR and Raman spectra for stereochemical assignment using the example of isopulegol (1). The unaligned, aligned, and experimental spectra for compounds 2–14 are provided in the ESI.† The conformational search with OMEGA identified 17 different conformers for 1. The theoretical Raman and IR spectra were generated by convoluting the obtained frequencies and IR/Raman intensities with a Lorentzian bandwidth of 12 cm^−1^. The final theoretical Raman and IR spectra for the conformational ensemble were obtained by Boltzmann-weighting the theoretical spectra assuming a temperature of 298.15 K. Next, the theoretical and experimental spectra were deconvoluted assuming a set of pseudo-Voigt bandshapes (Fig. 5). All peaks were automatically selected using the peak detection algorithm as implemented in scipy.^32^ For complicated cases (*e.g.* noisy spectra), a manual selection might be necessary.

The converged parameters for the pseudo-Voigt bandshapes (*η*, *I*, *w* and *x*_0_) were extracted from the fit and subsequently used in the alignment with the IRSA algorithm.

**Fig. 6:**
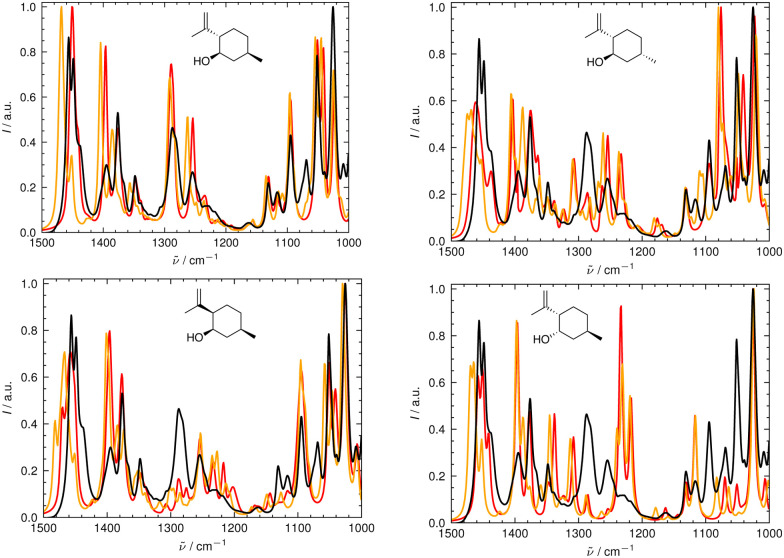
Superimposed experimental (black), aligned (red), and scaled-but-unaligned (orange, *μ* = 0.980) theoretical IR spectra of isopulegol (1). The aligned results (red) used *μ* = 0.975. The alignment was assessed using the combined score *s*_comb_ from the IR and Raman spectra. (Top left): isomer 0 (correct isomer). (Top right): isomer 1. (Bottom left): isomer 2. (Bottom right): isomer 3.

**Fig. 7:**
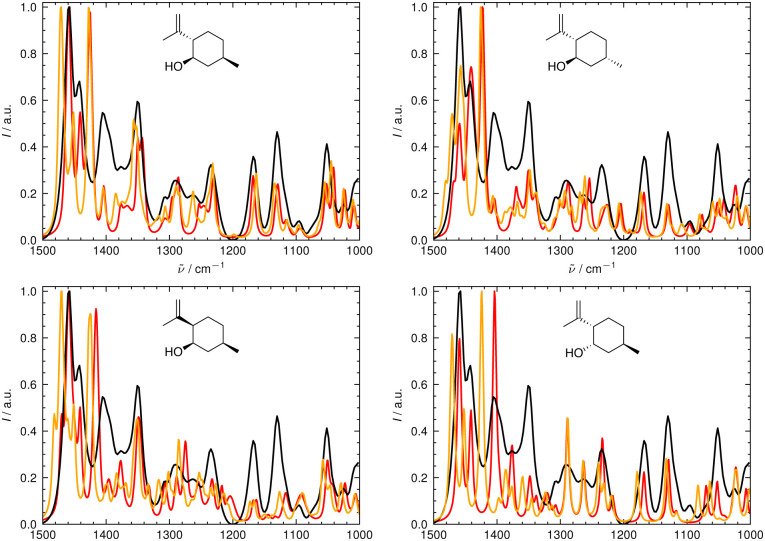
Superimposed experimental (black), aligned (red), and scaled-but-unaligned (orange, *μ* = 0.980) theoretical Raman spectra of isopulegol (1). The aligned results (red) used *μ* = 0.975. The alignment was assessed using the combined score *s*_comb_ from the IR and Raman spectra. (Top left) isomer 0 (correct isomer). (Top right) isomer 1. (Bottom left) isomer 2. (Bottom right) isomer 3.

In Fig. 6 and 7, the aligned theoretical and experimental IR and Raman spectra are shown for the correct isomer (top left panel) and for the other isomers of isopulegol (**1**). The orange line represents the theoretical spectrum scaled solely by the constant factor *μ* = 0.98 (*i.e.* no alignment algorithm applied).

Only the range between 1000 cm^−1^ and 1500 cm^−1^ was used for the alignment with the IRSA algorithm because the vibrations between 1500 cm^−1^ and 2000 cm^−1^ typically belong to localized OH and NH_2_ vibrations, which are very dominant but do not probe the stereochemical information. When the spectral region below 1000 cm^−1^ is resolved in the experimental spectrum (*e.g.*, with attenuation total reflection (ATR) IR), it is worth including this range in the analysis.

It can already be seen visually that the theoretical spectra of isomer 0 (the correct isomer) fit best to the experimental ones (top left panel in Fig. 6 and 7). The other isomers (remaining panels) agree less well with experiment. The quality of the alignments of the IR and Raman spectra is assessed quantitatively by calculating the different metrics: the total alignment score *s*, the Pearson correlation coefficient *r*_P_ and the Spearman correlation coefficient *r*_S_ as well as the combined score *s*_comb_ (Table 2). The value of *s*_comb_ is highest for the correct isomer 0.

**Table 2:** Combined score *s*_comb_ computed for the aligned IR and Raman spectra of all isomers of compounds 1–14. The number in parentheses indicates the scaling factor *μ*, for which the best metric was obtained. The last column gives the ratio between *s*_comb_ of the correct isomer (isomer 0) and *s*_comb_ of the second best isomer

Compound	Isomer 0	Isomer 1	Isomer 2	Isomer 3	Ratio
1	0.35 (0.975)	0.14 (0.970)	0.17 (0.975)	0.09 (0.975)	2.5
2	0.47 (0.975)	0.07 (0.975)	0.11 (0.970)	0.21 (0.975)	2.2
3	0.34 (0.975)	0.20 (0.970)	0.15 (0.970)	0.15 (0.975)	1.7
4	0.35 (0.980)	0.09 (0.975)	—	—	3.9
5	0.16 (0.975)	0.08 (0.975)	0.09 (0.975)	0.07 (0.980)	2.0
6	0.18 (0.970)	0.13 (0.980)	0.11 (0.975)	0.11 (0.975)	1.4
7	0.37 (0.975)	0.22 (0.975)	—	—	1.7
8	0.03 (0.970)	0.003 (0.97)	—	—	7.4
9	0.013 (0.970)	0.008 (0.975)	—	—	1.6
10	0.09 (0.980)	0.06 (0.985)	—	—	1.5
11	0.34 (0.975)	0.24 (0.975)	—	—	1.4
12	0.15 (0.980)	0.06 (0.975)	—	—	2.5
13	0.24 (0.975)	0.15 (0.970)	0.15 (0.975)	0.08 (0.975)	1.6
14	1.00 (0.985)	0.25 (0.975)	—	—	4.0

**Fig. 8:**
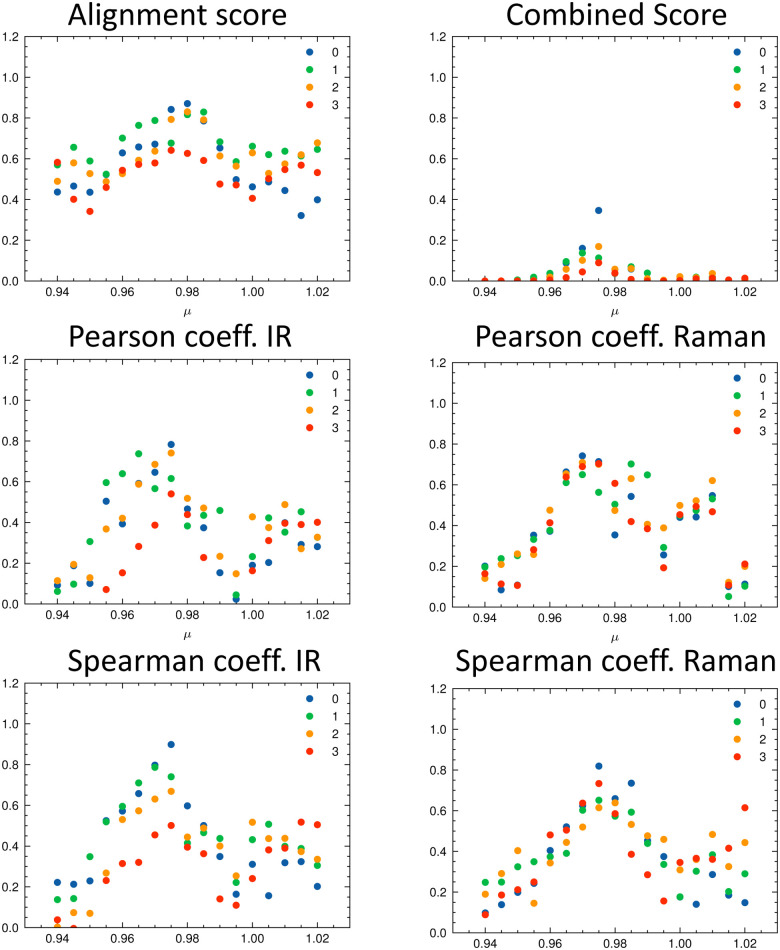
Evaluation metrics as a function of the scaling factor *μ* for the four isomers of isopulegol (1): total alignment score *s* (top left), combined score *s*_comb_ (top right), Pearson correlation coefficient (middle panels) and Spearman correlation coefficient (bottom panels) for IR and Raman. The best match in terms of the combined score *s*_comb_ is obtained with *μ* = 0.975 for isomer 0 (correct isomer).

As discussed in the Appendix Section A1.2, using an universal scaling factor fails in practice. Therefore, a screening around the tabulated value is often performed to determine *μ* for a given compound.^33^ We followed this approach and varied *μ* in the range of [0.94…1.02] in steps of 0.005. For each value of *μ*, we re-calculated the metrics and determined which isomer matches the experiment best (Fig. 8). In Fig. 8, the importance of the alignment score *s* becomes eminent: *s* is higher for the correct isomer (isomer 0, blue dots, top left panel) than for the other three isomers. The overlap metrics (Pearson and Spearman correlation coefficients) are not robust for the correct isomer across the range of *μ*. By combining the different metrics in *s*_comb_, the certainty of the assignment can be increased due to the complementary information. The best match was found for the correct isomer 0 with *μ* = 0.975. We noticed that the plot of the combined score *s*_comb_ as a function of *μ* resembles a normal distribution for each isomer. For the chosen level of theory, this maximum lies for compounds 1–14 on average at *μ* = 0.975 (see Table 2).

Table 2 gives the numerical results for the complete set of compounds 1–14. The aligned spectra, and the individual values for the different metrics are provided in the ESI.† The combined score *s*_comb_ is highest for the correct isomer for all compounds. Cases where *s*_comb_ is very similar for more than one isomer might need further validation using additional experiments. We note that the absolute value of *s*_comb_ can be very small due to its definition. Thus, we also compare the ratio between *s*_comb_ of the best performing isomer (isomer 0) and the second best isomer (see last column in Table 2). If the ratio is close to one, then the theoretical spectra of the two best matching isomers describe the experimental spectra similarly well.

### 4.2 Combining IR and VCD spectroscopy

**Fig. 9:**
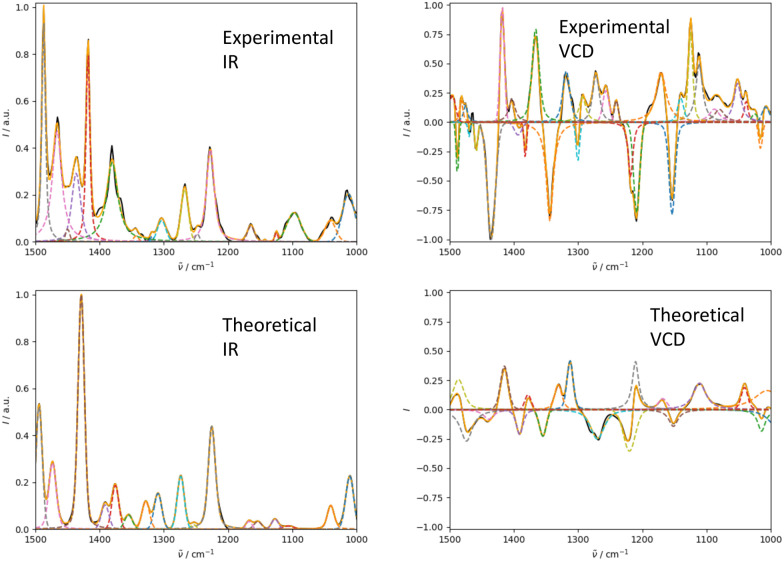
Deconvolution of the experimental (top) and theoretical (bottom) IR (left) and VCD (right) spectra of filorexant (15) by fitting pseudo-Voigt bands (colored dashed lines). The black line represents the original experimental spectrum (behind the colored lines), the red line the original theoretical spectrum for isomer 0. The continuous orange line shows the spectrum, which results from superimposing all pseudo-Voigt bands (colored dashed lines) with each other.

We illustrate the workflow for combining IR and VCD spectra for stereochemical assignment using the example of filorexant (15). The unaligned, aligned, and experimental spectra for compounds aprepitant (16) and ezetimibe (17) are provided in the ESI.† In Fig. 9, the experimental and theoretical IR and VCD spectra are displayed for the correct isomer, with deconvoluted pseudo-Voigt bands shown as dashed lines.

**Fig. 10:**
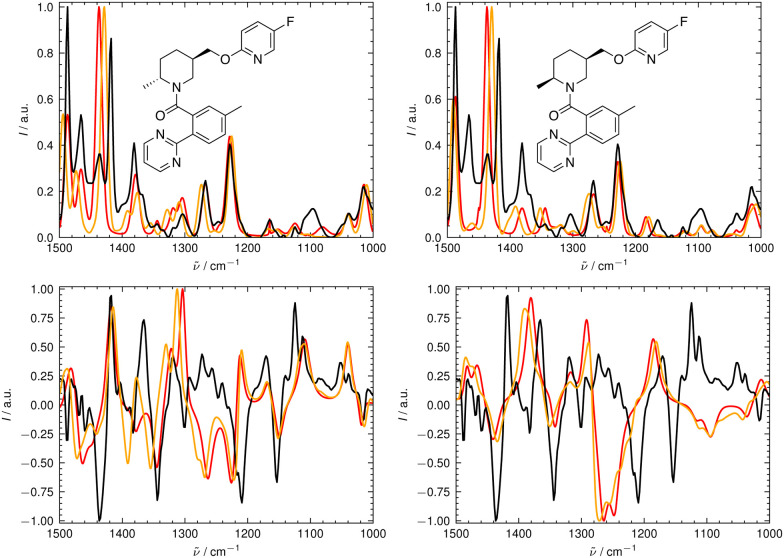
Superimposed experimental (black), aligned (red), and scaled-but-unaligned (orange, *μ* = 0.98) theoretical IR (top) and VCD (bottom) spectra of filorexant (15). The aligned spectra (ref) used *μ* = 0.98. The alignment was assessed using the combined score *s*_comb_ from the IR and VCD spectra. (Left): isomer 0 (correct isomer). (Right): isomer 1.

**Fig. 11:**
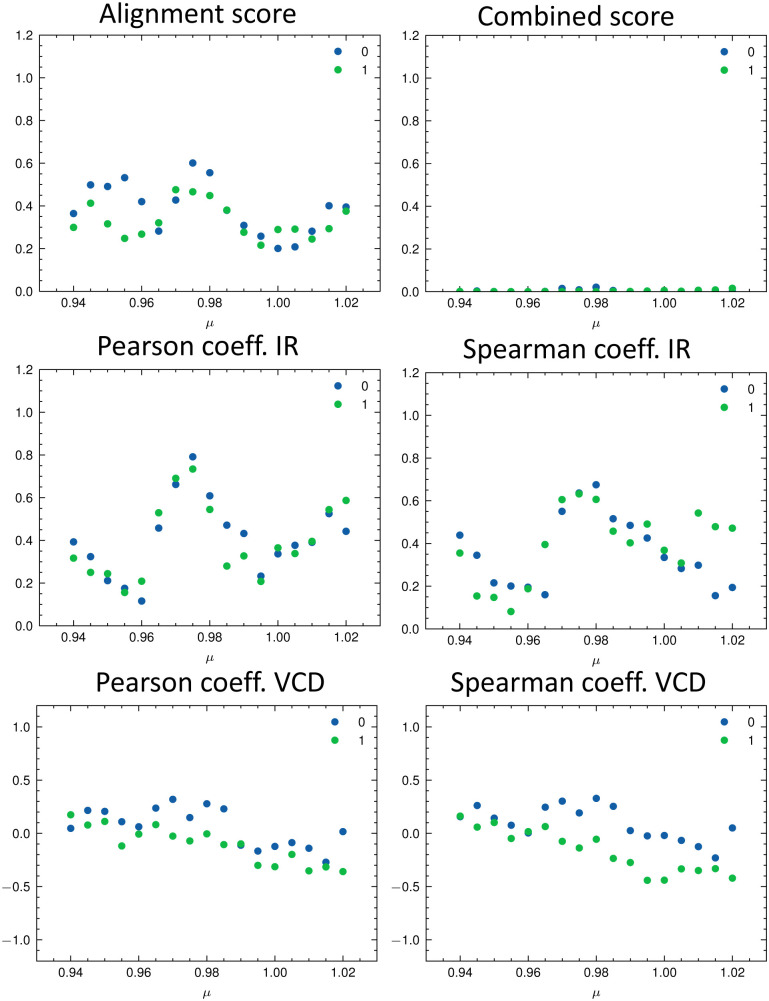
Evaluation metrics as a function of the scaling factor *μ* for the two isomers of filorexant (15): total alignment score *s* (top left), combined score *s*_comb_ (top right), Pearson correlation coefficient (middle panels), and Spearman correlation coefficient (bottom panels) for the IR (left) and VCD (right) spectra. The best match in terms of the combined score *s*_comb_ is obtained with *μ* = 0.98 for isomer 0 (correct isomer).

The parameters extracted from the fitted pseudo-Voigt bands were subsequently used in the IRSA algorithm. The results are shown in Fig. 10. Again, the correct isomer 0 (left panels in Fig. 10) agrees better with the experimental data than the incorrect isomer 1. The evaluation metrics as a function of the scaling factor *μ* are shown in Fig. 11. The highest combined score *s*_comb_ is obtained for isomer 0, the correct isomer. Note that with VCD spectra also the correct absolute stereochemistry can be extracted. As the scoring function is insensitive towards the sign in the VCD spectrum, the alignment is performed exactly the same for the enantiomer. Thus, the resulting overlap metrics for the VCD spectra are anti-correlated for the incorrect enantiomer. A positive sign in the Pearson and Spearman correlation coefficients of the VCD spectrum indicates that the correct enantiomer is computed with this setup.

Table 3 gives the numerical results for compounds 15–17. The aligned spectra, and the individual values for the different metrics are listed in the ESI.† The combined score *s*_comb_ is highest for the correct isomer for all compounds. We would like to emphasize that the combination of IR and VCD spectroscopy allows for a clear distinction between the isomers as illustrated by the well separated scores in Table 3 as well as by visual comparison of the aligned spectra (see ESI†).

**Table 3:** Combined score *s*_comb_ computed for the aligned IR and VCD spectra of all isomers of compounds 15–17. The score is highest for the correct isomer for all compounds. The numbers in the parentheses indicate the scaling factor used to obtain the highest score

Compound	Isomer 0	Isomer 1	Isomer 2	Isomer 3	Ratio
15	0.02 (0.980)	0.002 (0.975)	—	—	10.0
16	0.21 (0.980)	0.005 (0.980)	0.07 (0.975)	0.15 (0.985)	1.4
17	0.04 (0.985)	0.007 (0.975)	0.003 (0.970)	0.02 (0.980)	1.6

### 4.3 General discussion

While the modifications of the IRSA algorithm presented in this study improve its performance and robustness, some sources of errors remain, which we discuss below together with suggestions on how to address them.

#### 4.3.1 Errors in the free-energy landscape

The quality of the theoretical spectra depends heavily on the exhaustiveness and accuracy of the conformational sampling. In this study, we used the OMEGA conformer generator with custom settings. Conformer generators typically use torsion libraries and/or other heuristics for torsional preferences. In some cases, such heuristics may cause relevant conformations to be missed. Cross-checking with different conformer generators or settings (*e.g.* disabling the use of heuristics), or using alternative methods such as molecular dynamics may help to assess the quality of the conformational sampling.

Another source of error is the estimation of the free-energy landscape. Errors of DFT seen in benchmarking studies (see *e.g.* ref. 34) are typically large enough that the global minimum structure can be shifted. Neglected solute-solute and/or solvent-solute interactions can increase this error. An interesting solution is demonstrated by the program DP4+.^35^ DP4+ compares computed NMR spectra with experimental ones. The authors of DP4+ realized that the computed free energies (and thus the weights of the conformers in the ensemble) are of crucial importance for a correct assignment. They proposed to alter the free-energy landscape repeatedly by perturbing it with randomly drawn floating numbers, followed by a statistical analysis. It is straight forward to combine such an approach with the IRSA algorithm.

It is worth mentioning that a statistical analysis of the metrics as a function of the perturbed free energies can give insight into the experimentally measured conformational ensemble. The randomly perturbed free-energy landscape, which matches the experimental spectrum best, is likely to resemble the measured free-energy landscape. We pursued a similar idea already in ref. 9.

#### 4.3.2 Errors in the computed frequency spectra

The neglect of anharmonic effects and solvent effects can alter the theoretical spectrum. One advantage of the IRSA algorithm is that it can account for these effects partially. However, if the effects are too strong, a comparison with experiment becomes unfeasible. The problem can only be resolved if one explicitly considers anharmonic effects, *e.g.*, by employing first-principles molecular dynamics simulations,^36,37^ and/or includes the solvent.^38,39^ For the molecules studied in this work, these measures were not necessary as the effects were small.

#### 4.3.3 Band-shape analysis and peak selection

The computed vibrational spectra has to be broadened to make the results comparable with experiment. The IRSA algorithm performs an automated band-shape analysis (deconvolution) of the theoretical and experimental spectra, and uses the information extracted to perform the alignment. If the band-shape analysis of the experimental spectrum leads to vastly different results than the analysis of the theoretical spectrum, the algorithm will encounter difficulties to align the spectra. We expect that improving the broadening scheme and the band-shape analysis will greatly enhance the performance of the algorithm, and increase the significance of the alignment score *s* further.

Peaks that are fully overlapping in the experimental spectrum do not necessarily have to be fused in the computed spectrum (or the other way around). In these cases, the peak selection might identify only one peak in the experimental spectrum, while two peaks are identified in the theoretical spectrum (or *vice versa*). While the algorithm can still align the peaks, the score *s* is perturbed. In future work, we will explore whether this issue can be addressed by not fixing the Lorentzian band shape of the theoretical spectrum at the beginning, but rather compute an individual bandwidth for each peak.

## 5 Conclusions

In this study, we presented an updated version of the IRSA algorithm. The changes introduced are the following: (i) the algorithm handles overlapping peaks *via* deconvolution of the spectra. Thus, the bandwidth can now be used in the alignment algorithm. (ii) The scoring function was adopted further such that it converges smoothly to zero, which limits how far peaks can be shifted. (iii) The algorithm can perform multiple sequence alignment with spectra from different sources (*e.g.* IR and Raman or VCD).

We demonstrated the performance of the algorithm on a set of 14 compounds, for which we measured IR and Raman spectra, as well as a set of three compounds, for which experimental IR and VCD spectra were available in the literature. Especially the combination IR and VCD allows for the determination of the correct stereoisomer with high accuracy as the VCD spectra show larger differences among isomers. The quality of the alignment can be assessed with different quantitative metrics, which carry different information. Here, we used the alignment score and a set of overlap metrics (*i.e.* the Pearson and Spearman correlation coefficients). Note that also other metrics used in spectroscopy could be integrated. The total alignment score informs about the “work” needed to align the spectra, whereas the Pearson and Spearman correlation coefficients assess the overlap of the aligned spectra. The different information can be further combined into a score *s*_comb_ for quantitative comparison between isomers. When plotting *s*_comb_ as a function of the scaling factor *μ*, the resulting curve resembles a normal distribution with a maximum around *μ* = 0.975 for the B3LYP(G)/def2-TZVP level of theory. In cases where the algorithm is uncertain, additional experimental data should be included and/or the conformational ensemble and the computational methods adapted.

Considering the performance of the updated IRSA algorithm presented here, we believe that it is at the forefront of quantitative-based spectral assignments and ready to be used in spectral case studies (both in academia and industry).

## Conflicts of interest

There are no conflicts to declare.

## Supplementary Material

CP-025-D2CP04907D-s001

## Appendices

### Appendices

#### A1.1 Sampling of Conformations and Quantum-Mechanical Calculations

A set of conformer geometries was generated for compounds 1–17 using the exhaustive torsion sampling method as implemented in OMEGA.^40^ The number of possible conformations was set to 4500, although the search can already be exhausted at a lower number of conformers. The maximum energy cutoff was set to 15 kcal mol^−1^ for compounds 1–14 and 10 kcal mol^−1^ for compounds 15–17, with sampling of the hydrogen rotations explicitly enabled. The conformers of compounds 1–14 were optimized on the RIJCOSX^41,42^-B3LYP^43^(G)^44^-D4^45,46^/def2-TZVP^47^ level of theory (no implicit solvent), followed by frequency calculations to obtain a free-energy estimate using the ORCA/5^48^ software package. The calculations made use of the auxilary basis set def2/J.^49^ Raman spectra were obtained by displacing the normal coordinates numerically and computing the polarizability. The (G) refers to the B3LYP definition as in Gaussian.^44^ Other options of the calculations were tight convergence criteria in the self-consistent field methodology, an integration grid 6 (Grid6), and tight optimization criteria. Compounds 15–17 were optimized on the B3LYP(G)-D3/6G++31(d,p)^50^ level of theory using the Gaussian09^44^ software package. The main reason for the different computational workflow is that ORCA does currently not support VCD calculations, and the Poble basis set is the standard basis set used in Gaussian. The integration grid was set to ultrafine, and the convergence criterium of the SCF procedure as well as that of the geometry optimization were set to tight. The geometry optimization was followed by frequency calculations and VCD^51–53^ calculations.

#### A1.2 Post-processing and Alignment of the Spectra

The resulting theoretical vibrational spectra were checked for imaginary frequencies and Boltzmann-weighted according to

A1

and Lorentzian broadened with

A2

where *I* is the IR, VCD, or Raman intensity, and *w* is a tunable parameter set to 12 cm^−1^ for all cases.

Next, the experimental and theoretical spectra were deconvoluted by fitting a set of pseudo-Voigt bands^29^ as described in Section 2.1. For IR and Raman spectra, peaks were automatically assigned using the peak finding algorithm in scipy^32^ with standard parameters. For VCD spectra, we implemented a simple threshold criterium: If the absolute value of the current position is greater than the absolute value of the next and the previous position, the position is marked as a peak. VCD peaks below an intensity of 0.03 after normalization were discarded. For each peak, a pseudo-Voigt band was placed with an initial amplitude equal to the peak height *I*, an initial center position *x*_0_ equal to the peak position, an initial bandwidth of *w* = 12 cm^−1^, and an initial mixing parameter *η* = 1.0. This resulted in a set of parameters equal to the number of peaks times four, *i.e.*, {*η*_0_,*η*_1_,…,*x*_0,1_,*x*_0,2_,…,*I*_0_,*I*_1_,…,*w*_0_,*w*_1_,…}. The parameters were then fitted to reproduce the spectra as accurately as possible. For this, the parameters were varied within a specified range. The range for the intensities *I* was set to [0, 1] (for VCD: [−1, +1]), the range for the bandwidth *w* to [1,64] cm^−1^, and the range of the peak position to the selected peak position *x*_0_ ± 2 cm^−1^. The fitting was performed using a least square method as implemented in the lmfit library.^54^

The theoretical spectra were first scaled by *μ* and subsequently, the fitted parameters were used in the alignment with the IRSA algorithm. The value of *μ* was varied for each isomer to maximize the quantitative metrics. The exact value is specified for each case. Screening of a range of values for *μ* can be justified as follows. *μ* is typically extracted for a specific level of theory from sources such as ref. 16 for small compounds. This corrects for the error, which is introduced in the calculation by assuming a set of harmonic oscillators. It is often found that a universal scaling factor performs poorly, especially for flexible compounds.^18^ To address this, a common solution is to determine a specific scaling factor by maximizing a chosen metric.^33^

The cutoff values for the scoring functions in eqn (5)–(7) used in this study are summarized in Table 4. A cutoff parameter of 0.01 means that the respective attribute is allowed to differ by 1% in order to be matched (*e.g.*, for *μ* = 0.98, the search range for suitable experimental peaks is 0.97–0.99). We tested this set of cutoff parameters on compound 1, and found a satisfying performance of the algorithm.

**Table 4:** Cutoff parameters used for the scoring functions in eqn (5)–(7). The values are fraction dependent, *e.g.*, 1.0 means that the values may differ by 100%, 0.01 means that the values may differ by 1%

	Cutoff [a.u.]	Description
*C*_*ν̃*_	0.01	Cutoff value for frequencies
*C*_*I*_	1.00	Cutoff value for intensities
*C*_*w*_	8.00	Cutoff value for bandwidths
