# Supplementary material for: Improving the IR spectra alignment algorithm with spectra deconvolution and combination with Raman or VCD spectroscopy
Source: Phys Chem Chem Phys. 2022 Dec 22;25(3):2063–74. doi: 10.1039/d2cp04907d (PMC9847344; doi:10.1039/d2cp04907d)
Supplement: CP-025-D2CP04907D-s001 [file CP-025-D2CP04907D-s001.pdf]

Electronic Supplementary Material (ESI) for Physical Chemistry Chemical Physics.
This journal is © the Owner Societies 2022

SUPPORTING INFORMATION
Improving the IR Spectra Alignment Algorithm with Spectra Deconvolution and Combination with Raman or VCD Spectroscopy

Lennard Böselt,[a] Roy Aerts,[b] Wouter Herrebout,[b] Sereina Riniker*[a]

[a] *Laboratory of Physical Chemistry, ETH Zurich, Vladimir-Prelog-Weg 2, 8093 Zurich, Switzerland*
Email: sriniker@ethz.ch
[b] *Department of Chemistry, University of Antwerp, Antwerp, Belgium*

Contents

1 Isomer Definition **S4**

2 Correlation Plots **S7**
2.1 Compound 2 . . . . S7
2.1.1 Alignment Alignment Score s and Combined Score S7
2.1.2 Pearson Correlation Coefficient S7
2.1.3 Spearman Correlation Coefficient S8
2.2 Compound 3 . . . . S8
2.2.1 Alignment Score s and Combined Score S8
2.2.2 Pearson Correlation Coefficient S9
2.2.3 Spearman Correlation Coefficient S9
2.3 Compound 4 . . . . S10
2.3.1 Alignment Score s and Combined Score S10
2.3.2 Pearson Correlation Coefficient S10
2.3.3 Spearman Correlation Coefficient S11
2.4 Compound 5 . . . . S11
2.4.1 Alignment Score s and Combined Score S11
2.4.2 Pearson Correlation Coefficient S12
2.4.3 Spearman Correlation Coefficient S12
2.5 Compound 6 . . . . S13
2.5.1 Alignment Score s and Combined Score S13
2.5.2 Pearson Correlation Coefficient S13
2.5.3 Spearman Correlation Coefficient S14
2.6 Compound 7 . . . . S14
2.6.1 Alignment Score s and Combined Score S14
2.6.2 Pearson Correlation Coefficient S15
2.6.3 Spearman Correlation Coefficient S15

2.7 Compound 8 . . . . . S16
2.7.1 Alignment Score s and Combined Score S16
2.7.2 Pearson Correlation Coefficient S16
2.7.3 Spearman Correlation Coefficient S17
2.8 Compound 9 . . . . . S17
2.8.1 Alignment Score s and Combined Score S17
2.8.2 Pearson Correlation Coefficient S18
2.8.3 Spearman Correlation Coefficient S18
2.9 Compound 10 . . . . . S19
2.9.1 Alignment Score s and Combined Score S19
2.9.2 Pearson Correlation Coefficient S19
2.9.3 Spearman Correlation Coefficient S20
2.10 Compound 11 . . . . . S20
2.10.1 Alignment Score s and Combined Score S20
2.10.2 Pearson Correlation Coefficient S21
2.10.3 Spearman Correlation Coefficient S21
2.11 Compound 12 . . . . . S22
2.11.1 Alignment Score s and Combined Score S22
2.11.2 Pearson Correlation Coefficient S22
2.11.3 Spearman Correlation Coefficient S23
2.12 Compound 13 . . . . . S23
2.12.1 Alignment Score s and Combined Score S23
2.12.2 Pearson Correlation Coefficient S24
2.12.3 Spearman Correlation Coefficient S24
2.13 Compound 14 . . . . . S25
2.13.1 Alignment Score s and Combined Score S25
2.13.2 Pearson Correlation Coefficient S25
2.13.3 Spearman Correlation Coefficient S26
2.14 VCD spectra S26

3 Aligned Spectra **S28**
3.1 Compound 1 . . . . . S28
3.2 Compound 2 . . . . . S29
3.3 Compound 3 . . . . . S31
3.4 Compound 4 . . . . . S32
3.5 Compound 5 . . . . . S33
3.6 Compound 6 . . . . . S34
3.7 Compound 7 . . . . . S35
3.8 Compound 8 . . . . . S36
3.9 Compound 9 . . . . . S37
3.10 Compound 10 . . . . . S38
3.11 Compound 11 . . . . . S39
3.12 Compound 12 . . . . . S40
3.13 Compound 13 . . . . . S41
3.14 Compound 14 . . . . . S42
3.15 Compound 15 . . . . . S43

3.16 Compound 16 . . . . S44
3.17 Compound 17 . . . . S45

1 Isomer Definition

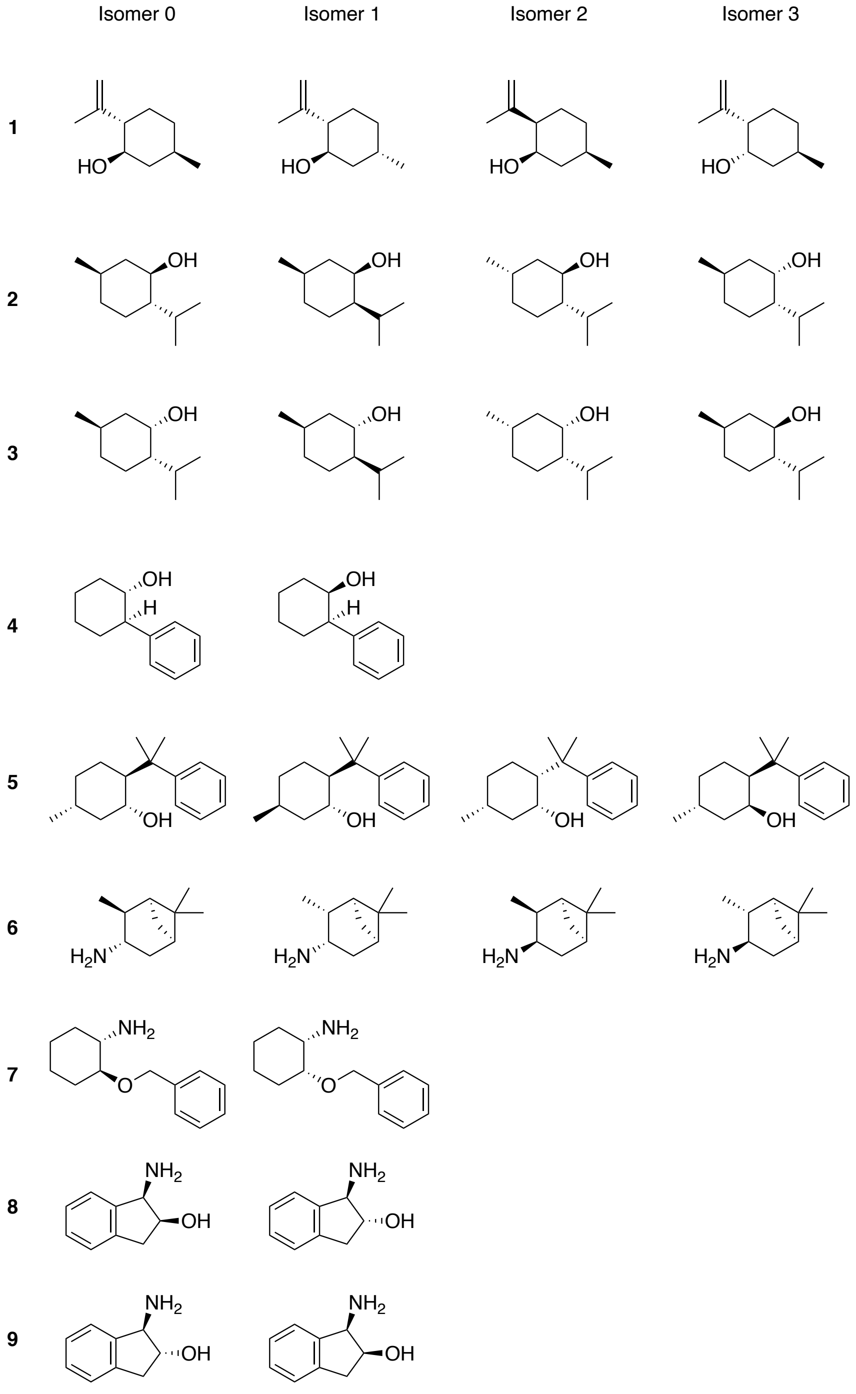

Figure S1: Isomer definition used for compounds **1**-**9**.

Isomer 0 | Isomer 1

10, 11, 12, 13, 14

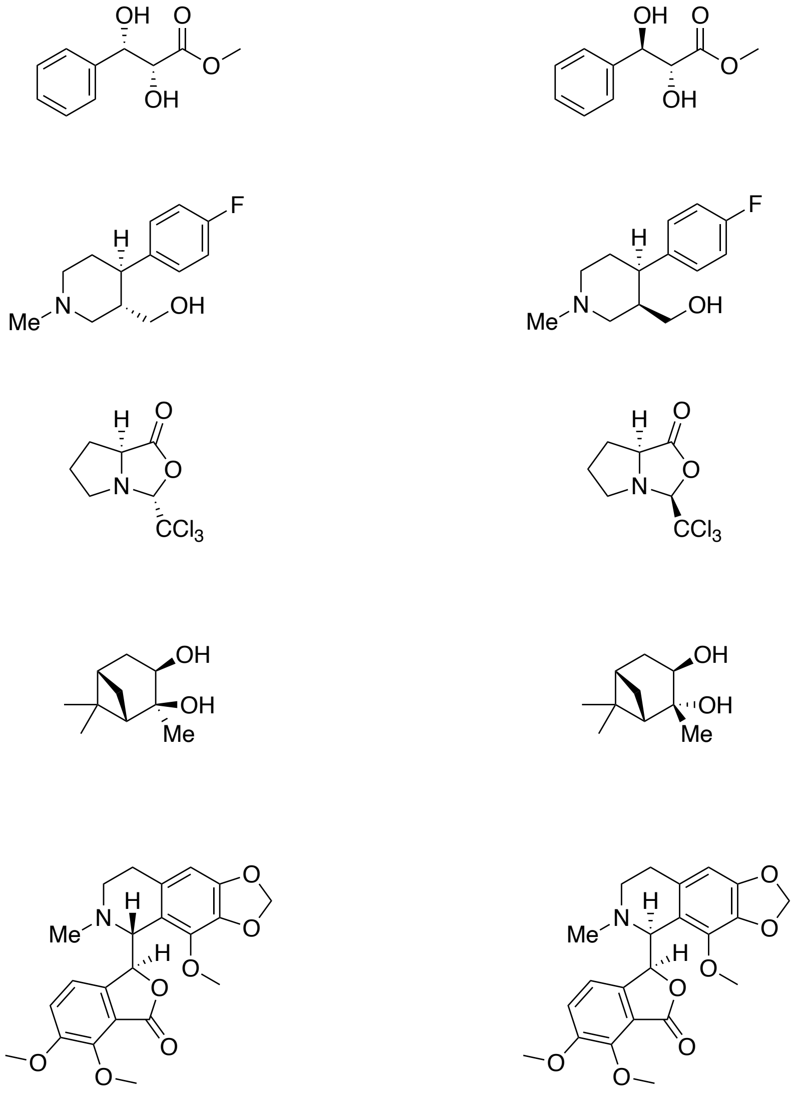

Figure S2: Isomer definition used for compounds **10**-**14**.

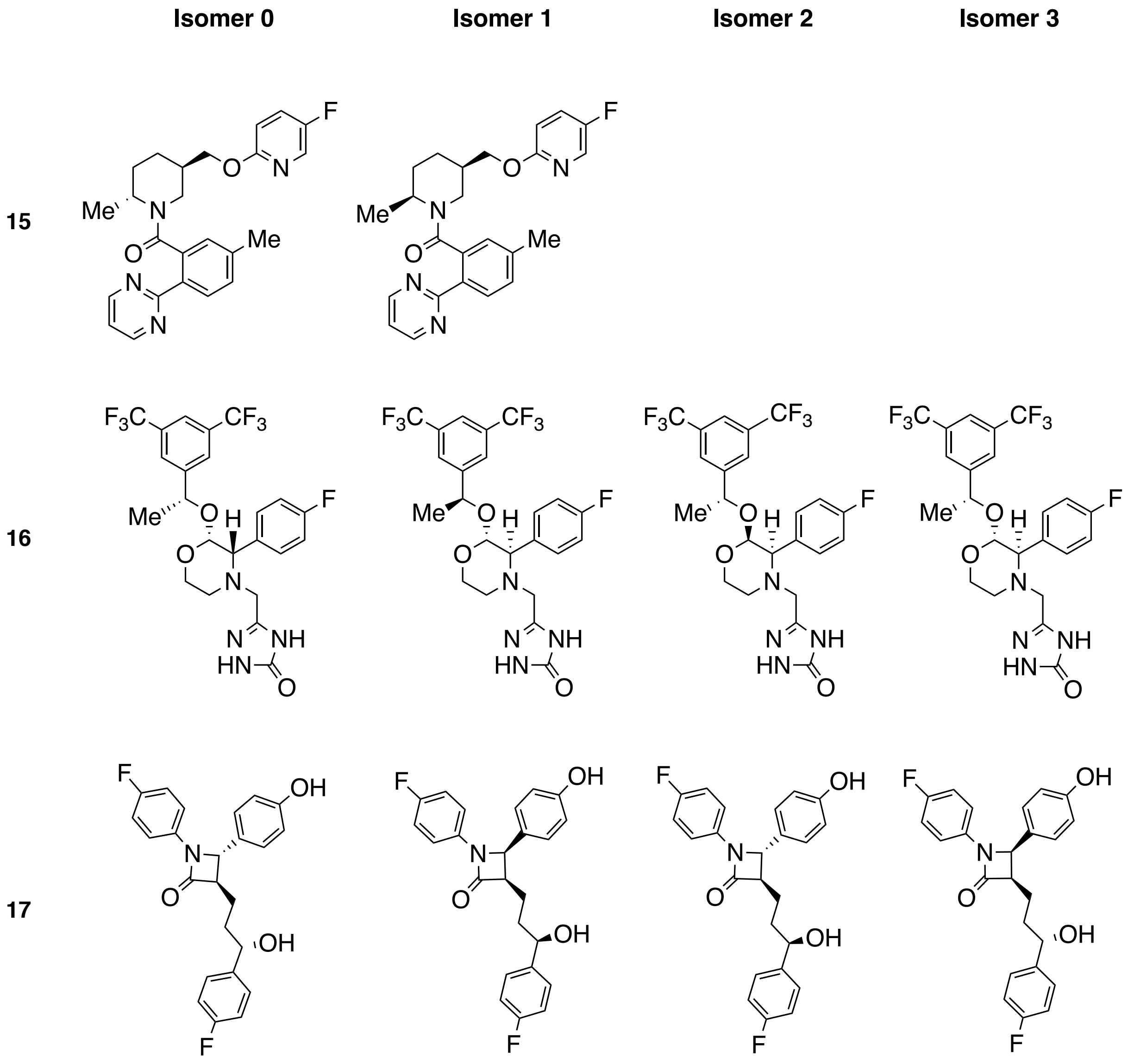

Figure S3: Isomer definition used for compounds **15**-**17**.

2 Correlation Plots

2.1 Compound 2

2.1.1 Alignment Alignment Score s and Combined Score

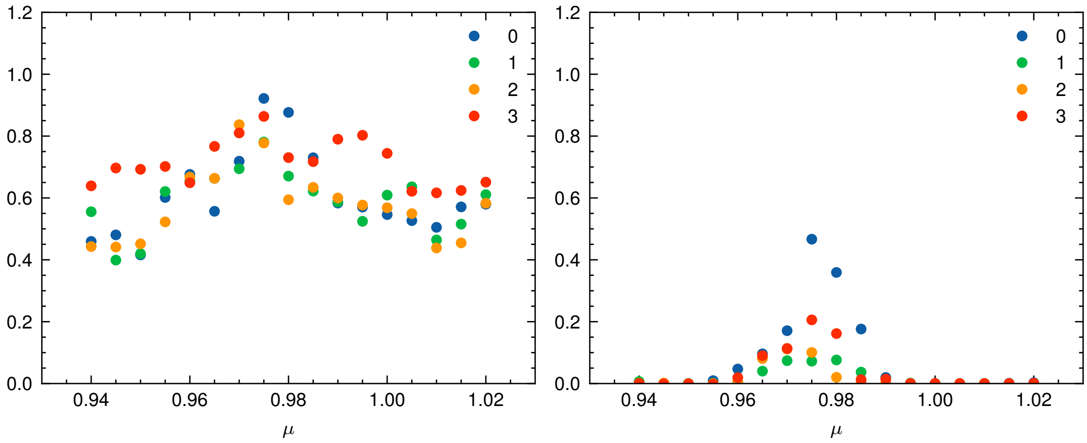

Figure S4: Evaluation metrics as a function of the scaling factor μ for the isomers of compound **2**: total alignment score s (left) and combined score s_{comb} (right).

2.1.2 Pearson Correlation Coefficient

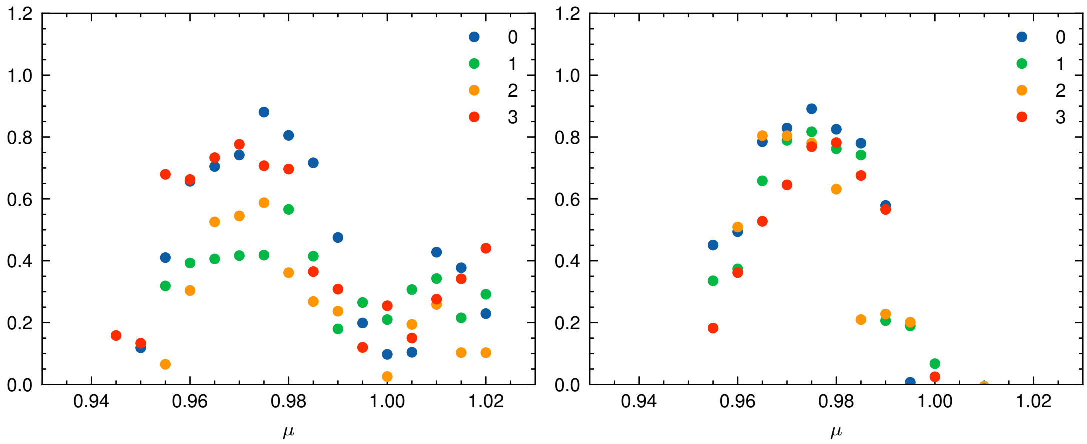

Figure S5: Evaluation metrics as a function of the scaling factor μ for the isomers of compound **2**: Pearson correlation coefficient for IR (left) and Raman (right).

2.1.3 Spearman Correlation Coefficient

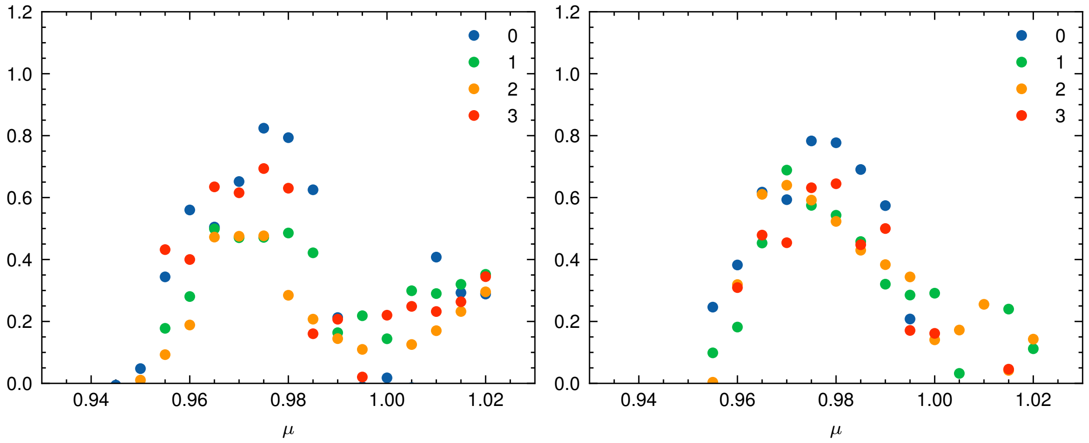

Figure S6: Evaluation metrics as a function of the scaling factor μ for the isomers of compound **2**: Spearman correlation coefficient for IR (left) and Raman (right).

2.2 Compound 3

2.2.1 Alignment Score s and Combined Score

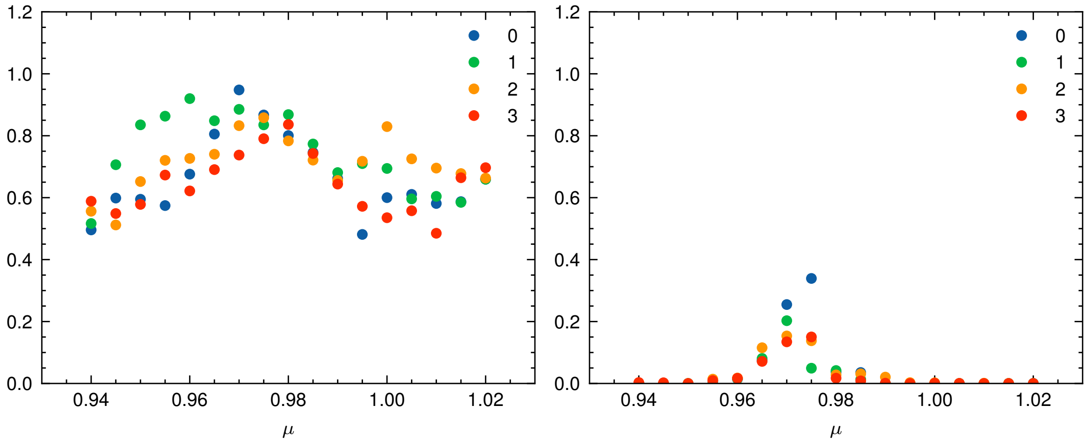

Figure S7: Evaluation metrics as a function of the scaling factor μ for the isomers of compound **3**: total alignment score s (left) and combined score s_{comb} (right).

2.2.2 Pearson Correlation Coefficient

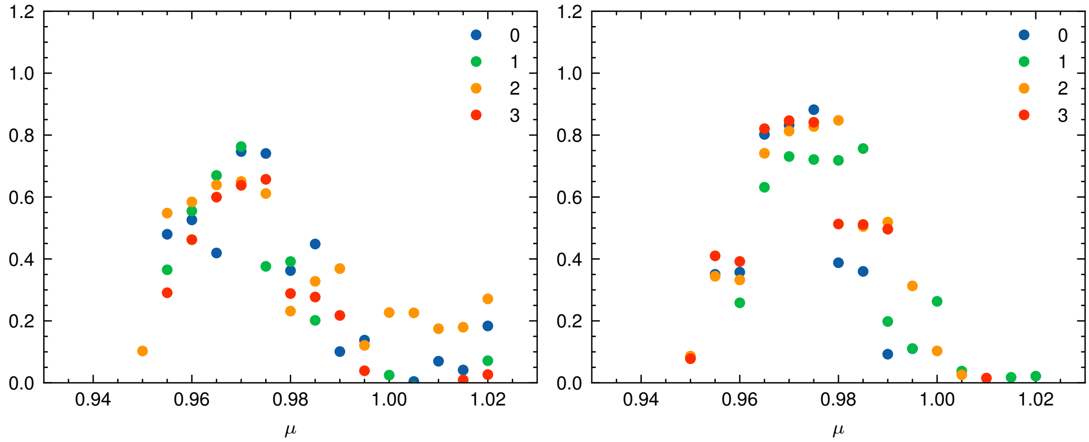

Figure S8: Evaluation metrics as a function of the scaling factor μ for the isomers of compound **3**: Pearson correlation coefficient for IR (left) and Raman (right).

2.2.3 Spearman Correlation Coefficient

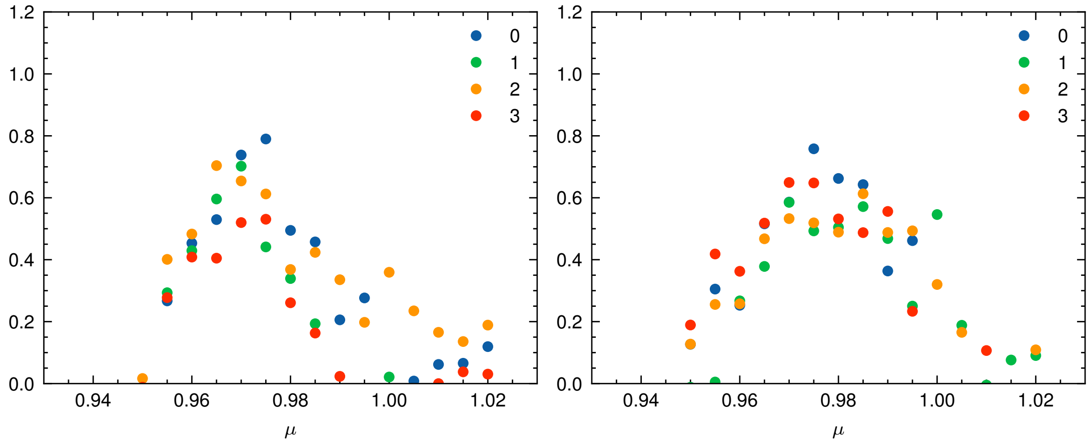

Figure S9: Evaluation metrics as a function of the scaling factor μ for the isomers of compound **3**: Spearman correlation coefficient for IR (left) and Raman (right).

2.3 Compound 4

2.3.1 Alignment Score s and Combined Score

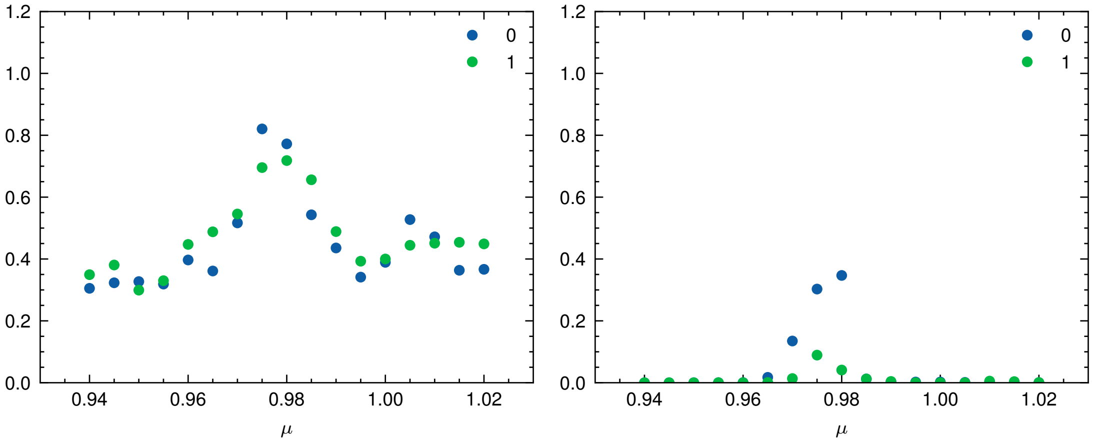

Figure S10: Evaluation metrics as a function of the scaling factor μ for the isomers of compound **4**: total alignment score s (left) and combined score s_{comb} (right).

2.3.2 Pearson Correlation Coefficient

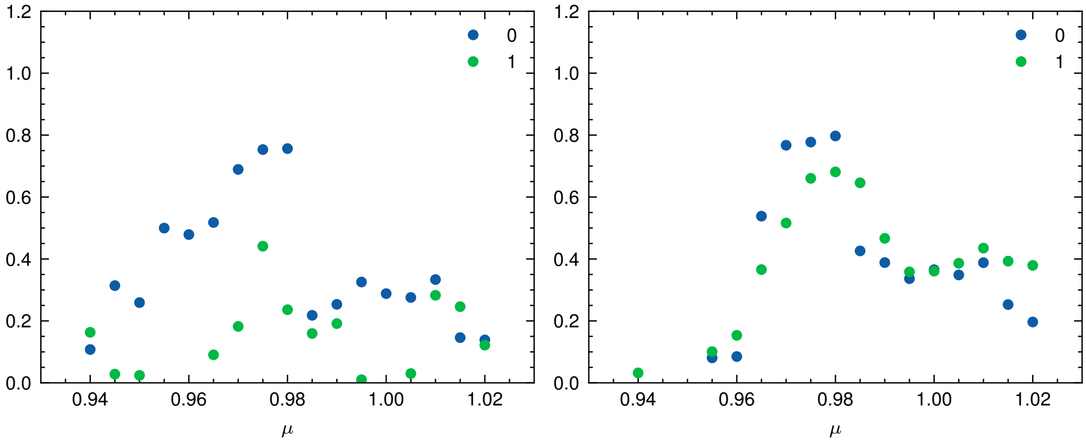

Figure S11: Evaluation metrics as a function of the scaling factor μ for the isomers of compound **4**: Pearson correlation coefficient for IR (left) and Raman (right).

2.3.3 Spearman Correlation Coefficient

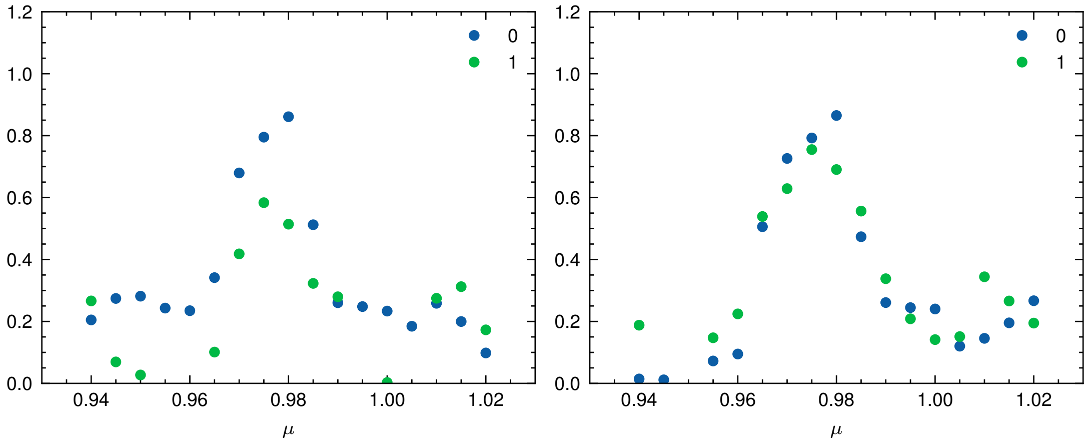

Figure S12: Evaluation metrics as a function of the scaling factor μ for the isomers of compound **4**: Spearman correlation coefficient for IR (left) and Raman (right).

2.4 Compound 5

2.4.1 Alignment Score s and Combined Score

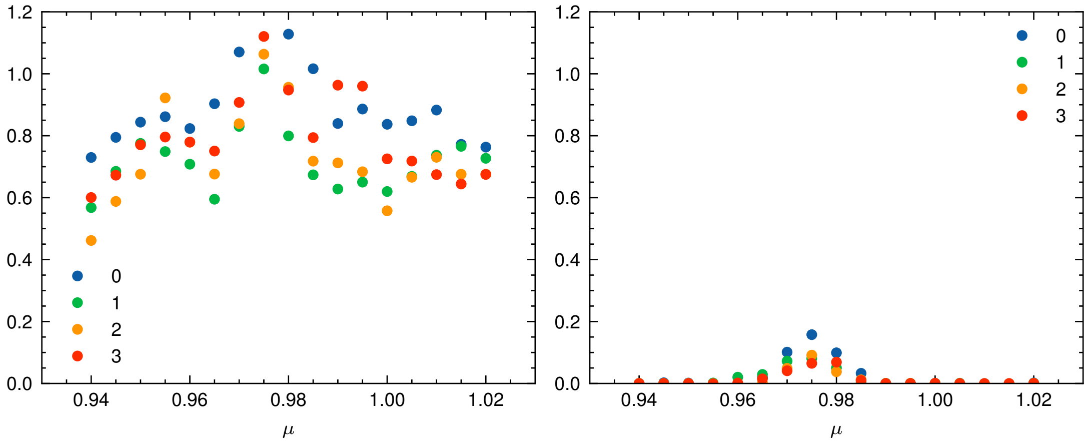

Figure S13: Evaluation metrics as a function of the scaling factor μ for the isomers of compound **5**: total alignment score s (left) and combined score s_{comb} (right).

2.4.2 Pearson Correlation Coefficient

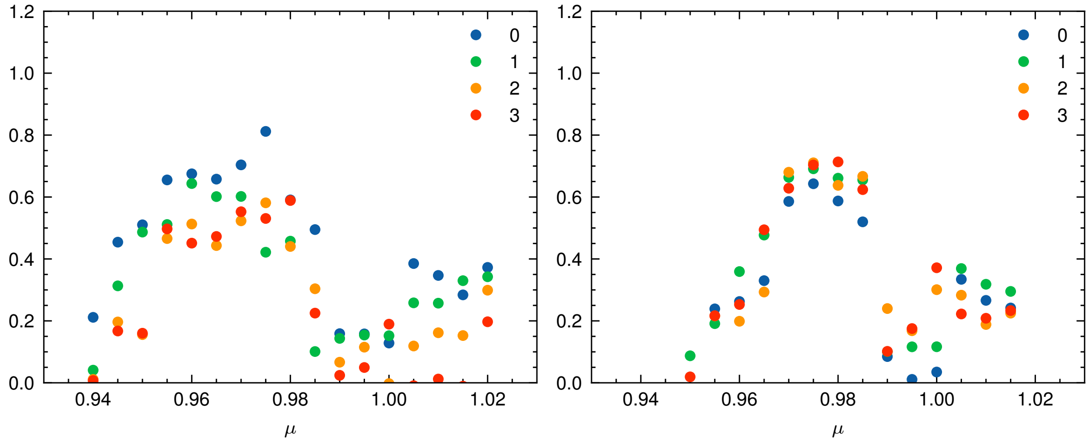

Figure S14: Evaluation metrics as a function of the scaling factor μ for the isomers of compound **5**: Pearson correlation coefficient for IR (left) and Raman (right).

2.4.3 Spearman Correlation Coefficient

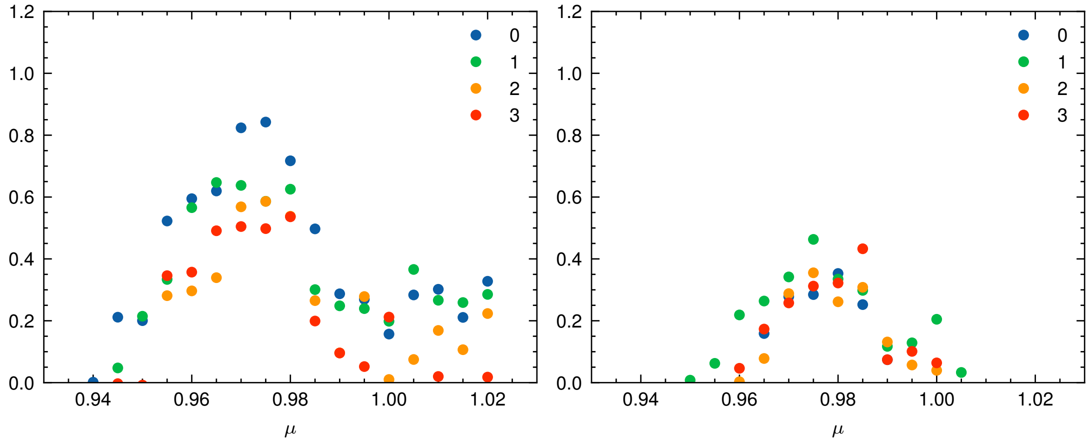

Figure S15: Evaluation metrics as a function of the scaling factor μ for the isomers of compound **5**: Spearman correlation coefficient for IR (left) and Raman (right).

2.5 Compound 6

2.5.1 Alignment Score s and Combined Score

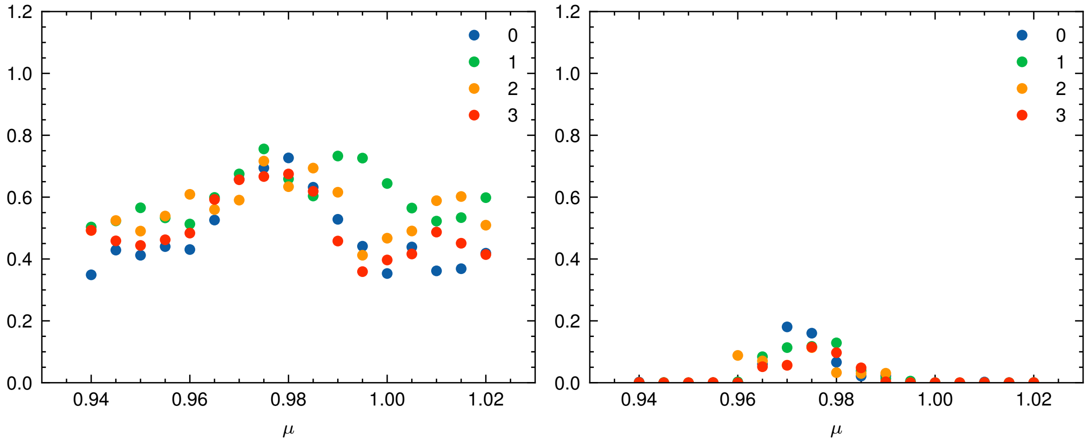

Figure S16: Evaluation metrics as a function of the scaling factor μ for the isomers of compound **6**: total alignment score s (left) and combined score s_{comb} (right).

2.5.2 Pearson Correlation Coefficient

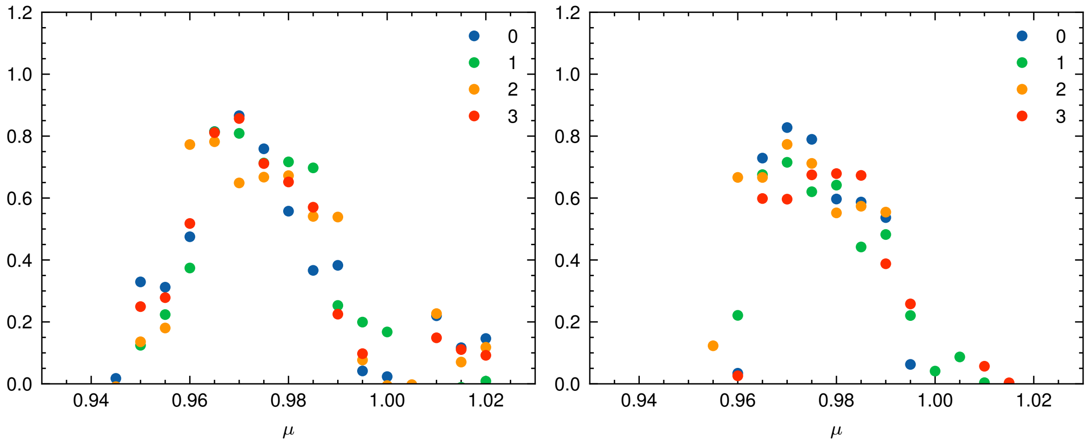

Figure S17: Evaluation metrics as a function of the scaling factor μ for the isomers of compound **6**: Pearson correlation coefficient for IR (left) and Raman (right).

2.5.3 Spearman Correlation Coefficient

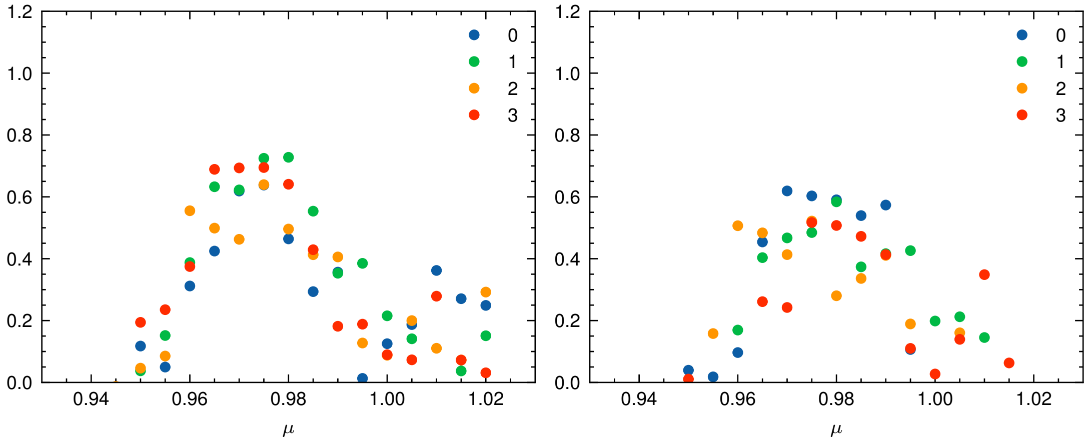

Figure S18: Evaluation metrics as a function of the scaling factor μ for the isomers of compound **6**: Spearman correlation coefficient for IR (left) and Raman (right).

2.6 Compound 7

2.6.1 Alignment Score s and Combined Score

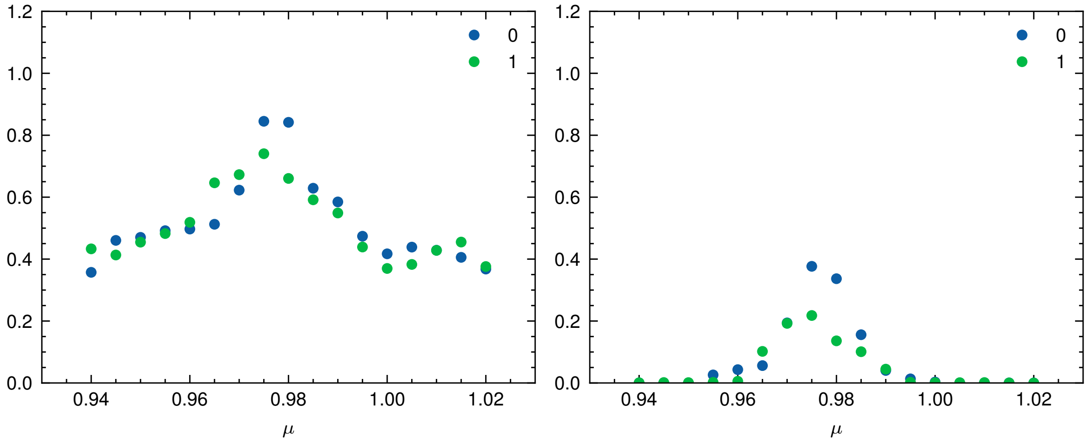

Figure S19: Evaluation metrics as a function of the scaling factor μ for the isomers of compound **7**: total alignment score s (left) and combined score s_{comb} (right).

2.6.2 Pearson Correlation Coefficient

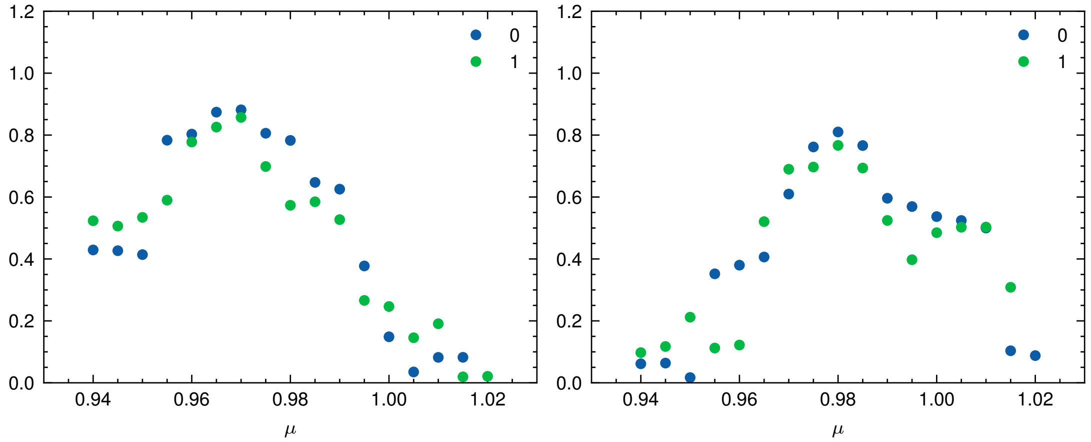

Figure S20: Evaluation metrics as a function of the scaling factor μ for the isomers of compound **7**: Pearson correlation coefficient for IR (left) and Raman (right).

2.6.3 Spearman Correlation Coefficient

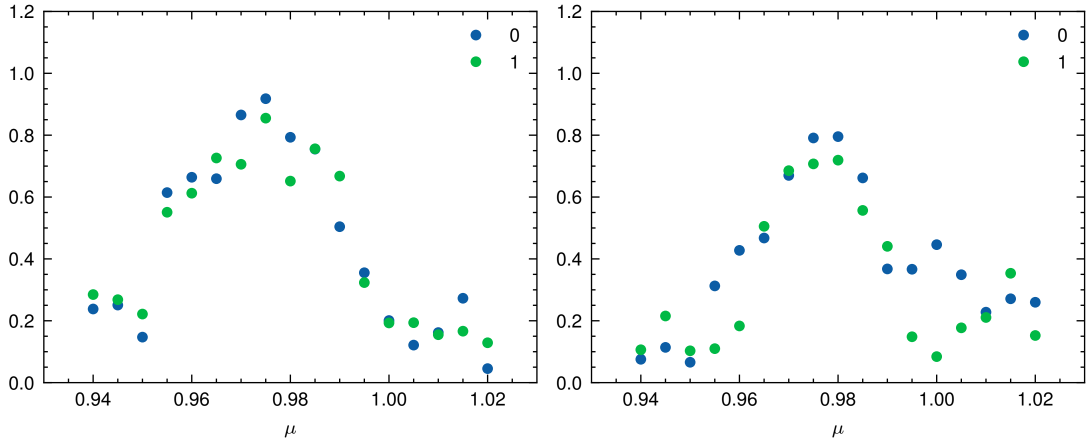

Figure S21: Evaluation metrics as a function of the scaling factor μ for the isomers of compound **7**: Spearman correlation coefficient for IR (left) and Raman (right).

2.7 Compound 8

2.7.1 Alignment Score s and Combined Score

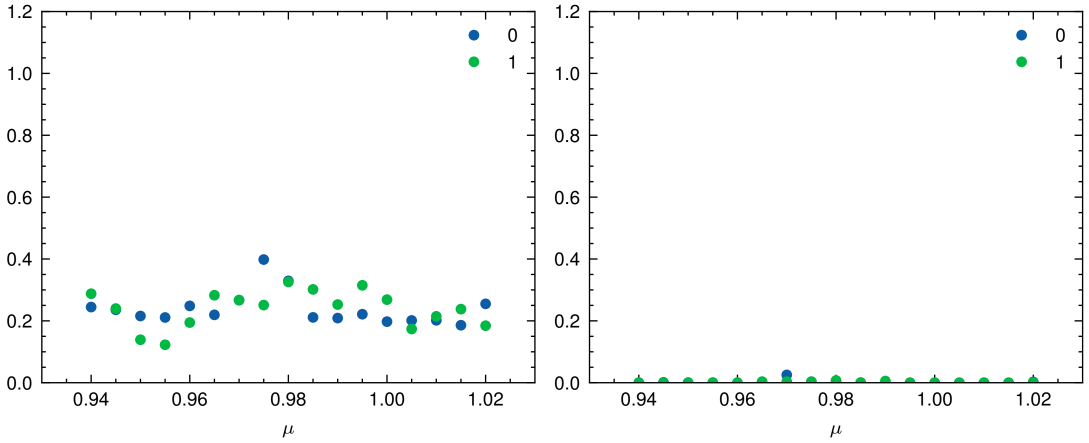

Figure S22: Evaluation metrics as a function of the scaling factor μ for the isomers of compound **2**: total alignment score s (left) and combined score s_{comb} (right).

2.7.2 Pearson Correlation Coefficient

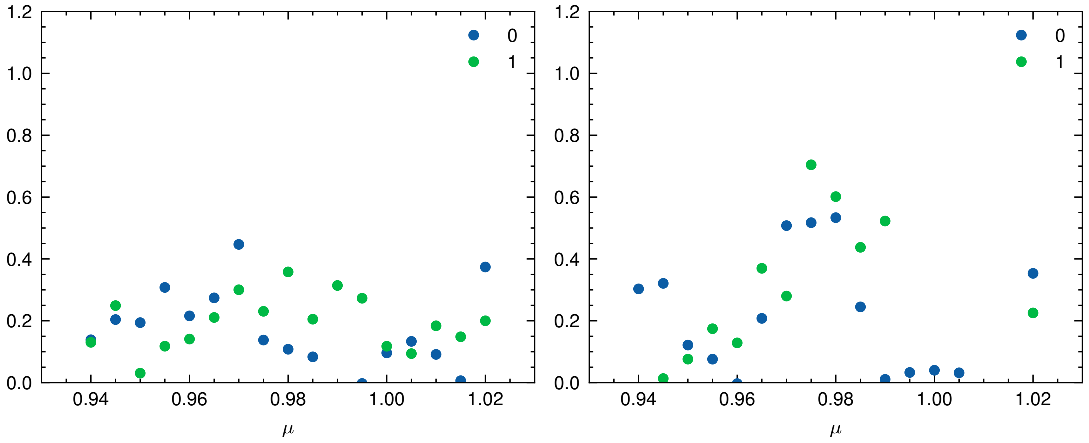

Figure S23: Evaluation metrics as a function of the scaling factor μ for the isomers of compound **8**: Pearson correlation coefficient for IR (left) and Raman (right).

2.7.3 Spearman Correlation Coefficient

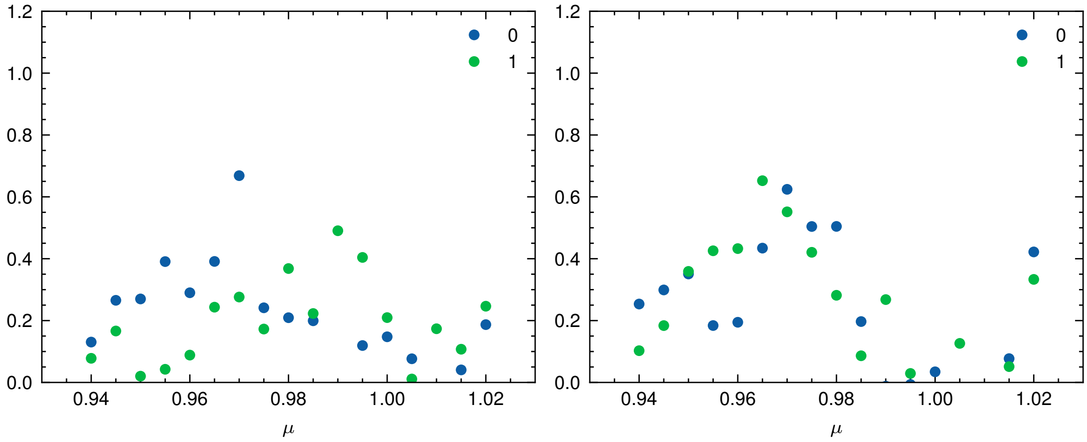

Figure S24: Evaluation metrics as a function of the scaling factor μ for the isomers of compound **8**: Spearman correlation coefficient for IR (left) and Raman (right).

2.8 Compound 9

2.8.1 Alignment Score s and Combined Score

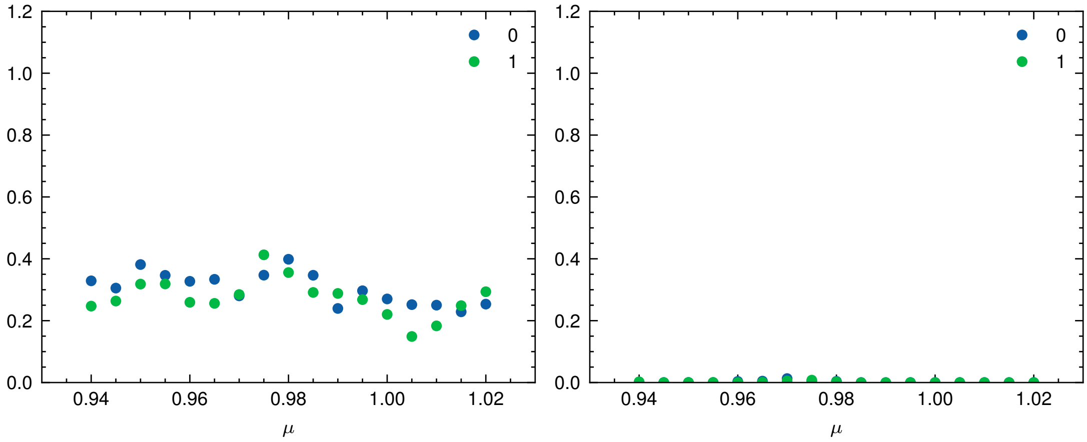

Figure S25: Evaluation metrics as a function of the scaling factor μ for the isomers of compound **9**: total alignment score s (left) and combined score s_{comb} (right).

2.8.2 Pearson Correlation Coefficient

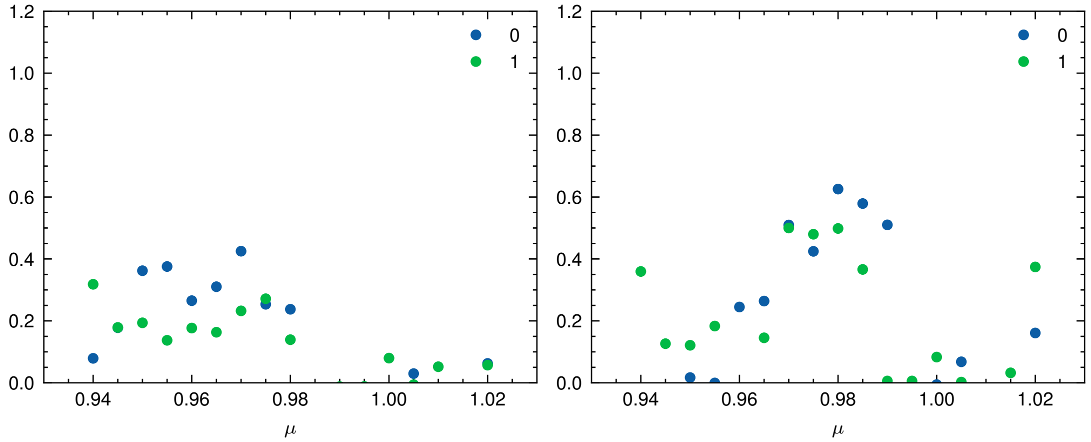

Figure S26: Evaluation metrics as a function of the scaling factor μ for the isomers of compound **9**: Pearson correlation coefficient for IR (left) and Raman (right).

2.8.3 Spearman Correlation Coefficient

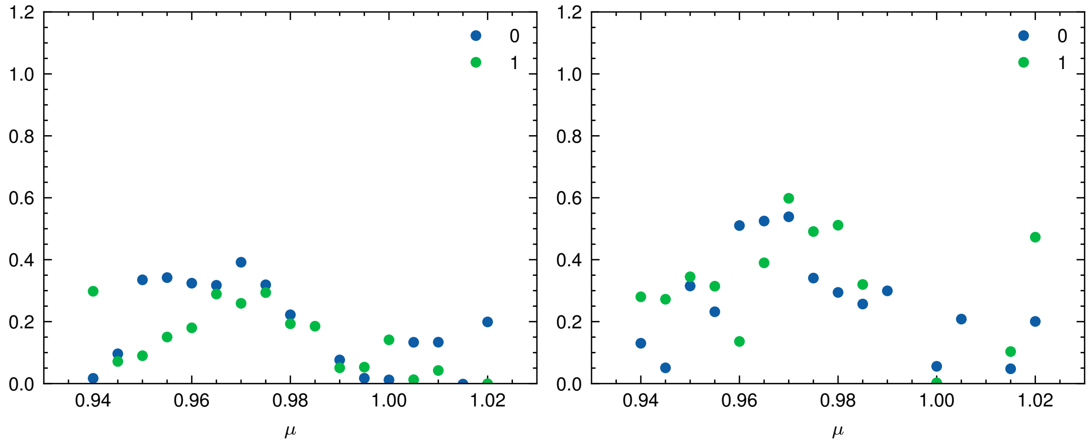

Figure S27: Evaluation metrics as a function of the scaling factor μ for the isomers of compound **9**: Spearman correlation coefficient for IR (left) and Raman (right).

2.9 Compound 10

2.9.1 Alignment Score s and Combined Score

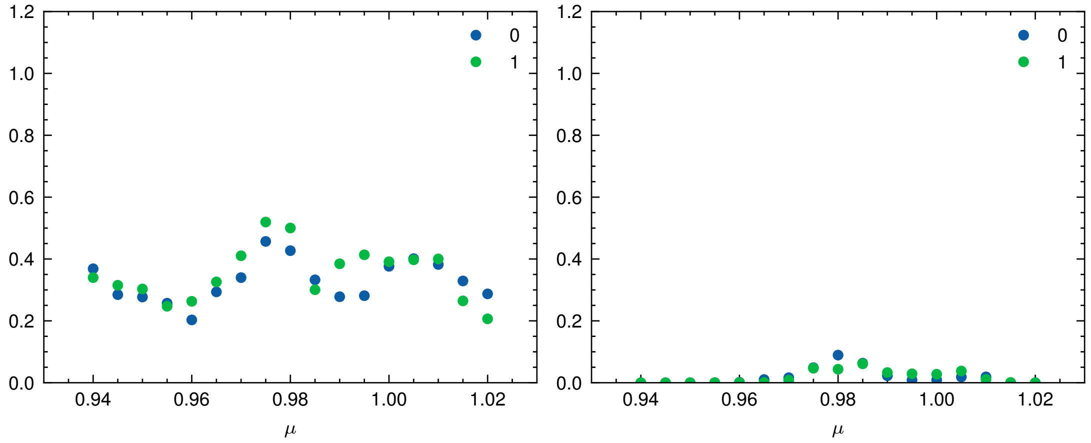

Figure S28: Evaluation metrics as a function of the scaling factor μ for the isomers of compound **10**: total alignment score s (left) and combined score s_{comb} (right).

2.9.2 Pearson Correlation Coefficient

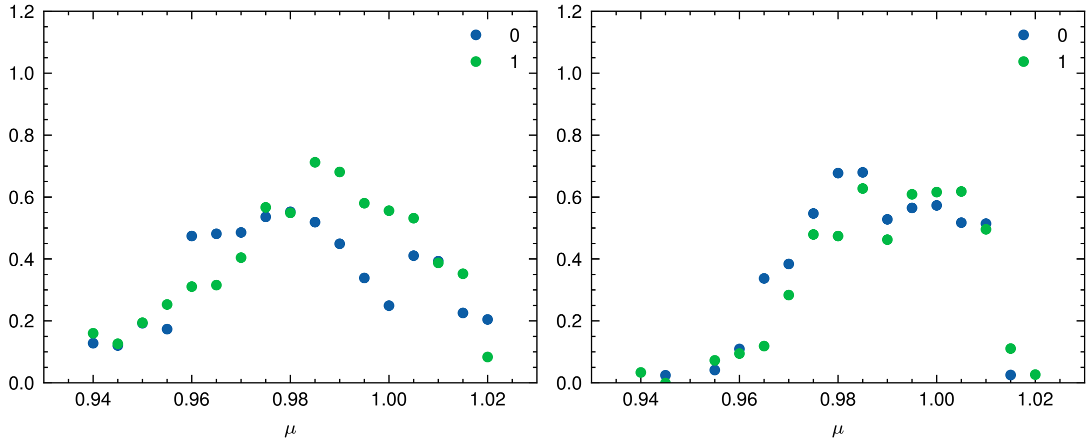

Figure S29: Evaluation metrics as a function of the scaling factor μ for the isomers of compound **10**: Pearson correlation coefficient for IR (left) and Raman (right).

2.9.3 Spearman Correlation Coefficient

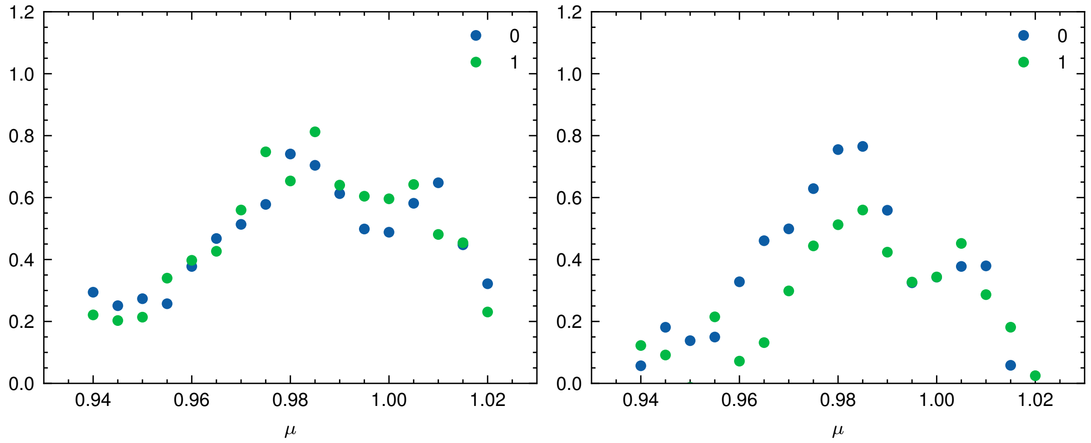

Figure S30: Evaluation metrics as a function of the scaling factor μ for the isomers of compound **10**: Spearman correlation coefficient for IR (left) and Raman (right).

2.10 Compound 11

2.10.1 Alignment Score s and Combined Score

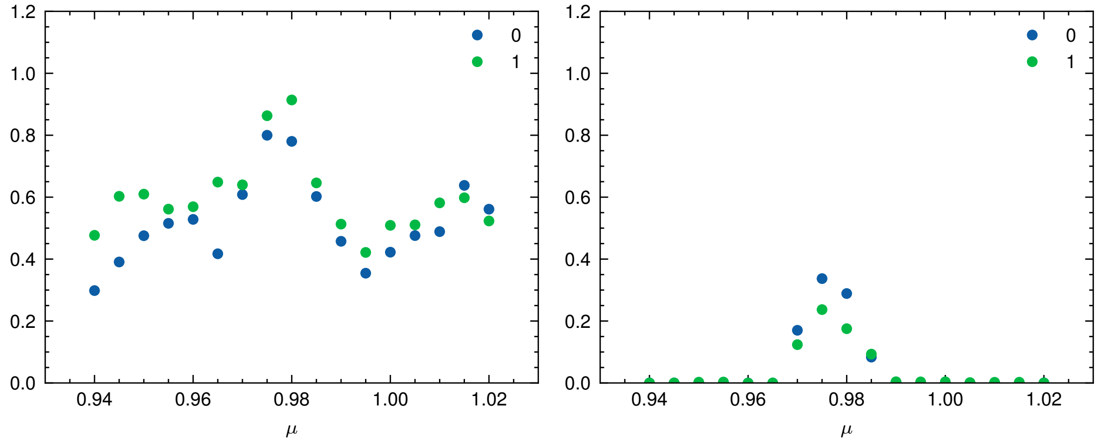

Figure S31: Evaluation metrics as a function of the scaling factor μ for the isomers of compound **11**: total alignment score s (left) and combined score s_{comb} (right).

2.10.2 Pearson Correlation Coefficient

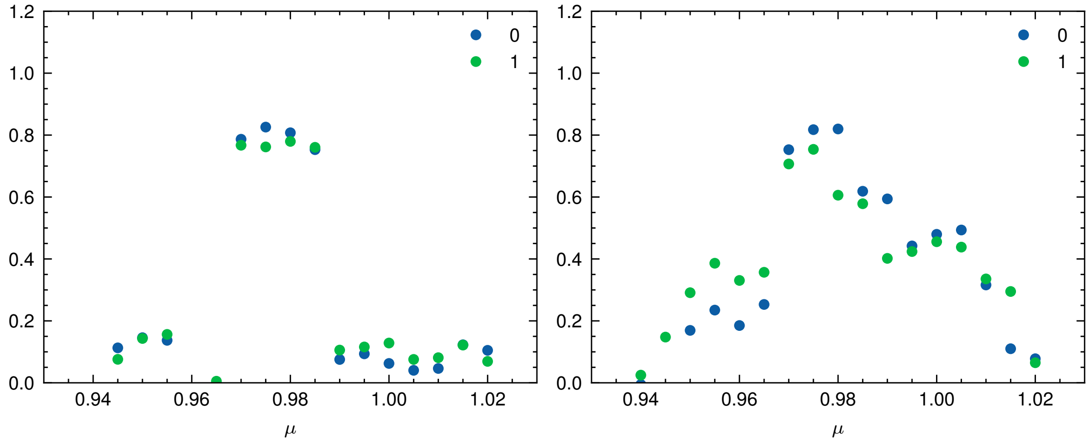

Figure S32: Evaluation metrics as a function of the scaling factor μ for the isomers of compound **11**: Pearson correlation coefficient for IR (left) and Raman (right).

2.10.3 Spearman Correlation Coefficient

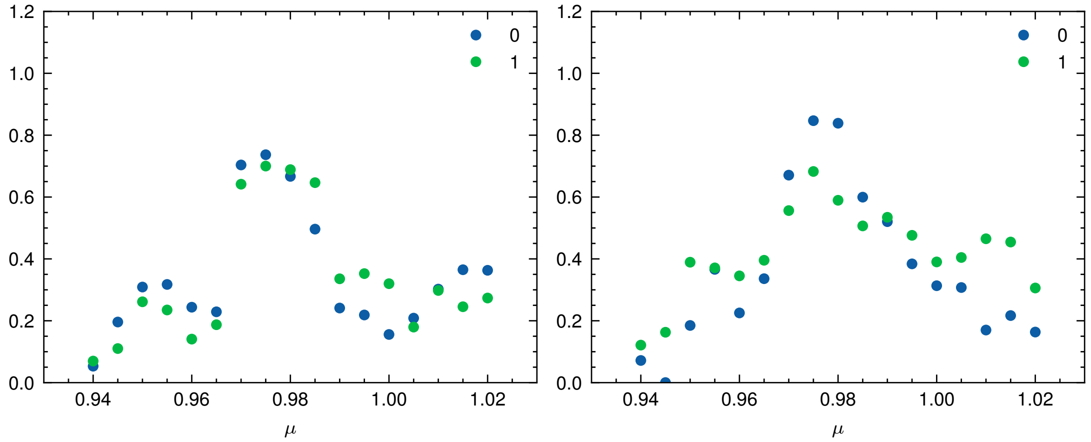

Figure S33: Evaluation metrics as a function of the scaling factor μ for the isomers of compound **11**: Spearman correlation coefficient for IR (left) and Raman (right).

2.11 Compound 12

2.11.1 Alignment Score s and Combined Score

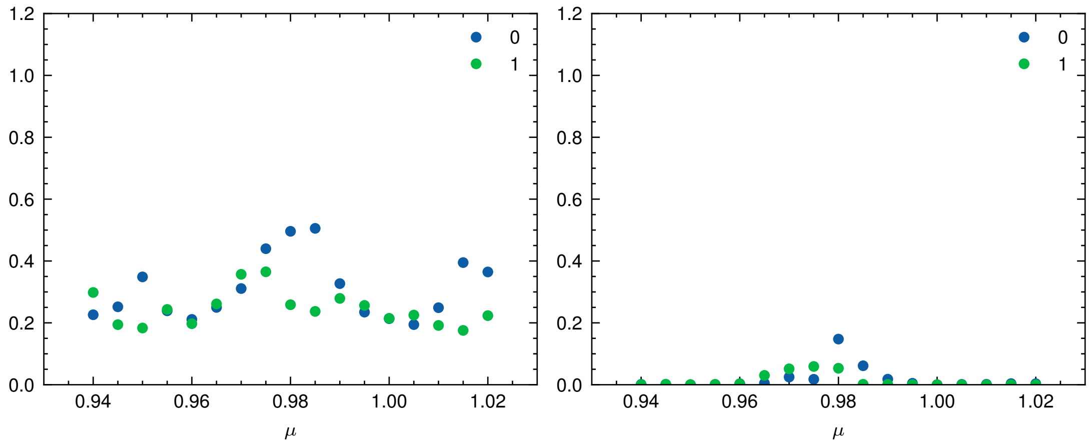

Figure S34: Evaluation metrics as a function of the scaling factor μ for the isomers of compound **12**: total alignment score s (left) and combined score s_{comb} (right).

2.11.2 Pearson Correlation Coefficient

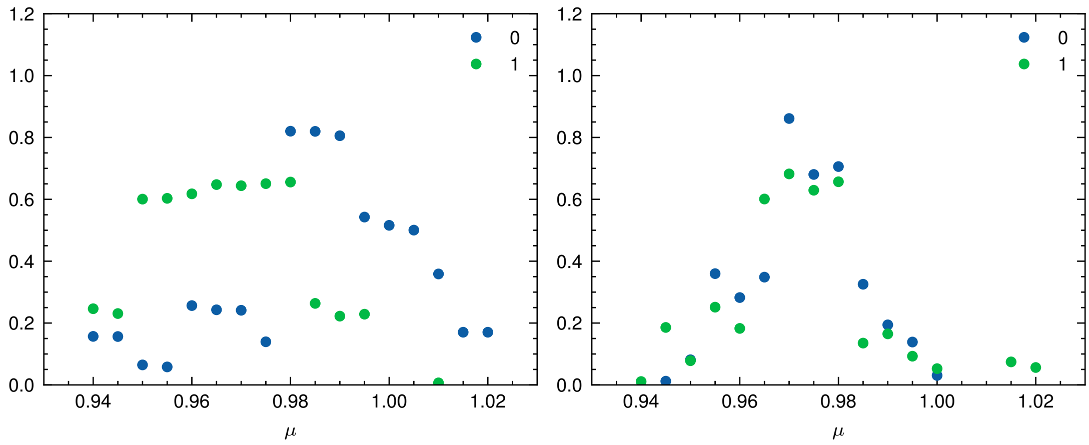

Figure S35: Evaluation metrics as a function of the scaling factor μ for the isomers of compound **12**: Pearson correlation coefficient for IR (left) and Raman (right).

2.11.3 Spearman Correlation Coefficient

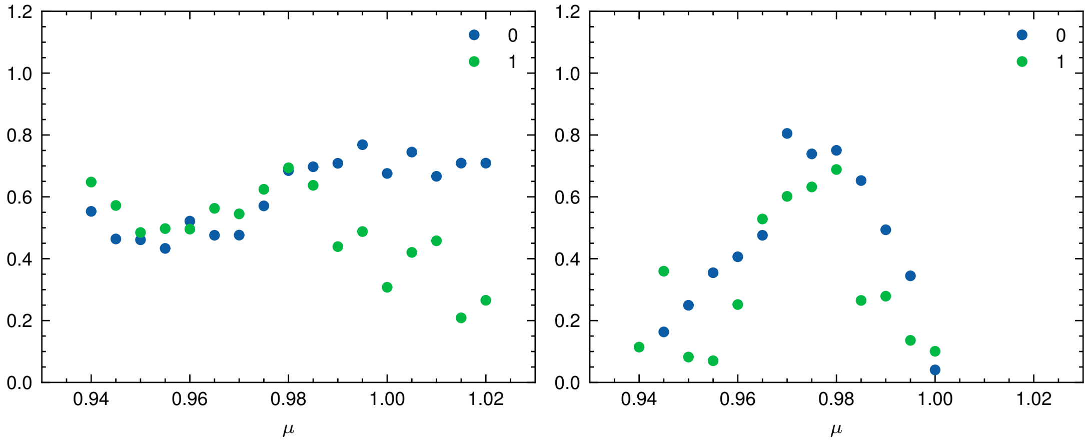

Figure S36: Evaluation metrics as a function of the scaling factor μ for the isomers of compound **12**: Spearman correlation coefficient for IR (left) and Raman (right).

2.12 Compound 13

2.12.1 Alignment Score s and Combined Score

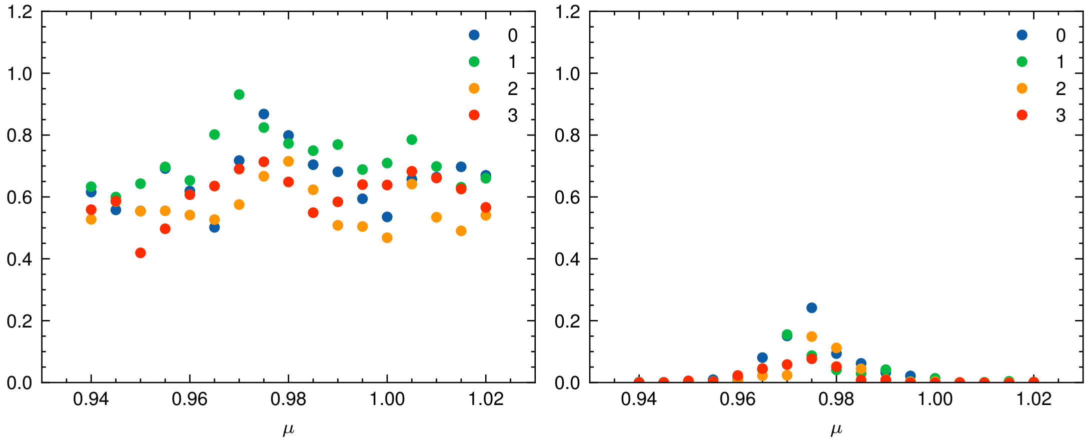

Figure S37: Evaluation metrics as a function of the scaling factor μ for the isomers of compound **13**: total alignment score s (left) and combined score s_{comb} (right).

2.12.2 Pearson Correlation Coefficient

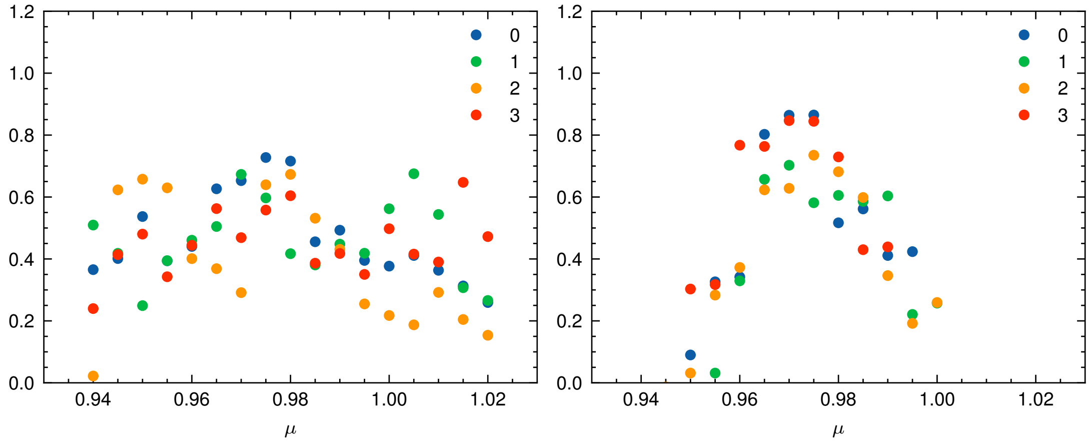

Figure S38: Evaluation metrics as a function of the scaling factor μ for the isomers of compound **13**: Pearson correlation coefficient for IR (left) and Raman (right).

2.12.3 Spearman Correlation Coefficient

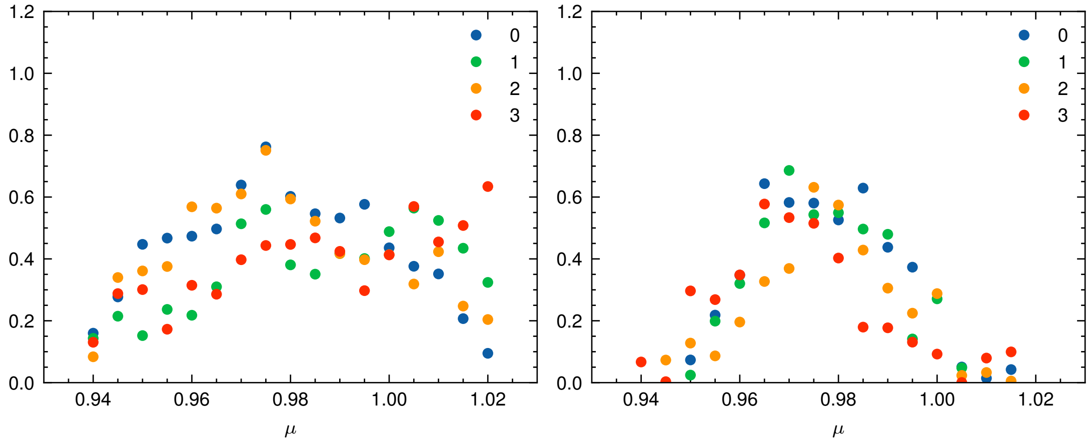

Figure S39: Evaluation metrics as a function of the scaling factor μ for the isomers of compound **13**: Spearman correlation coefficient for IR (left) and Raman (right).

2.13 Compound 14

2.13.1 Alignment Score s and Combined Score

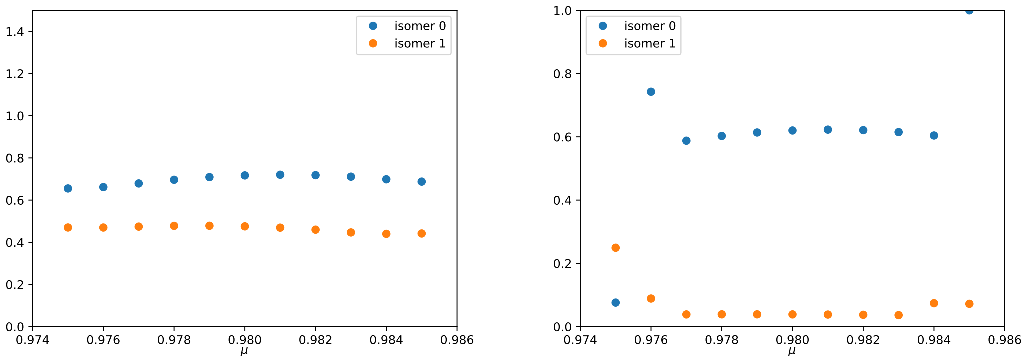

Figure S40: Evaluation metrics as a function of the scaling factor μ for the isomers of compound **14**: total alignment score s (left) and combined score s_{comb} (right).

2.13.2 Pearson Correlation Coefficient

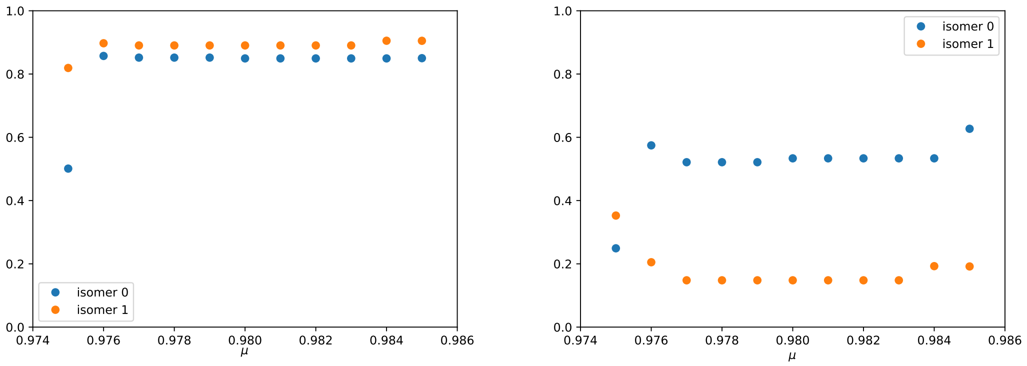

Figure S41: Evaluation metrics as a function of the scaling factor μ for the isomers of compound **14**: Pearson correlation coefficient for IR (left) and Raman (right).

2.13.3 Spearman Correlation Coefficient

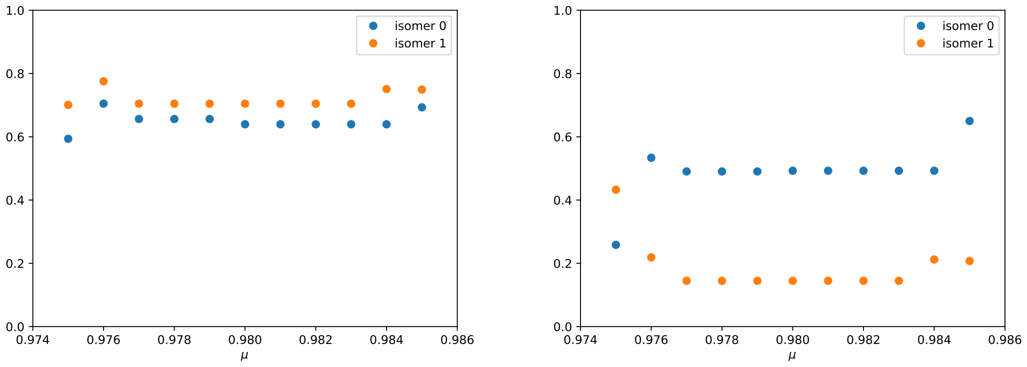

Figure S42: Evaluation metrics as a function of the scaling factor μ for the isomers of compound **14**: Spearman correlation coefficient for IR (left) and Raman (right).

2.14 VCD spectra

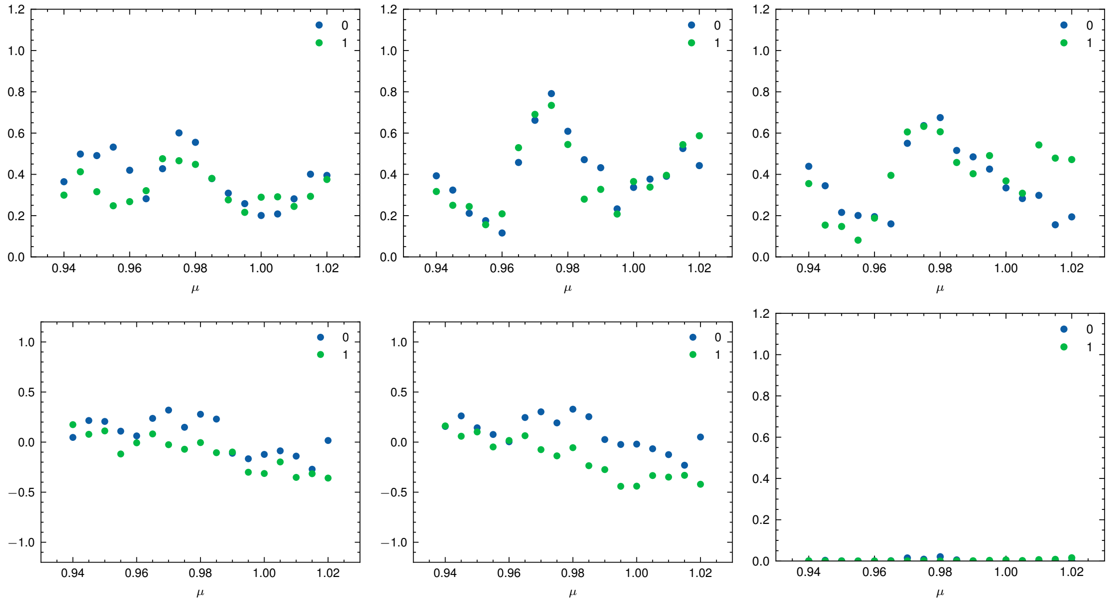

Figure S43: Evaluation metrics as a function of the scaling factor μ for the isomers of compound **15**: total alignment score s (top left), combined score s_{comb} (bottom right), Pearson and Spearman correlation coefficients (remaining panels) for the IR and VCD spectra.

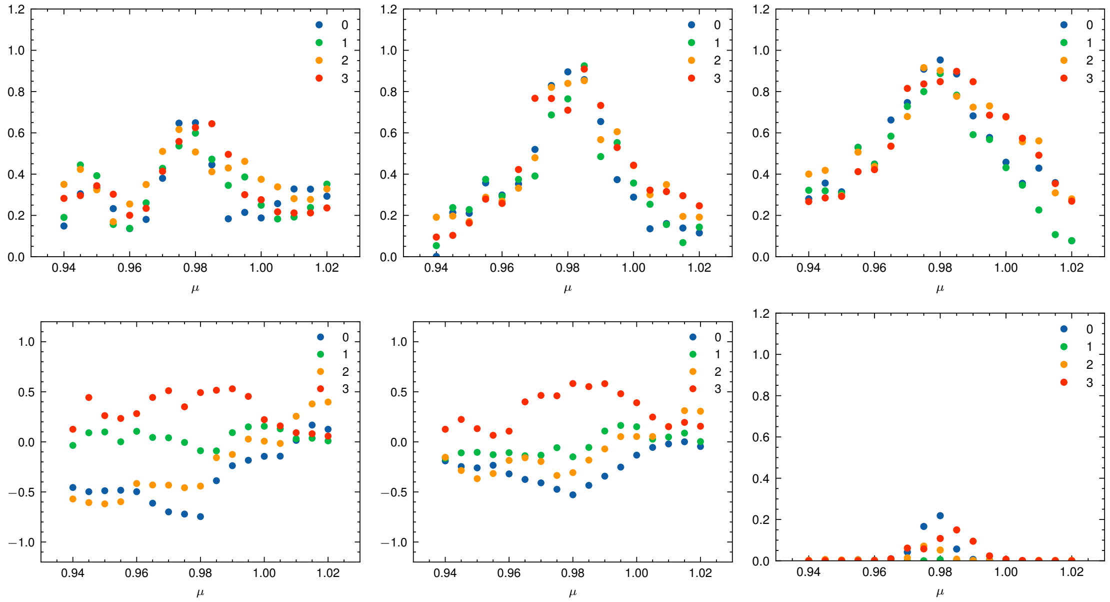

Figure S44: Evaluation metrics as a function of the scaling factor μ for the isomers of compound **16**: total alignment score s (top left), combined score s_{comb} (bottom right), Pearson and Spearman correlation coefficients (remaining panels) for the IR and VCD spectra.

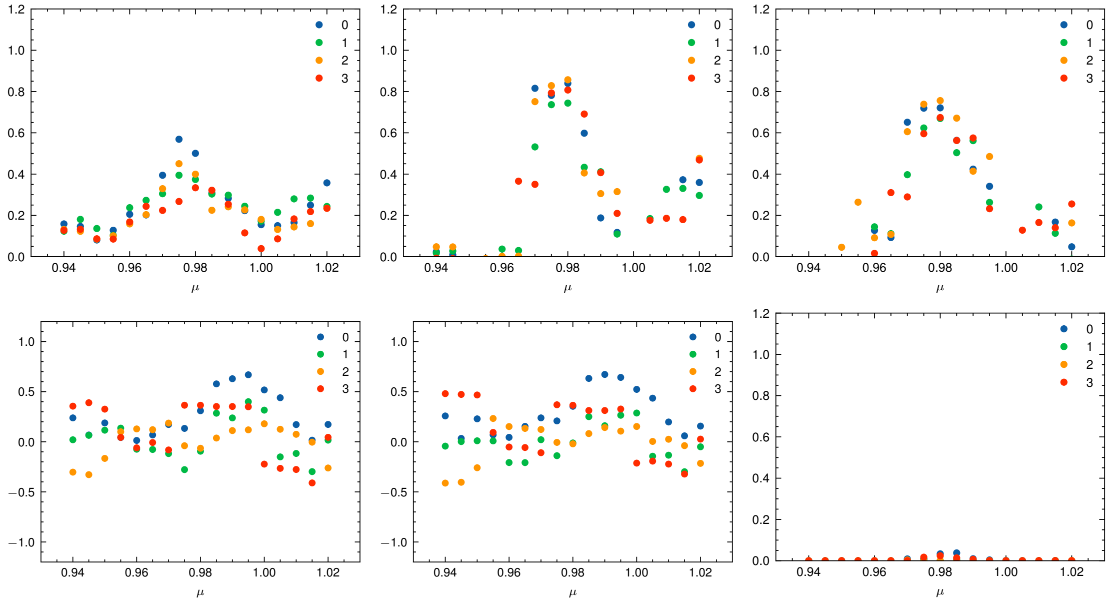

Figure S45: Evaluation metrics as a function of the scaling factor μ for the isomers of compound **17**: total alignment score s (top left), combined score s_{comb} (bottom right), Pearson and Spearman correlation coefficients (remaining panels) for the IR and VCD spectra.

3 Aligned Spectra

All presented results employ $\mu = 0.98$.

3.1 Compound 1

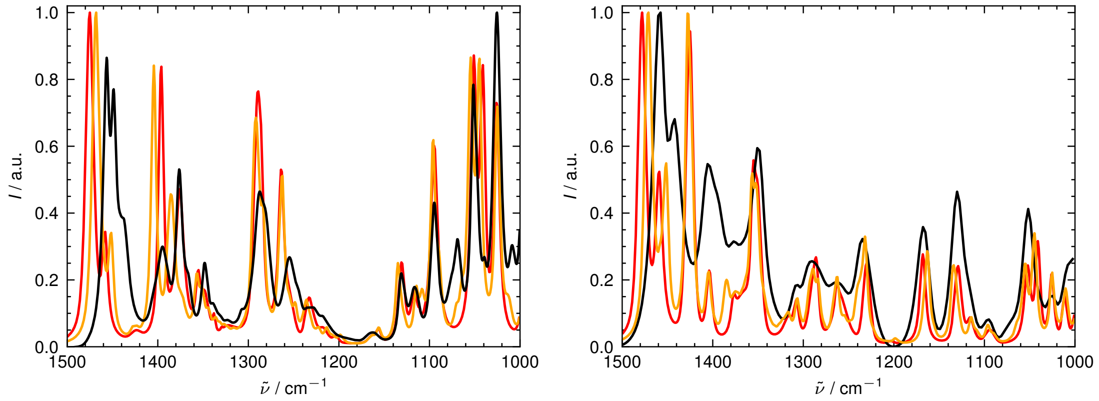

Figure S46: Aligned spectrum of compound **1**, isomer 0. Left: IR, Right: Raman.

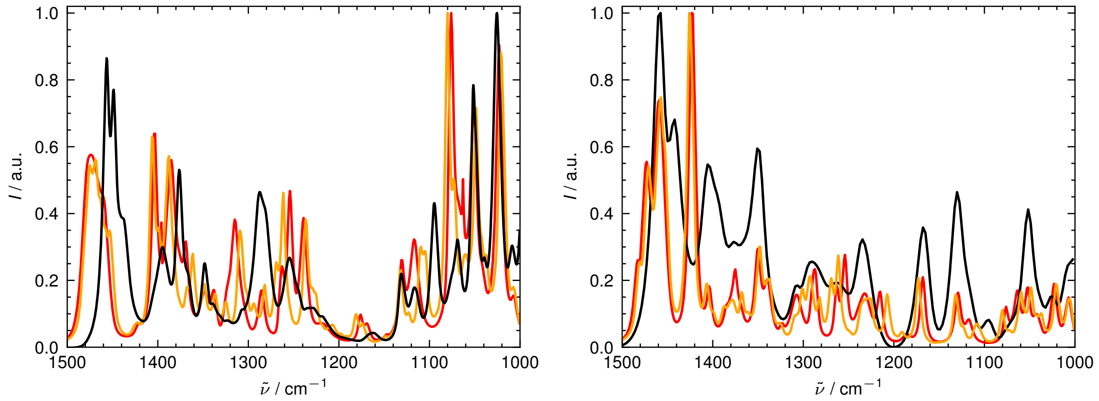

Figure S47: Aligned spectrum of compound **1** isomer 1. Left: IR, Right: Raman.

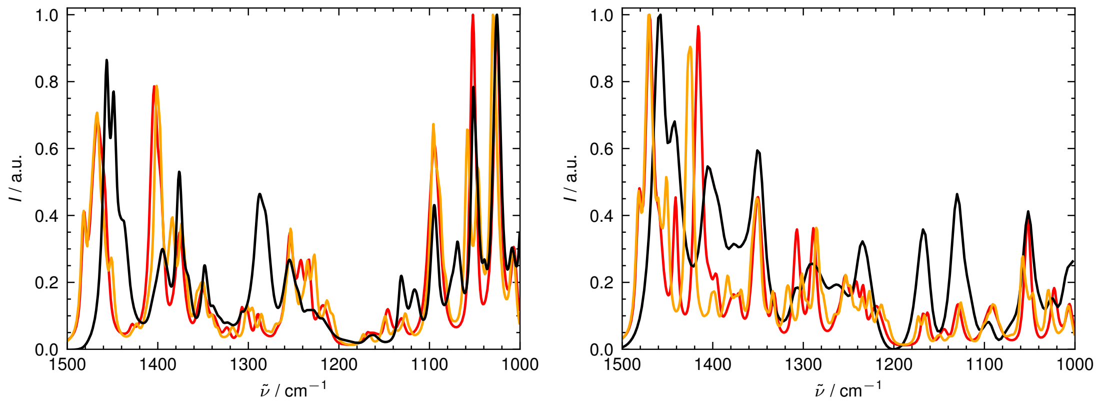

Figure S48: Aligned spectrum of compound **1**, isomer 2. Left: IR, Right: Raman.

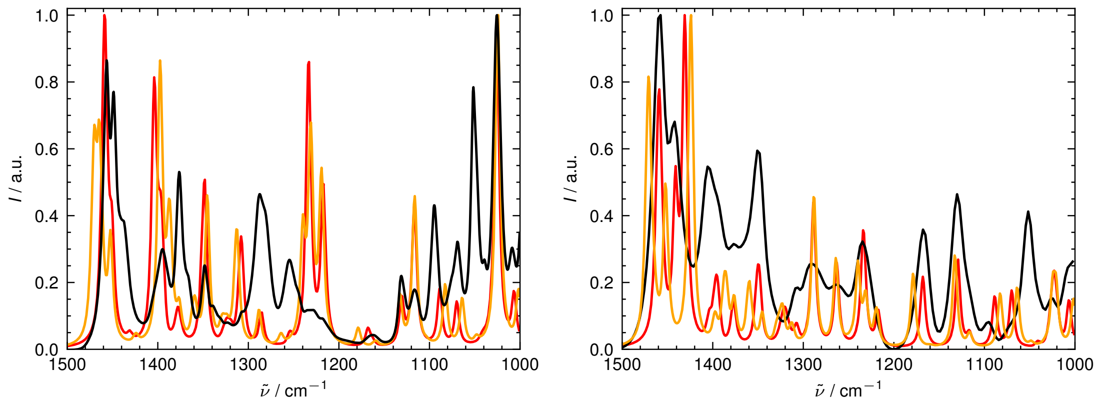

Figure S49: Aligned spectrum of compound **1**, isomer 3. Left: IR, Right: Raman.

3.2 Compound 2

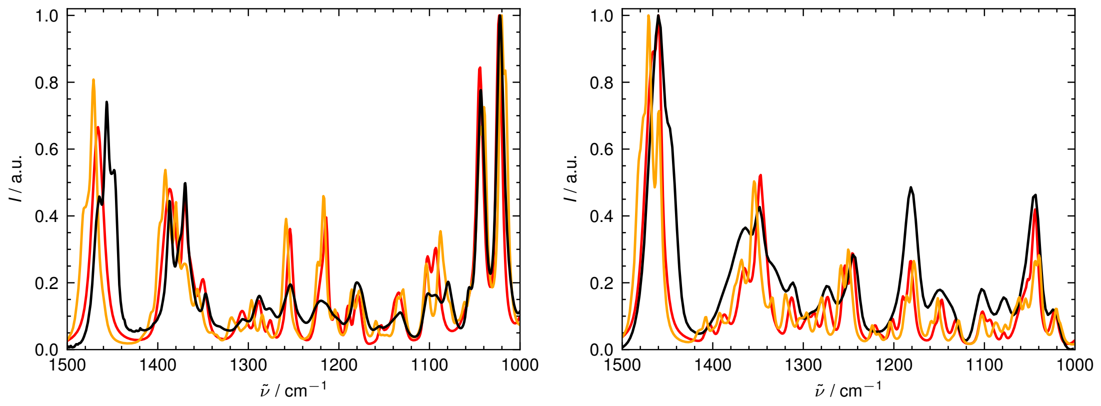

Figure S50: Aligned spectrum of compound **2**, isomer 0. Left: IR, Right: Raman.

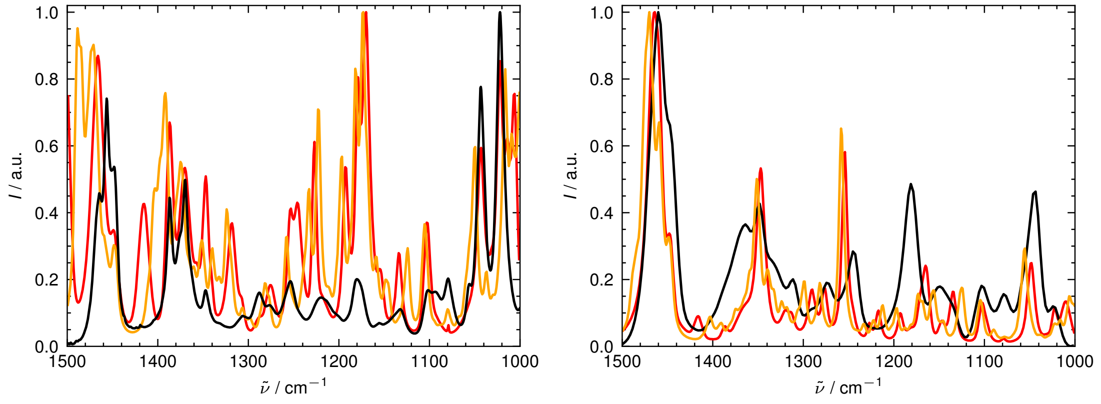

Figure S51: Aligned spectrum of compound **2**, isomer 1. Left: IR, Right: Raman.

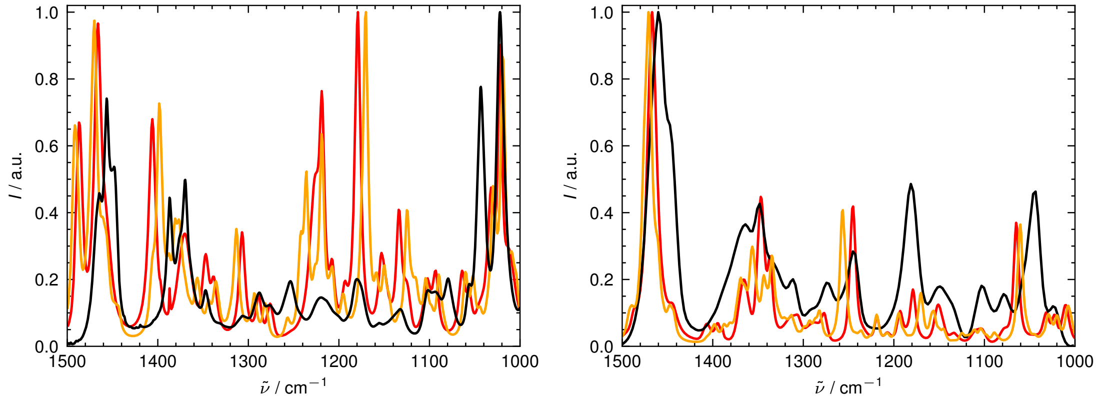

Figure S52: Aligned spectrum of compound **2**, isomer 2. Left: IR, Right: Raman.

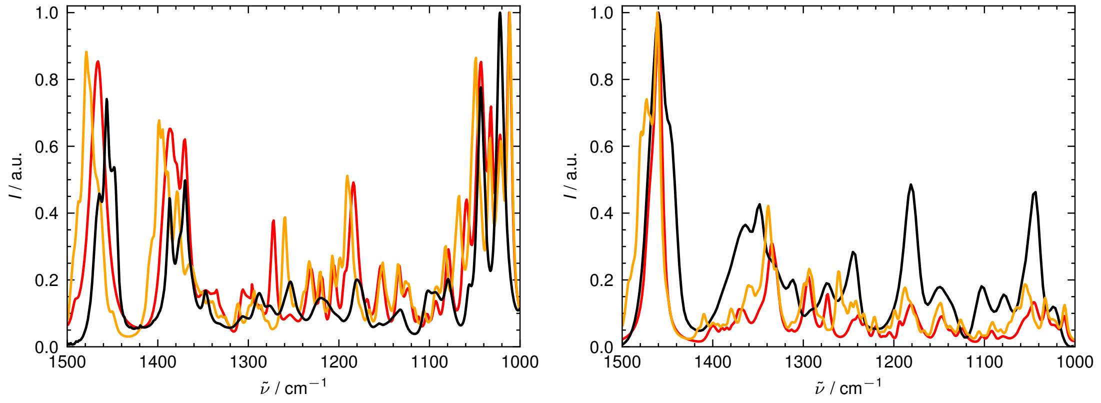

Figure S53: Aligned spectrum of compound **2**, isomer 3. Left: IR, Right: Raman.

3.3 Compound 3

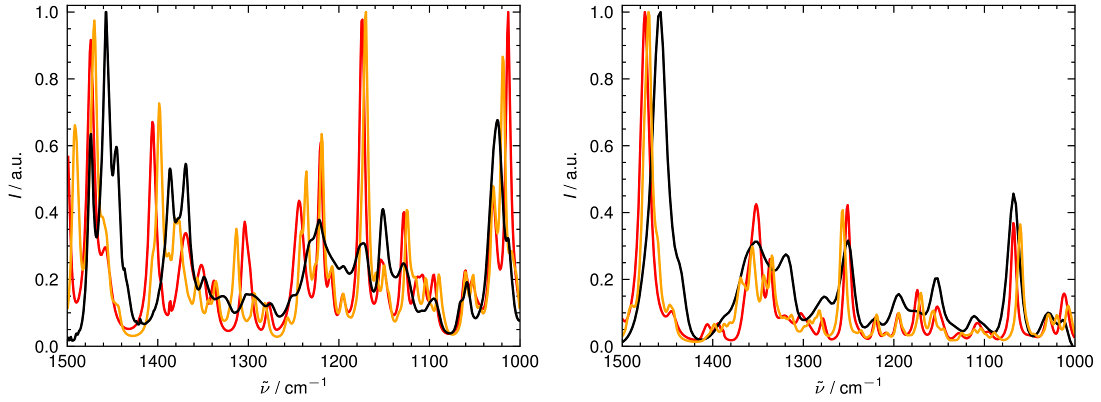

Figure S54: Aligned spectrum of compound **3**, isomer 0. Left: IR, Right: Raman.

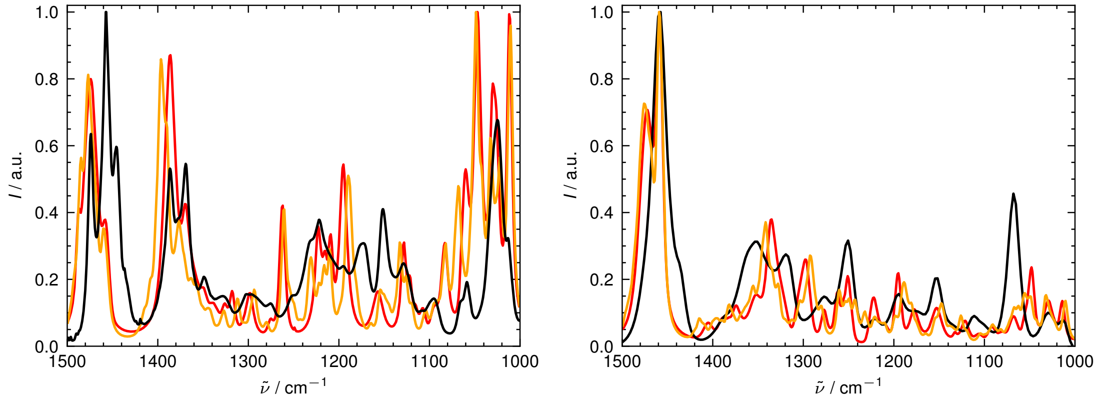

Figure S55: Aligned spectrum of compound **3**, isomer 1. Left: IR, Right: Raman.

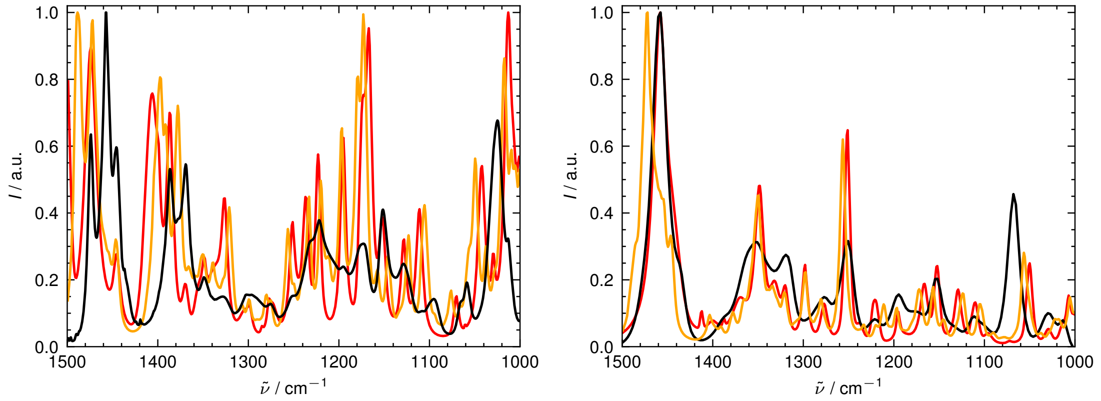

Figure S56: Aligned spectrum of compound **3**, isomer 2. Left: IR, Right: Raman.

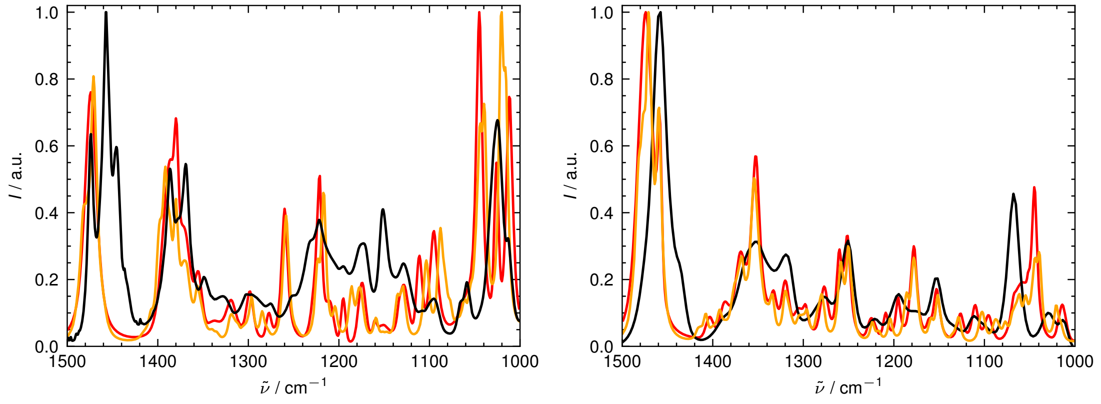

Figure S57: Aligned spectrum of compound **3**, isomer 3. Left: IR, Right: Raman.

3.4 Compound 4

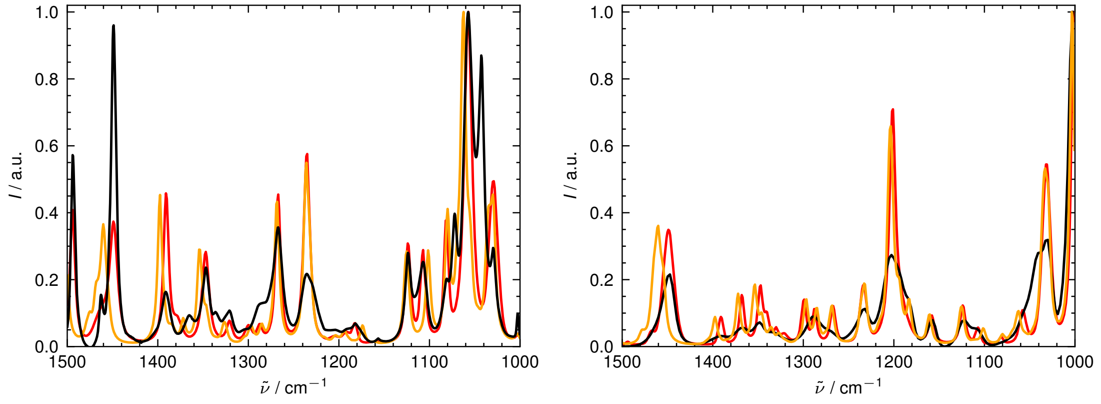

Figure S58: Aligned spectrum of compound **4**, isomer 0. Left: IR, Right: Raman.

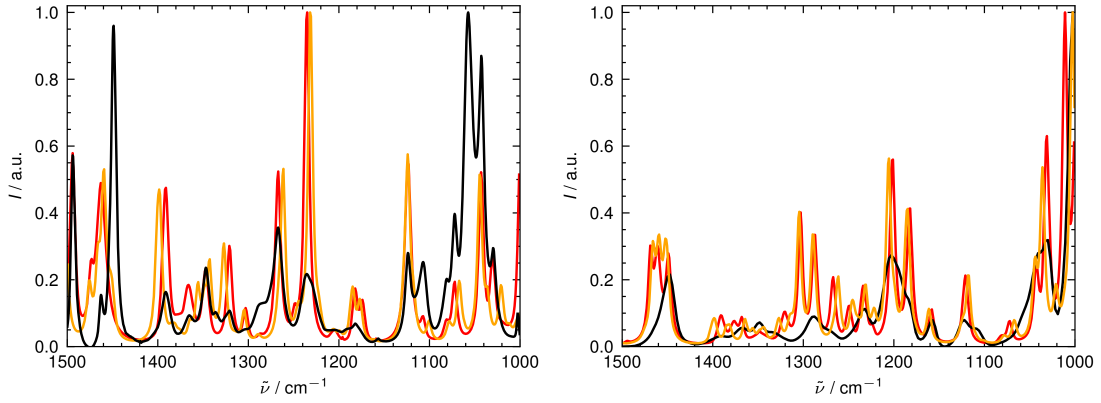

Figure S59: Aligned spectrum of compound **4**, isomer 1. Left: IR, Right: Raman.

3.5 Compound 5

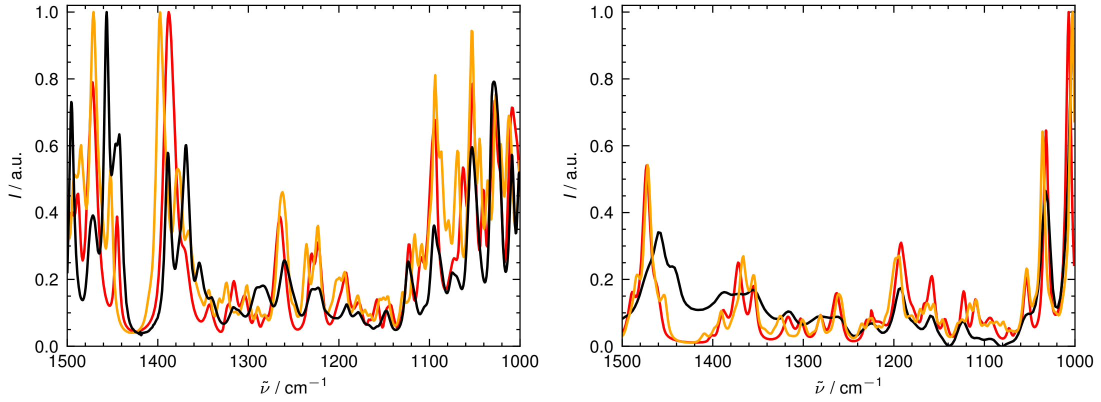

Figure S60: Aligned spectrum of compound **5**, isomer 0. Left: IR, Right: Raman.

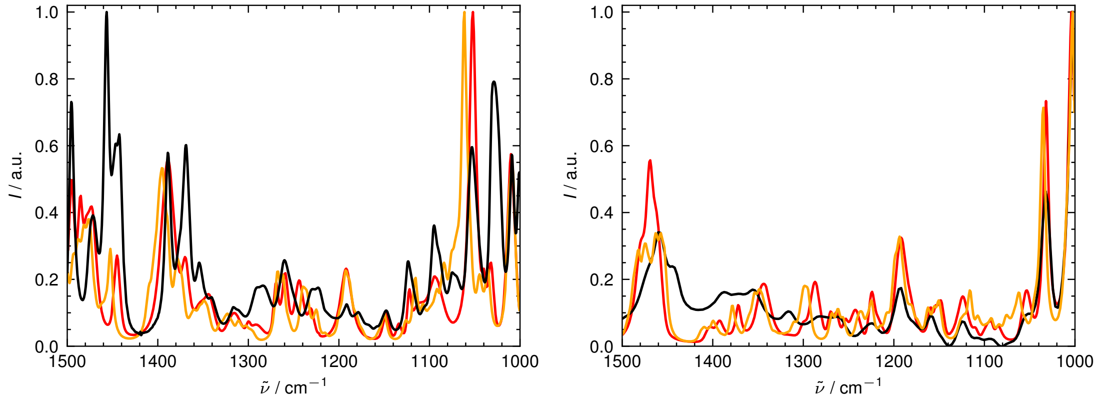

Figure S61: Aligned spectrum of compound **5**, isomer 1. Left: IR, Right: Raman.

3.6 Compound 6

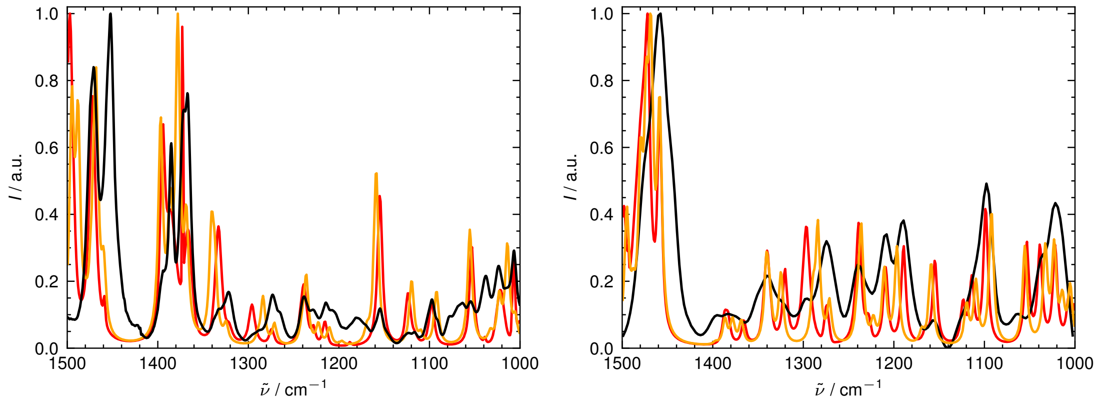

Figure S62: Aligned spectrum of compound **6**, isomer 0. Left: IR, Right: Raman.

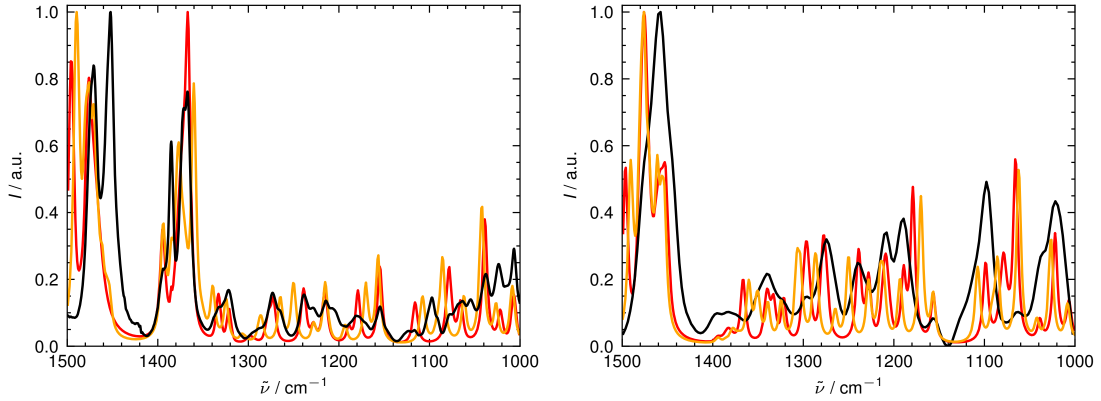

Figure S63: Aligned spectrum of compound **6**, isomer 1. Left: IR, Right: Raman.

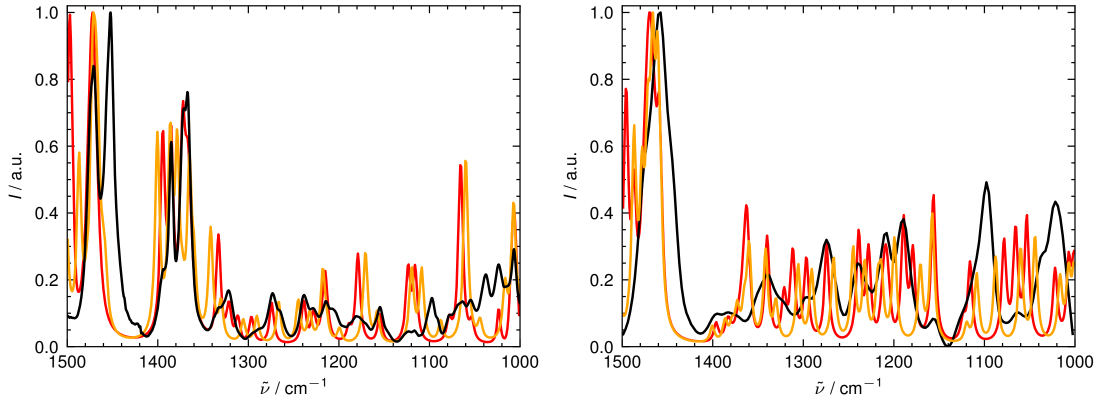

Figure S64: Aligned spectrum of compound **6**, isomer 2. Left: IR, Right: Raman.

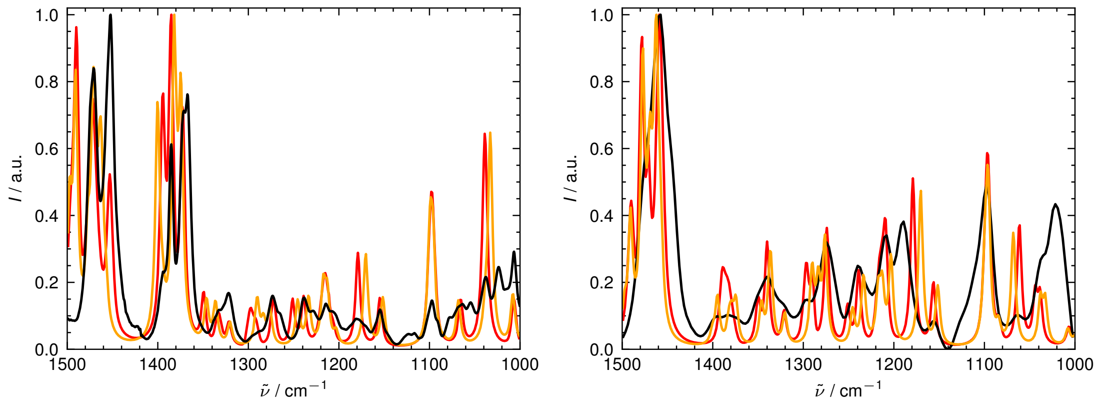

Figure S65: Aligned spectrum of compound **6**, isomer 3. Left: IR, Right: Raman.

3.7 Compound 7

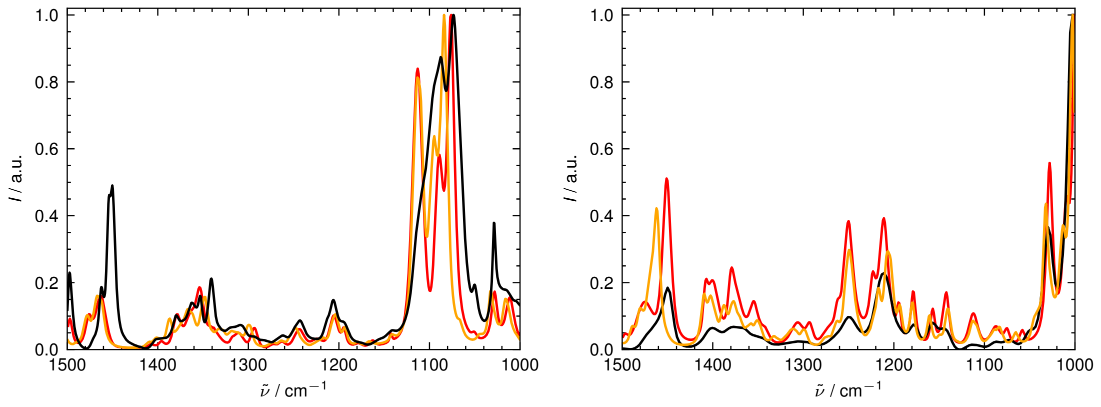

Figure S66: Aligned spectrum of compound **7**, isomer 0. Left: IR, Right: Raman.

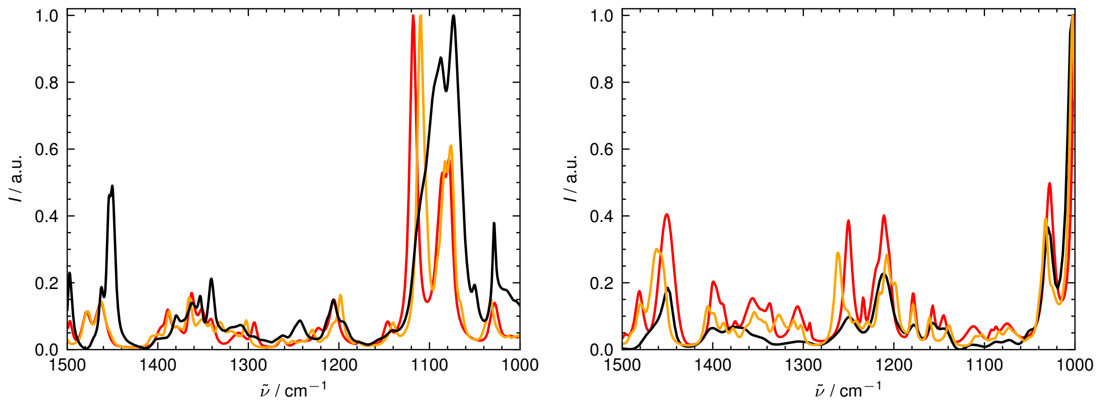

Figure S67: Aligned spectrum of compound **7**, isomer 1. Left: IR, Right: Raman.

3.8 Compound 8

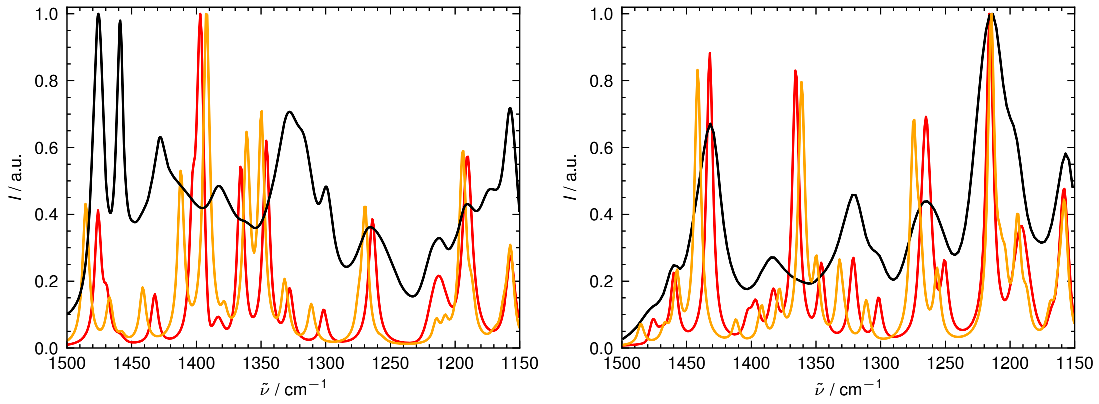

Figure S68: Aligned spectrum of compound **8**, isomer 0. Left: IR, Right: Raman.

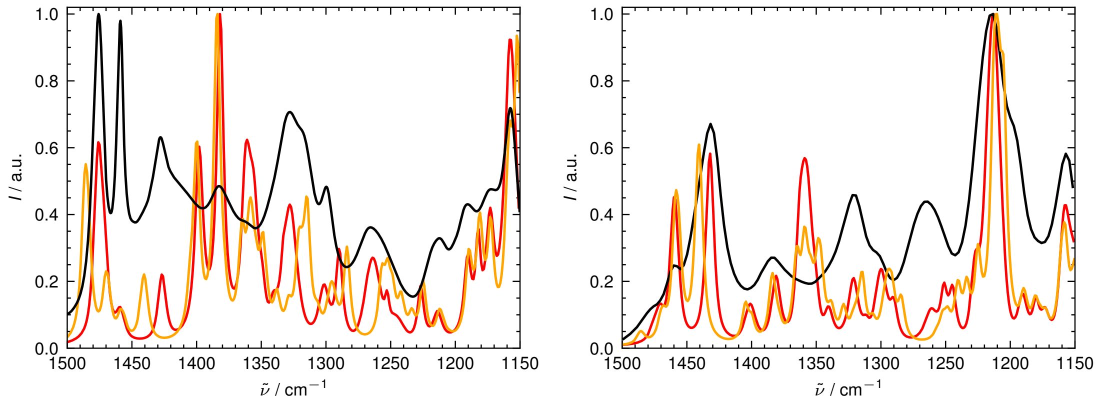

Figure S69: Aligned spectrum of compound **8**, isomer 1. Left: IR, Right: Raman.

3.9 Compound 9

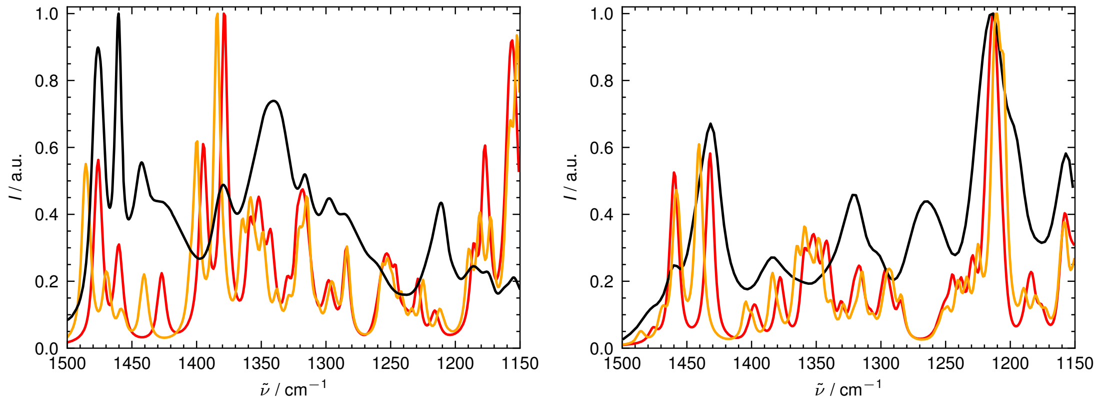

Figure S70: Aligned spectrum of compound **9**, isomer 0. Left: IR, Right: Raman.

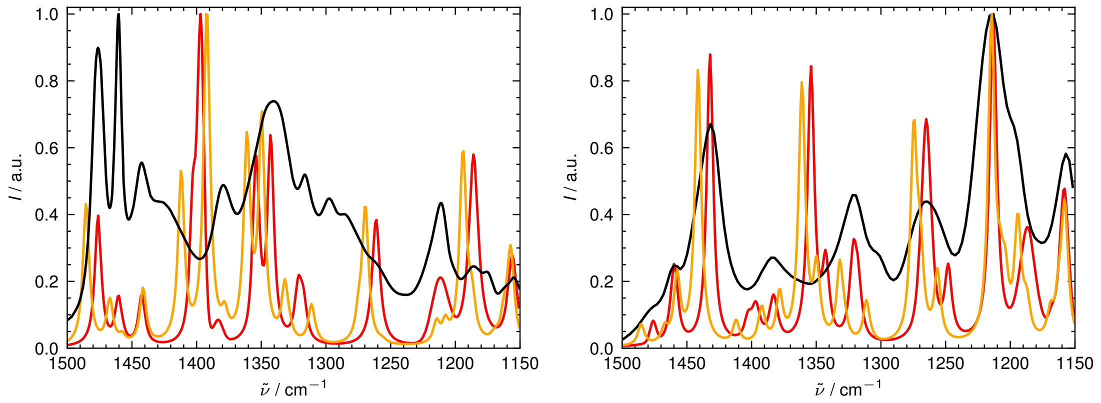

Figure S71: Aligned spectrum of compound **9**, isomer 1. Left: IR, Right: Raman.

3.10 Compound 10

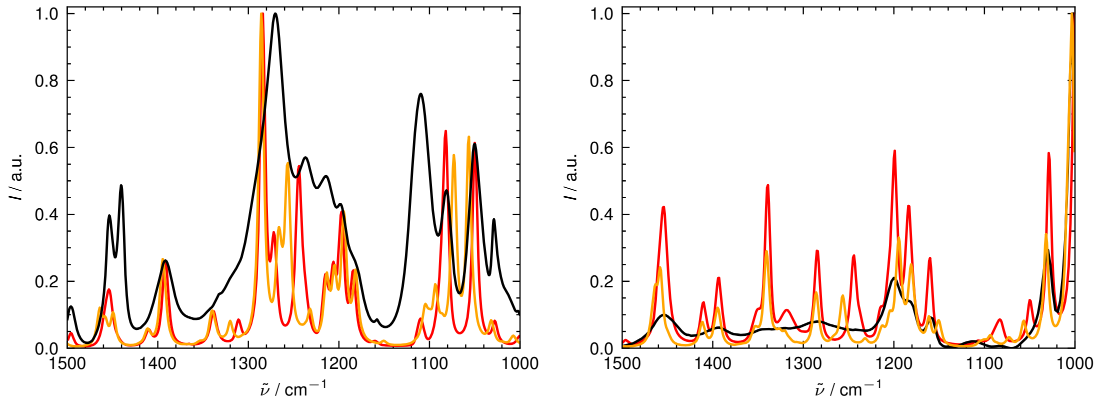

Figure S72: Aligned spectrum of compound **10**, isomer 0. Left: IR, Right: Raman.

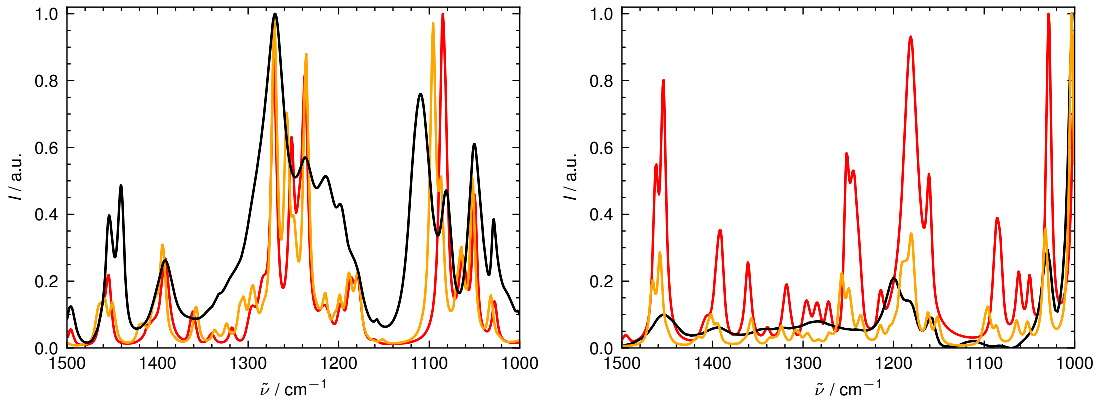

Figure S73: Aligned spectrum of compound **9**, isomer 1. Left: IR, Right: Raman.

3.11 Compound 11

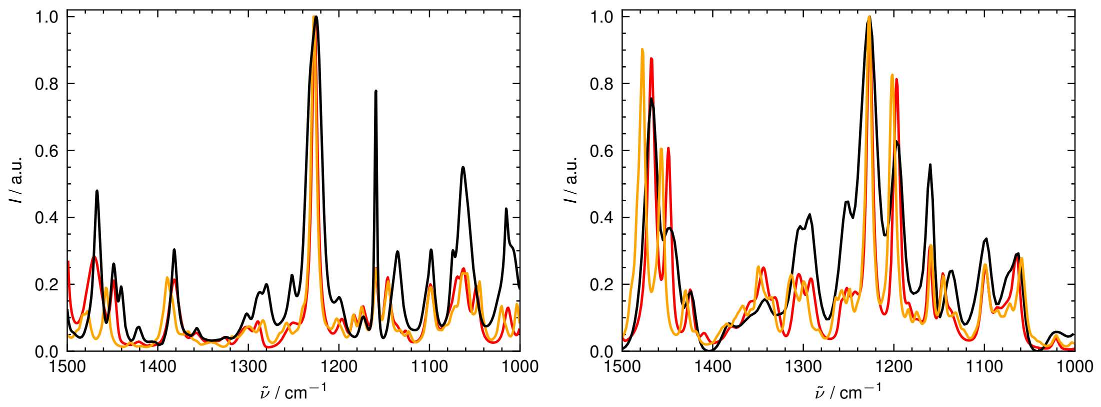

Figure S74: Aligned spectrum of compound **11**, isomer 0. Left: IR, Right: Raman.

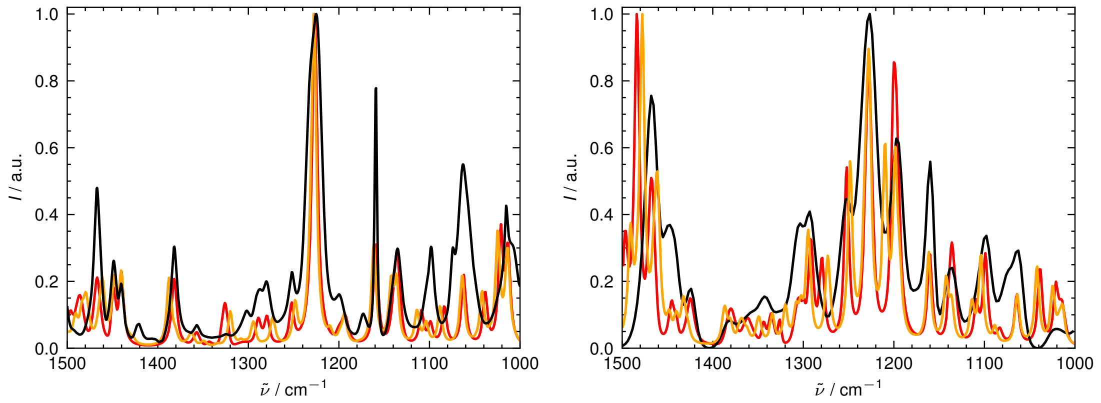

Figure S75: Aligned spectrum of compound **11**, isomer 1. Left: IR, Right: Raman.

3.12 Compound 12

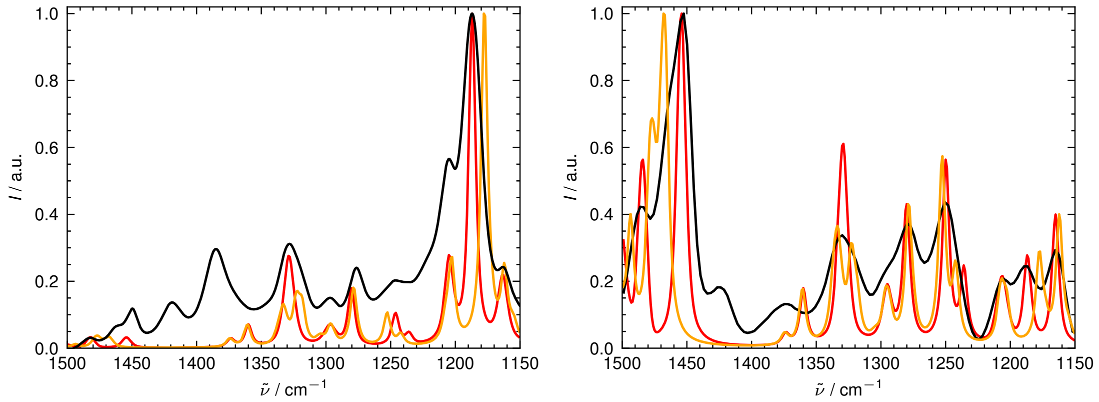

Figure S76: Aligned spectrum of compound **12**, isomer 0. Left: IR, Right: Raman.

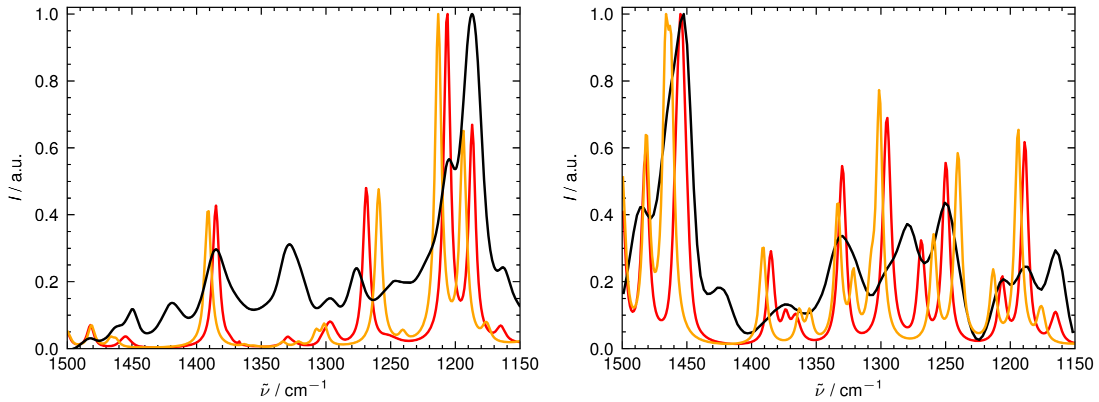

Figure S77: Aligned spectrum of compound **12**, isomer 1. Left: IR, Right: Raman.

3.13 Compound 13

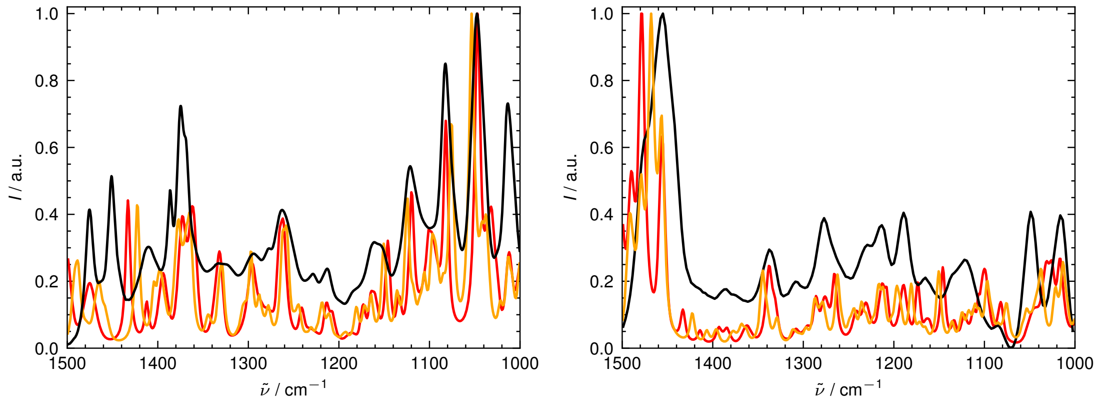

Figure S78: Aligned spectrum of compound **13**, isomer 0. Left: IR, Right: Raman.

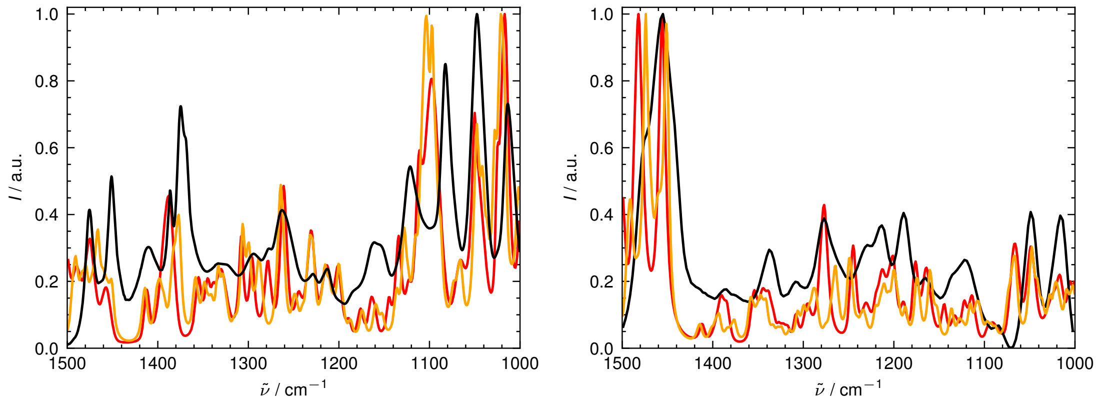

Figure S79: Aligned spectrum of compound **13**, isomer 1. Left: IR, Right: Raman.

3.14 Compound 14

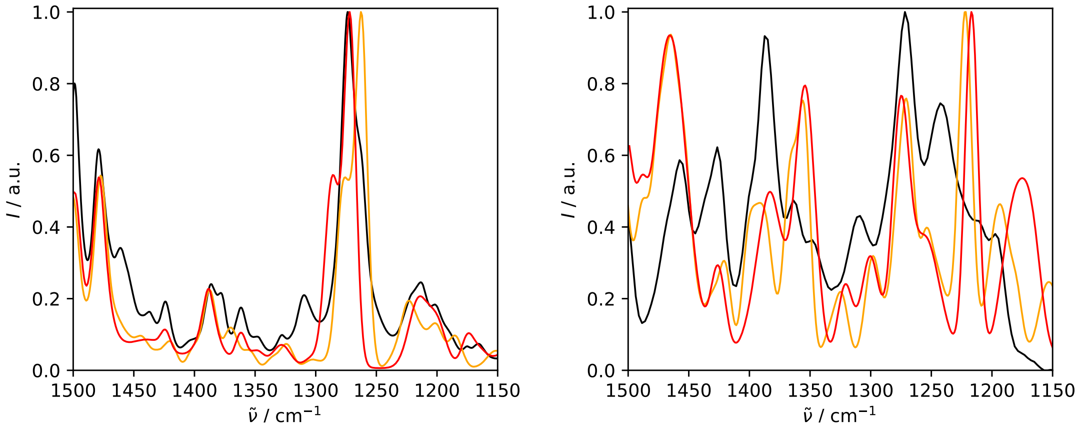

Figure S80: Aligned spectrum of compound **14**, isomer 0. Left: IR, Right: Raman.

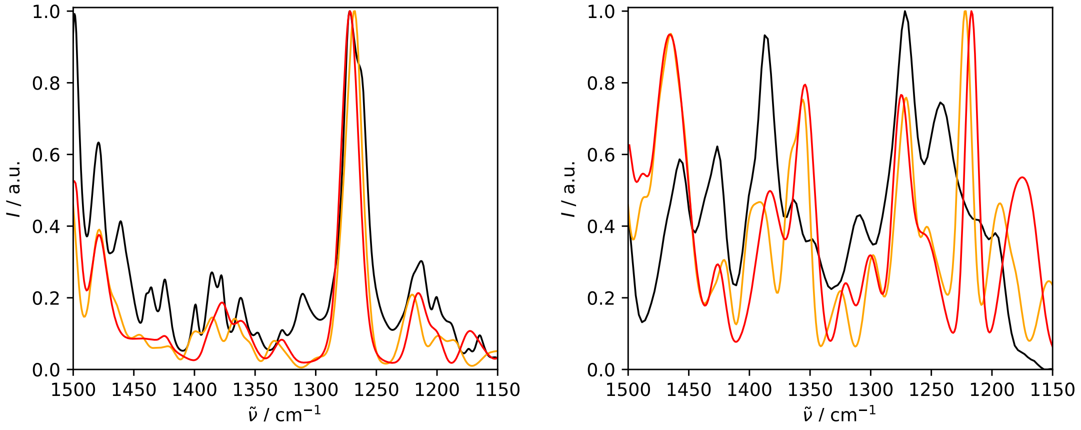

Figure S81: Aligned spectrum of compound **14**, isomer 1. Left: IR, Right: Raman.

3.15 Compound 15

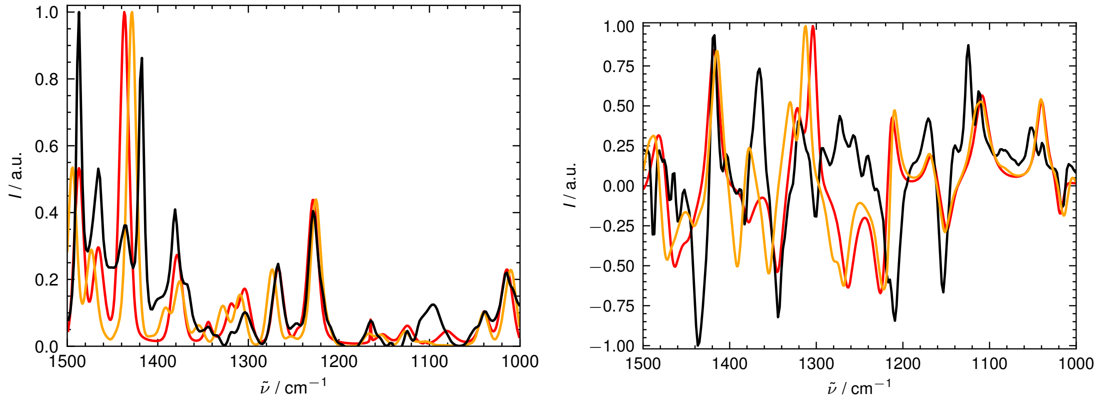

Figure S82: Aligned spectrum of compound **15**, isomer 0. Left: IR, Right: VCD.

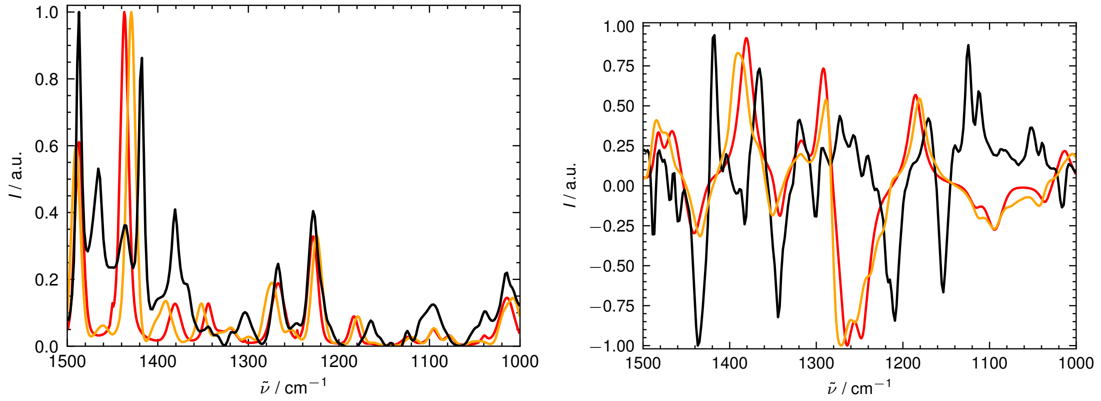

Figure S83: Aligned spectrum of compound **15**, isomer 1. Left: IR, Right: VCD.

3.16 Compound 16

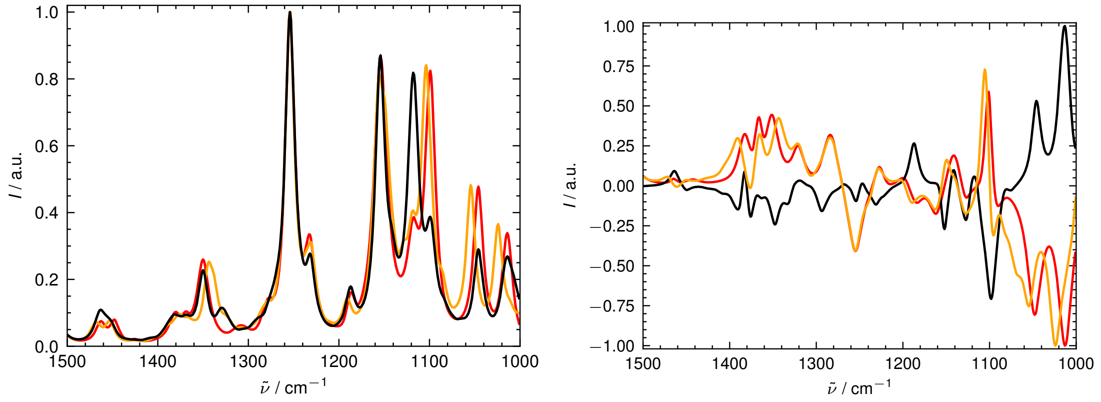

Figure S84: Aligned spectrum of compound **16**, isomer 0. Left: IR, Right: VCD.

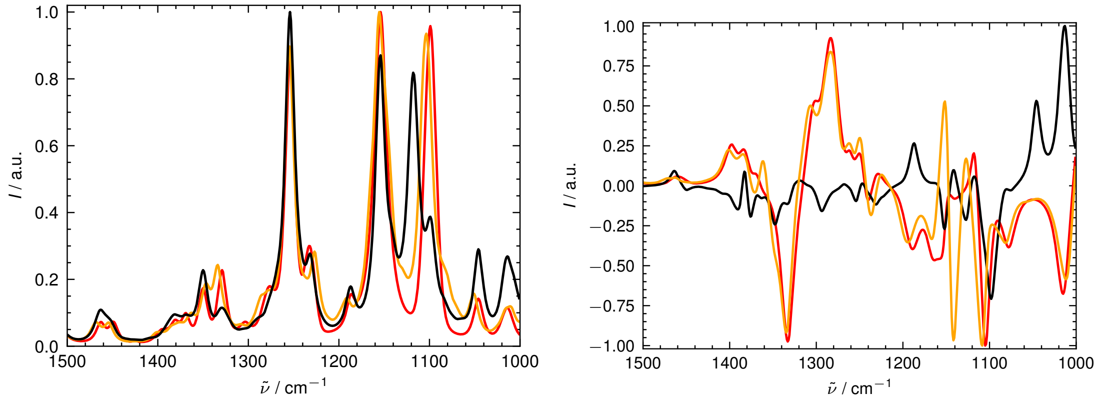

Figure S85: Aligned spectrum of compound **16** isomer 1. Left: IR, Right: VCD.

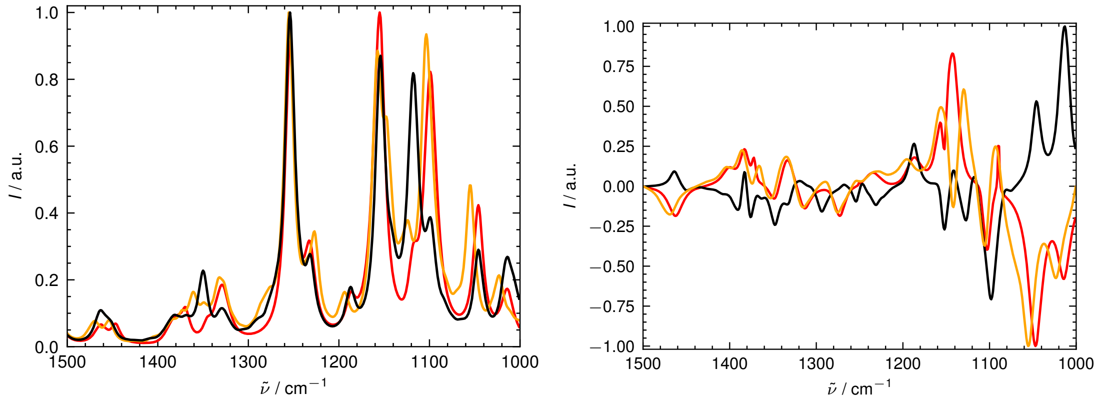

Figure S86: Aligned spectrum of compound **16** isomer 2. Left: IR, Right: VCD.

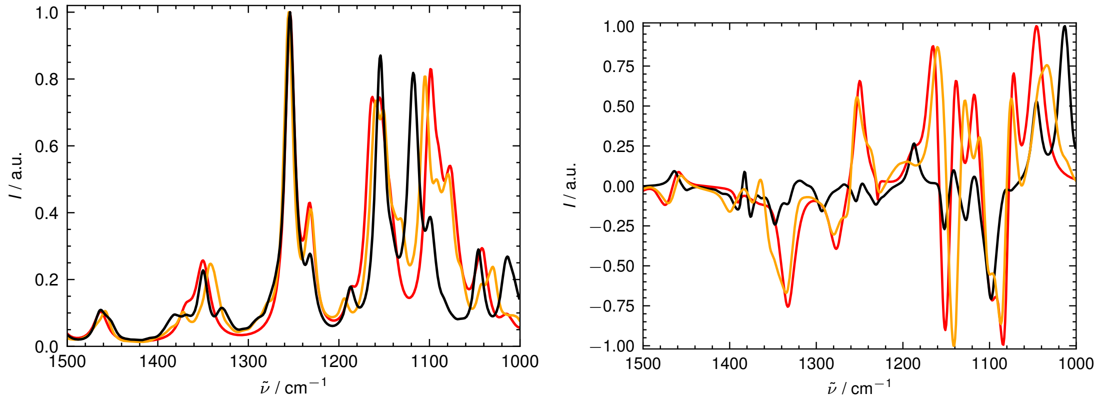

Figure S87: Aligned spectrum of compound **16** isomer 3. Left: IR, Right: VCD.

3.17 Compound 17

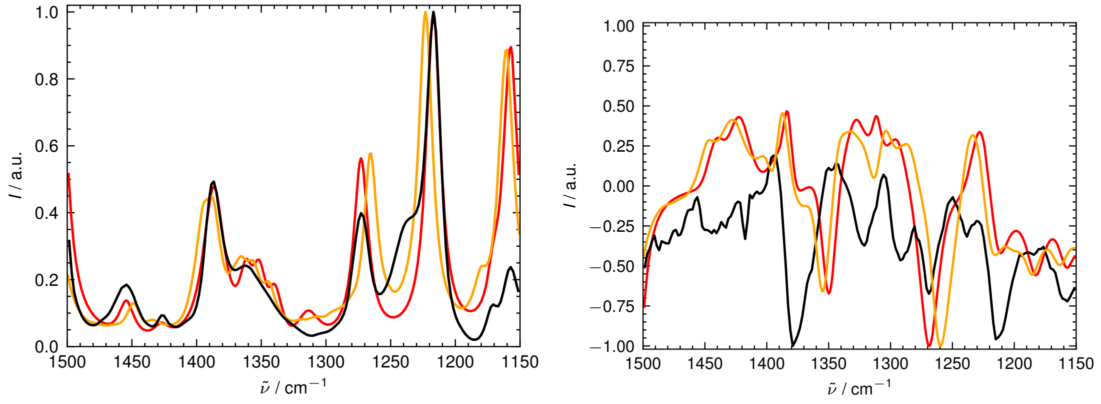

Figure S88: Aligned spectrum of compound **17**, isomer 0. Left: IR, Right: VCD.

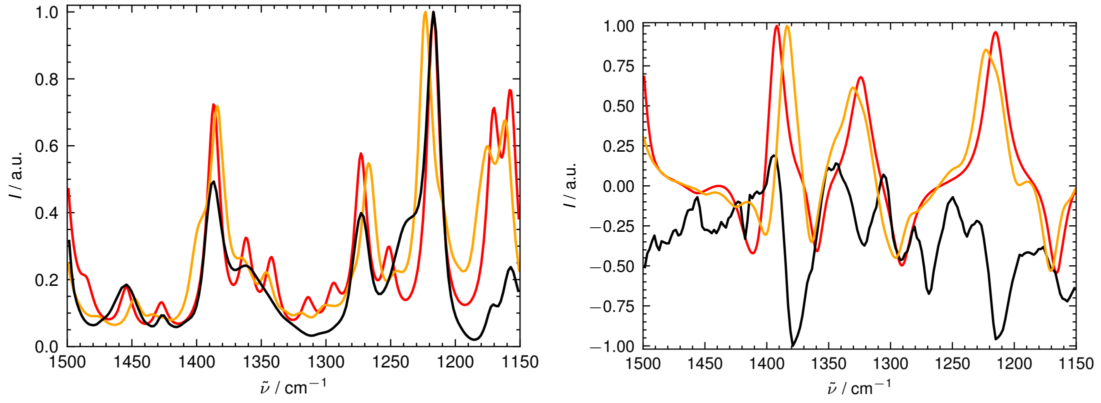

Figure S89: Aligned spectrum of compound **17** isomer 1. Left: IR, Right: VCD.

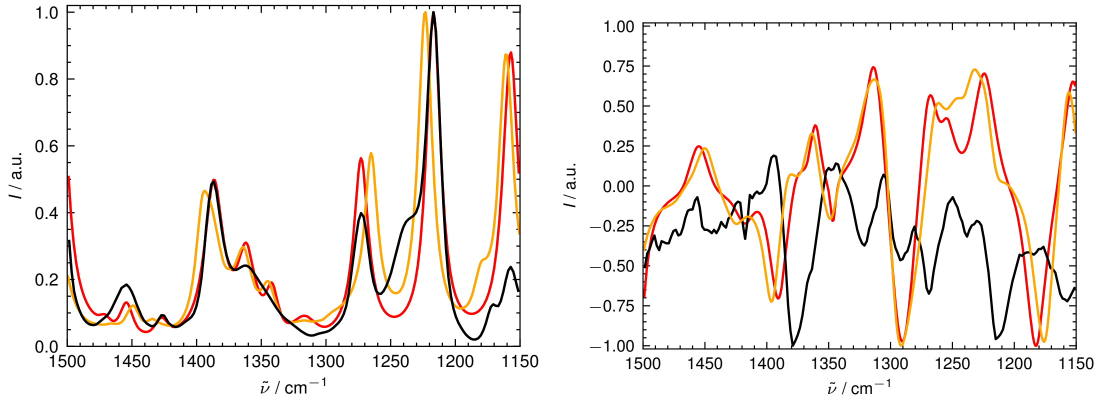

Figure S90: Aligned spectrum of compound **17** isomer 2. Left: IR, Right: VCD.

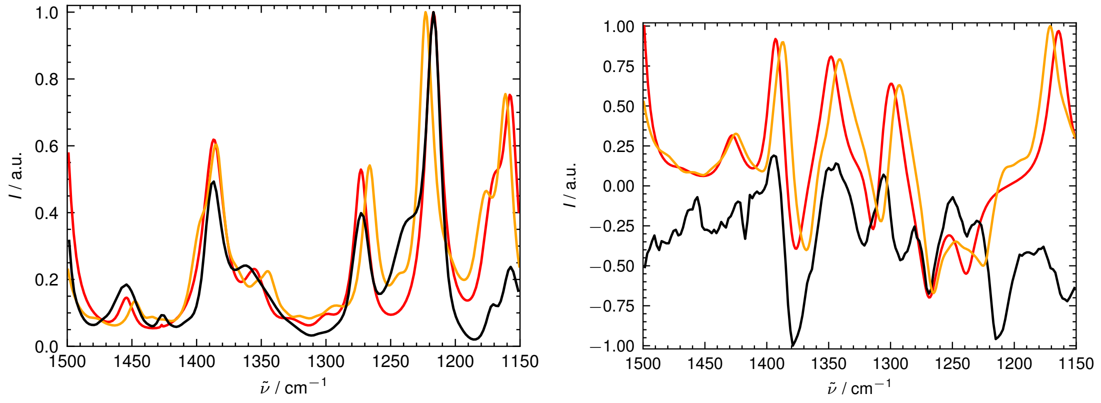

Figure S91: Aligned spectrum of compound **17** isomer 3. Left: IR, Right: VCD.
